# Association between night shift work, genetic risk, and chronic kidney disease: a prospective cohort study based on the UK Biobank

**DOI:** 10.3389/fpubh.2026.1772746

**Published:** 2026-04-30

**Authors:** Qianchun Xu, Jiayao Fan, Zhiyi Chen, Duo Lv, Dan Zhou, Zhangfei Shou, Xishao Xie

**Affiliations:** 1 Zhejiang Chinese Medical University, Hangzhou, Zhejiang, China; 2 Department of Nephrology, Shulan Hangzhou Hospital Affiliated to Zhejiang Shuren University Shulan International Medical College, Key Laboratory of Artificial Organs and Computational Medicine in Zhejiang Province, Hangzhou, China; 3 School of Public Health, The Second Affiliated Hospital Zhejiang University School of Medicine, Hangzhou, China; 4 Zhejiang University School of Medicine, Hangzhou, China; 5 Department of Clinical Pharmacy, The First Affiliated Hospital of Zhejiang University School of Medicine, Hangzhou, China; 6 Kidney Disease Center, The First Affiliated Hospital of Zhejiang University School of Medicine, Hangzhou, China

**Keywords:** chronic kidney disease, circadian rhythm, genetic susceptibility, night shift work, occupational health

## Abstract

**Objective:**

To investigate the association between night shift work and chronic kidney disease (CKD) risk and evaluate the interacting role of genetic susceptibility.

**Methods:**

This prospective cohort study included 252,425 participants from the UK Biobank for current night shift analysis and 67,097 for lifetime exposure. Cox proportional hazards models assessed the relationship between various shift patterns and CKD incidence. Formal interaction analyses (additive and multiplicative scales) and sensitivity analyses were conducted. Reporting followed STROBE guidelines.

**Results:**

Compared to those who never or rarely worked night shifts, permanent night shift workers had a significantly higher CKD risk (HR 1.19, 95% CI 1.06–1.34). Lifetime analyses revealed significant associations between incident CKD risk and duration, frequency, shift length, and consecutive shifts. Participants with high genetic risk and permanent night shift exposure had the highest CKD risk (HR 2.49, 95% CI 2.07–2.99). Potential interactions between genetic susceptibility and specific night shift patterns were suggested (*P* < 0.05). Sensitivity analyses further confirmed the robustness of these associations.

**Conclusions:**

Night shift work—particularly permanent schedules and adverse shift patterns—increases the risk of CKD, especially among individuals with high genetic predisposition. Optimizing shift schedules may help reduce CKD incidence.

## Introduction

Chronic kidney disease (CKD) is now widely acknowledged as a pressing issue in global public health, contributing substantially to premature mortality, compromised wellbeing and mounting healthcare expenses (1). Affecting more than 10% of people globally, CKD is projected to rank as the fifth leading cause of mortality by 2040 (1–4). Its pathogenesis arises from genetic predispositions and environmental triggers, whereas modifiable drivers including suboptimal diabetes/hypertension control and unhealthy lifestyle patterns critically accelerate disease progression (5–7). Given its profound societal impact and frequent diagnostic delays, identifying preventable risk factors has emerged as a critical public health imperative.

The growing prevalence of shift work, particularly night shifts, has become a global occupational health concern (8). Approximately 21% European workers and 17–20% United States adults engage in non-traditional work schedules extending beyond conventional daytime hours (9, 10). Night shift employment induces chronic circadian misalignment, disrupting metabolic homeostasis and hormonal regulation (11). These disturbances increase the risks of type 2 diabetes, cardiovascular diseases, hypertension, and psychological disorders (12–15). Beyond its classification as “a probable human carcinogen” by the International Agency for Research on Cancer (IARC) (16), accumulating epidemiological data have consistently shown that prolonged night shift work elevates risks for both breast and prostate cancers (17, 18). Growing evidence indicates that night shift work–related disruption of circadian rhythms may exert detrimental effects on renal health (19). However, its epidemiological association with incident chronic kidney disease remains inconclusive.

The objective of this study was to investigate the association between night shift work and the risk of incident CKD within a population of 252,425 UK Biobank participants. Consideration was given to various temporal exposure characteristics and genetic risk components. It was hypothesized that both current and lifetime night shift exposure are associated with an increased risk of CKD, and that this risk is further modified by genetic predisposition.

## Methods

### Study design and population

**Figure 1:**
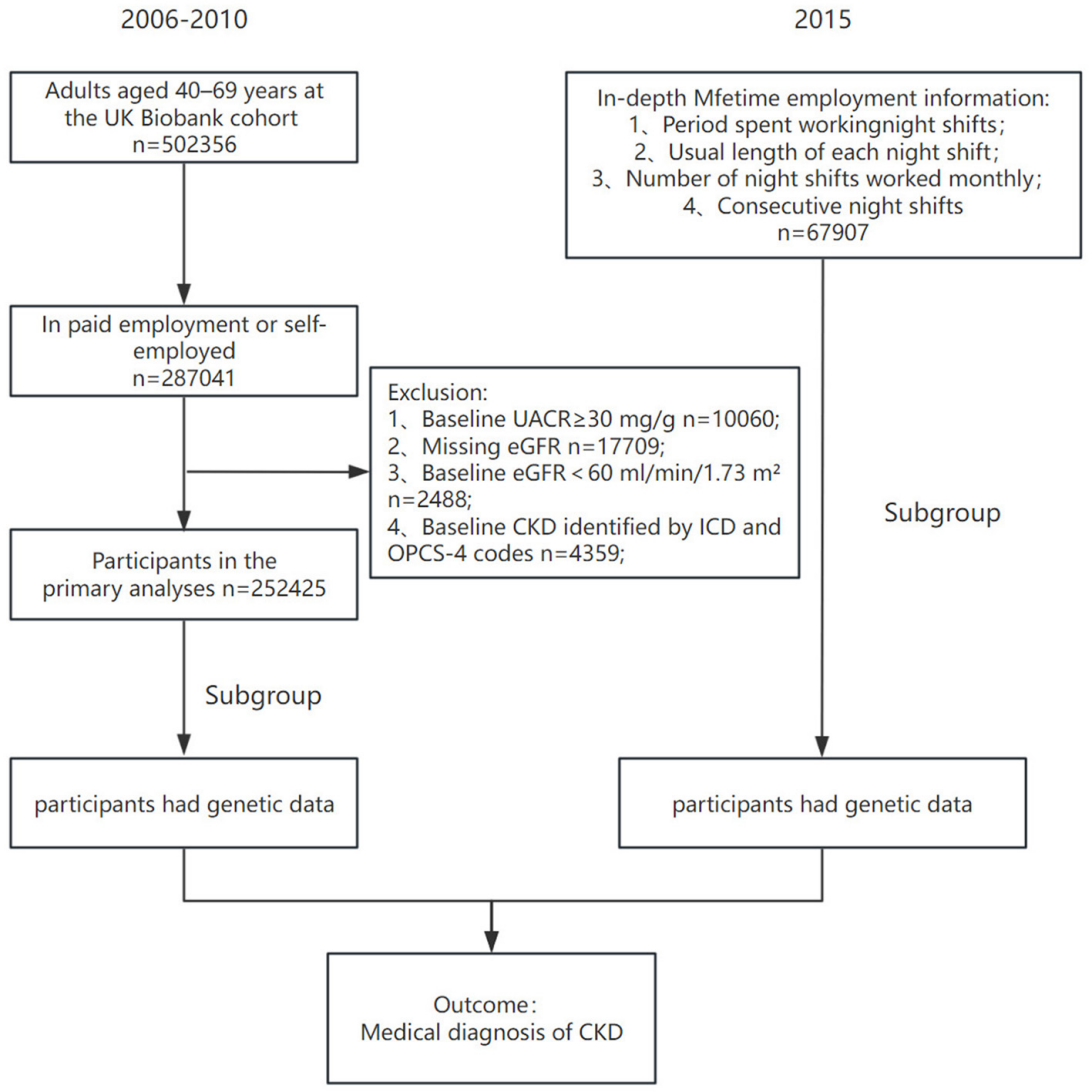
Study participant flow. CKD, chronic kidney disease; UACR, urinary albumin-to-creatinine ratio; eGFR, estimated glomerular filtration rate; PRS, polygenic risk score.

A prospective cohort study was conducted using the UK Biobank as the primary data source. Between 2006 and 2010, this prospective study recruited a population-based sample of over 500,000 individuals in the UK, with ages ranging from 37 to 73 years. The study design focused on baseline-employed participants (employed/self-employed) to evaluate the temporal relationship between night shift work and incident CKD. After excluding individuals with prevalent CKD or missing estimated glomerular filtration rate (eGFR) data, the study size was determined by the maximum available participants meeting the eligibility criteria, yielding a final analytical cohort of 252,425 individuals with complete genetic datasets. Furthermore, participants who had reported employment information at recruitment (2006–2010) and provided a valid email address were invited to complete an additional web-based lifetime occupational history questionnaire in 2015. Consequently, a subset of 75,391 participants who provided comprehensive and codable occupational histories was included in subsequent analyses (Figure 1). Ethical approval for the study protocol was obtained from the relevant institutional review board (20).

### Shift work assessment

Employment characteristics were self-reported via touchscreen interface, capturing current work status (employed/self-employed), shift work patterns, and night shift inclusion. For this study, shift work encompassed all occupational schedules differing from the standard daytime work pattern (9 a.m. to 5 p.m.). This may include shifts during the afternoon, evening, or night, as well as rotating schedules that alternate between these time periods. Participants were given response options of “Never/Rarely,” “Sometimes,” “Usually,” and “Always.” Additionally, night shifts refer to a work schedule that requires employees to work during typical sleeping hours, such as between midnight and 6 a.m. This type of shift disrupts the standard sleep cycle and is common in industries that operate around the clock. Based on their responses, participants' shift work status was categorized into groups: “never/rarely night shifts,” “irregular night shift,” and “permanent night shifts” (Supplementary Table S1). The lifetime occupational history questionnaire captured comprehensive job-specific details for all previous employment, including “period spent working night shifts,” “number of night shifts worked monthly,” “usual length of each night shift,” and “frequency of consecutive night shifts,” allowing for a comprehensive analysis of night shift exposure.

### Ascertainment of CKD

CKD cases were identified through either diminished eGFR (<60 mL/min/1.73 m^2^, derived from CKD-EPI) or urinary albumin-to-creatinine ratio (UACR; ≥30 mg/g). Incident CKD was identified through primary care data, hospital inpatient records, death registry entries and laboratory examinations, using ICD and OPCS-4 codes (Supplementary Table S2).

Follow-up time was calculated from enrollment until first CKD event, mortality, censoring, or study conclusion (Wales: May 2022; Scotland: August 2022; England: October 2022). In the Cox models, individuals who were lost to follow-up or died from other causes were censored at the date of their last recorded contact or death.

### Genetic risk assessment

The polygenic risk score (PRS) quantifying genetic susceptibility to CKD was generated from the UK Biobank's imputed genotype dataset. Following established genotyping and imputation protocols (21), quality control procedures preserved single nucleotide polymorphisms (SNPs) exhibiting a minor allele frequency (MAF) greater than 0.01 and a Hardy-Weinberg equilibrium *P*-value exceeding 1 × 10^−6^. Of 40,042 SNPs previously associated with renal function derived from a large-scale multi-ethnic genome-wide association study (GWAS) meta-analysis (22), 38,508 were successfully mapped to the post-QC dataset after allele harmonization and alignment with the GWAS reference panel (Data Sheet 2). SNP-level summary statistics, including effect size (β), standard error (SE), effect allele frequency (EAF), and *P*-value, were obtained from the aforementioned multi-ethnic GWAS. The PRS was calculated as a weighted sum: PRS = Σ_i_ (β_i_ × G_i_).

Where G_i_ represents the dosage of the effect allele (0, 1, or 2) for SNP_i_, and β_i_ denotes the corresponding GWAS-derived effect size (22). To address ancestry handling and minimize potential population stratification, we included self-reported ethnicity as a covariate in all Cox proportional hazards models. This approach was employed to adjust for ancestral diversity and to enhance the robustness of the observed associations between PRS and CKD risk. Regarding score construction, as the original GWAS weights were derived for increased eGFR, a higher PRS numerically reflects better renal function. To align with clinical risk interpretation, we employed reverse stratification: the “high genetic risk” group was defined as the lowest 20% of the PRS distribution, representing the highest predisposition to CKD. The Low genetic risk group comprised the highest 20% (the top quintile), representing the most protective genetic profile, while the remaining 60% (2nd−4th quintiles) formed the Intermediate genetic risk group.

### Covariate measurement

Baseline covariates were obtained through a combination of self-reported touchscreen questionnaires, standardized physical measurements, central laboratory analysis, and linkage to electronic health records. Demographic and lifestyle factors—including age, sex, race (White/Black/Asian/Other), Townsend deprivation index, education level (degree or above/other qualifications), smoking status (never/former/current), alcohol status (never/former/current), sleep duration, labor work level (never/sometimes/often/always)—were collected via baseline questionnaires. To evaluate both linear and non-linear associations, quantitative variables were handled as either continuous or categorical factors. Healthy diet score was calculated based on daily intake (Daily: ≥4 tbsp vegetables, ≥3 servings fruit. Weekly: ≥2 fish meals, ≤ 1 processed meat, ≤ 2 unprocessed red meats). Anthropometric data, specifically body mass index (BMI), were derived from standardized physical measurements and categorized into four groups according to the World Health Organization (WHO) international classification to identify specific risk thresholds: underweight (<18.5 kg/m^2^), normal (18.5 to <25 kg/m^2^), overweight (25 to <30 kg/m^2^), and obese (≥30 kg/m^2^). Clinical and biochemical factors, including baseline eGFR, baseline diagnoses of hypertension, diabetes, cardiovascular disease (CVD) and cancer, were determined through central laboratory analysis and linkage to ICD and OPCS-4 coded health records (Supplementary Table S3).

### Statistical analysis

The proportion of missing values for the covariates was generally low, ranging from 0.09% for alcohol consumption to 11.12% for education. To maintain statistical power and minimize potential bias, Multiple Imputation by Chained Equations (MICE) was employed to handle missing data for all analytical models. Lifetime night shift work categories were defined based on non-linear associations identidied from restricted cubic spline (RCS) models (23–25). Cut-off points were anchored at key inflection points where the Hazard Ratio (HR) exhibited significant shifts or reached a plateau, ensuring that each category represented a distinct risk profile. Specifically, duration was grouped into none, <2, 2–14, and >14 years, reflecting the baseline, the initial risk elevation (~2 years), and the subsequent non-monotonic trend or risk plateau. Monthly frequency (none, <5, 5–10, >10 times) and shift length (none, <8, 8–12, >12 h) and consecutive shifts (none, <2, 2–7, >7 shifts) were similarly categorized by aligning thresholds with visual inflections in the RCS curves to explore joint associations among different exposure dimensions.

Baseline characteristics were summarized as means with standard deviations (SD) for normally distributed variables or medians with interquartile ranges (IQR) for skewed data. These characteristics were compared according to the predefined lifetime night shift work categories and current participants' shift work status, respectively.

Time to first CKD event was assessed using the Kaplan-Meier method, estimating cumulative incidence based on current and lifetime night shift work status, with results illustrated in a survival plot. Associations of night shift work (current and lifetime) with CKD incidence were evaluated via Cox proportional hazards models, with results expressed as HRs and 95% confidence intervals (CIs). The following models were established: (i) an unadjusted model; (ii) “Model 1,” adjusted for gender, age, and race; (iii) “Model 2,” with additional adjustments for BMI, alcohol and smoking status, education, and the Townsend deprivation index; and (iv) “Model 3,” a fully adjusted model, further adjusting for healthy diet score, labor work level, and baseline comorbidities (including hypertension, diabetes, CVD, and cancer). Model 3 was designated as the primary analytical model for all statistical evaluations throughout this study, including the aforementioned RCS analyses used for exploring non-linear associations, as well as mediation analysis and sensitivity analyses. The adequacy of the proportional hazards assumption for the final multivariable model was assessed using Schoenfeld residuals. To provide clinically meaningful measures of absolute risk, incidence rates and 10-year cumulative risks were also calculated. Incidence rates were expressed as the number of CKD cases per 1,000 person-years. The absolute risk increase was defined as the difference in incidence rates between the night shift groups and the reference group. Additionally, the 10-year cumulative incidence of CKD for each category was estimated using Kaplan-Meier survival analysis.

Mediation analysis was performed to assess the extent to which CKD risk was explained by indirect pathways, including chronotype, sleep duration, physical labor intensity, healthy diet score, Townsend deprivation index, and baseline conditions such as cancer, hypertension, diabetes, CVD, along with smoking and alcohol consumption status. Specifically, a causal mediation framework based on the counterfactual approach was employed, utilizing the R “mediation” package. This method allowed for the decomposition of the Total Effect into the Average Causal Mediation Effect (ACME) and the average direct effect. To account for the potential non-normal distribution of the indirect effects, non-parametric bootstrapping with 1,000 iterations was applied to estimate 95% CIs. The Proportion Mediated was determined as the ratio of the ACME to the Total Effect. Simultaneously, joint models were developed to investigate whether genetic susceptibility influenced the associations between current rotating night shift work, lifetime night shift exposure, and the risk of incident CKD. For these analyses, a common reference group was defined as individuals with low genetic risk and no night shift work. To evaluate the joint associations between genetic susceptibility and night shift work patterns on an additive scale, the Relative Excess Risk due to Interaction (RERI) and the Attributable Proportion due to interaction (AP) were calculated. These metrics were used to explore potential departures from additivity, following the standard formulas: RERI = HR_11_ – HR_10_ – HR_01_ + 1 and AP = RERI/HR_11_. In these formulas, HR_11_ represents the risk of the group with both high genetic risk and night shift work, while HR_10_ and HR_01_ represent the risk of having only one of these factors, all compared against the aforementioned common reference group. To ensure statistical robustness, 95% confidence intervals (CIs) for RERI and AP were estimated via a bootstrapping procedure with 1,000 iterations. These analyses were intended to characterize combined risk profiles rather than to definitively establish biological effect modification.

Sensitivity analyses were carried out to assess the consistency and robustness of the outcomes. Initially, individuals diagnosed with CKD within 24 months of baseline were excluded to mitigate potential reverse causality. Second, participants with incomplete covariate data were excluded to enhance analytical reliability. Third, stratification of the analyses by BMI, race, and other potential confounders listed in the baseline table was performed to minimize the impact of confounding factors. Fourth, aiming to reduce potential biases arising from the limitations of specific algorithms, genetic susceptibility of CKD calculated using alternative method reported by Yu et al. (26), was utilized to repeat the gene association analysis. Fifth, Fine-Gray competing risks regression was employed to address potential bias from mortality. Finally, to mitigate immortal time bias and ensure a clear temporal sequence, employment timelines from the OSCAR questionnaire (Fields 22602 and 22603) were utilized. This involved excluding 26 participants who commenced night shift work after baseline recruitment (2006–2010) and strictly truncating exposure duration at the recruitment year for jobs spanning the baseline period.

Analyses were executed using R version 4.4.1, with statistical significance set at *P* < 0.05 (two-tailed).

## Results

### Study population and participant flow

The participant selection process is illustrated in Figure 1. Among the initial 502,364 UK Biobank participants, 249,939 individuals were excluded due to prevalent CKD at baseline or missing biochemical and genetic data. Consequently, a primary analytical cohort of 252,425 individuals was identified for the analysis of current night shift status. Within this cohort, a sub-cohort of 75,391 participants with complete lifetime occupational history was included to evaluate cumulative exposure parameters.

### Non-linear associations between night shift work and CKD

RCS models revealed significant non-linear associations between night shift exposure metrics and adverse outcomes, identifying critical thresholds for risk stratification. Risk associated with duration escalated significantly at approximately 2 years and reached a peak around 8–9 years, and exhibited a non-monotonic trend with a subsequent plateau or slight decline in the highest exposure categories (Supplementary Figure S1A). Monthly frequency showed a sharp HR increase up to 5–6 shifts before reaching a relative plateau near 10 shifts (Supplementary Figure S1B). Shift length exhibited an inverted U-shaped, non-monotonic pattern, peaking between 3 and 5 h and declining after 8 h (Supplementary Figure S1C). Additionally, the risk associated with consecutive night shifts increased significantly after 2 shifts and reached a relative plateau after approximately 4–5 shifts (Supplementary Figure S1D).

### Baseline characteristics

As shown in Table 1, participants exhibited differential baseline characteristics according to their night shift work status. As specified in the methods, multiple imputation was employed to address missing covariates. Detailed numbers and percentages of missing data for each variable prior to imputation are provided in Supplementary Table S4. The study ultimately included 252,425 participants, 57.1% were female and 94.4% were white. Compared with those who never or rarely participate in night shifts, night shift workers were more often male, have a higher Townsend deprivation index, and were less likely to hold higher educational qualifications. A higher probability of alcohol consumption and elevated BMI values were also exhibited. Furthermore, shorter sleep duration, a greater likelihood of engaging in manual labor, and a higher probability of hypertension diagnosis were observed.

**Table 1:** Baseline characteristics according to categories of current night shift work (*n* = 252,425).

Characteristics	Total	Current work schedule		
		Never/rarely night shifts	Irregular night shift	Permanent night shift
No. participants	252,425	230,296	16,564	5,565
Age (years) ± SD	52.6 ± 7.1	52.8 ± 7.1	51.0 ± 6.8	51.3 ± 6.8
Sex (*n*, %)				
Female	130,518 (51.7)	122,253 (53.1)	6,204 (37.5)	2,061 (37.0)
Male	121,907 (48.3)	108,043 (46.9)	10,360 (62.5)	3,504 (63.0)
Ethnic (*n*, %)				
White	238,258 (94.4)	218,787 (95.0)	14,472 (87.4)	4,999 (89.8)
Asian	5,786 (2.3)	4,897 (2.1)	722 (4.4)	167 (3.0)
Black	4,417 (1.7)	3,351 (1.5)	816 (4.9)	250 (4.5)
Other	3,964 (1.6)	3,261 (1.4)	554 (3.3)	149 (2.7)
Education (*n*, %)				
College or University degree	103,960 (41.2)	98,841 (42.9)	4,207 (25.4)	912 (16.4)
Other	148,465 (58.8)	131,455 (57.1)	12,357 (74.6)	4,653 (83.6)
Townsend deprivation index	−2.1 (−3.6, 0.4)	−2.2 (−3.7, 0.3)	−1.3 (−3.2, 1.8)	−1.2 (−3.1, 1.8)
BMI (*n*, %, kg/m^2^)				
Mean	26.5 (24.0,29.6)	26.4 (23.9, 29.5)	27.5 (24.8, 30.6)	27.8 (25.2, 30.9)
<18.5	1,139 (0.5)	1,061 (0.5)	58 (0.4)	20 (0.4)
18.5–24.9	87,080 (34.5)	81,427 (35.4)	4,354 (26.3)	1,299 (23.3)
25–29.9	107,274 (42.5)	97,400 (42.3)	7,338 (44.3)	2,536 (45.6)
≥30	56,932 (22.6)	50,408 (21.9)	4,814 (29.1)	1,710 (30.7)
Smoking (*n*, %)				
Never	145,169 (57.5)	133,486 (58.0)	8,776 (53.0)	2,907 (52.2)
Previous	80,277 (31.8)	73,568 (31.9)	5,041 (30.4)	1,668 (30.0)
Current	26,979 (10.7)	23,242 (10.1)	2,747 (16.6)	990 (17.8)
Alcohol (*n*, %)				
Never	8,470 (3.4)	7,294 (3.2)	871 (5.3)	305 (5.5)
Previous	6,792 (2.7)	6,028 (2.6)	549 (3.3)	215 (3.9)
Current	237,163 (94.0)	216,974 (94.2)	15,144 (91.4)	5,045 (90.7)
Sleep duration (h)	7.00 (6.0,8.0)	7.0 (7.0, 8.0)	7.0 (6.0, 8.0)	7.0 (6.0, 8.0)
Healthy diet score ± SD	2.8 ± 130	2.8 ± 1.30	2.7 ± 1.33	2.6 ± 1.30
Labor work (*n*, %)				
Never	164,367 (65.1)	158,041 (68.6)	5,060 (30.5)	1,266 (22.7)
Sometime	54,140 (21.4)	45,391 (19.7)	6,690 (40.4)	2,059 (37.0)
Usually	17,151 (6.8)	13,618 (5.9)	2,542 (15.3)	991 (17.8)
Always	16,767 (6.6)	13,246 (5.8)	2,272 (13.7)	1,249 (22.4)
Cancer (*n*, %)	20,697 (8.2)	19,221 (8.3)	1,103 (6.7)	373 (6.7)
Hypertension (*n*, %)	54,638 (21.6)	49,474 (21.5)	3,847 (23.2)	1,317 (23.7)
Diabetes (*n*, %)	9,178 (3.6)	8,102 (3.5)	815 (4.9)	261 (4.7)
Cardiovascular disease (*n*, %)	9,826 (3.9)	8,876 (3.9)	704 (4.3)	246 (4.4)
eGFR (mL/min per 1.73 m^2^)	96.0 (86.8, 102.9)	95.9 (86.7, 102.8)	97.5 (88.1, 104.5)	97.2 (87.8, 104.1)

Data were presented as frequency (%), mean ± standard deviation or median (interquartile range).

BMI, body mass index; eGFR, estimated Glomerular Filtration Rate.

### Night shift status and chronic kidney disease risk

During a median follow-up of 13.7 years (IQR, 13.0–14.3 years), 10,573 incident CKD cases were identified among 252,425 participants, contributing to 3.36 million person-years. The overall incidence rates were 3.11, 3.39, and 4.03 per 1,000 person-years for those who never, irregularly, and permanently worked night shifts, respectively (Table 2). Correspondingly, the 10-year cumulative risk of CKD rose from 2.11% in the reference group to 3.42% in the highest exposure group.

**Table 2:** Associations between the current work schedule and risks of CKD.

Variable	Current work schedule			
	Never/rarely night shifts	Irregular night shift	Permanent night shift	
No. participants	230,296	16,564	5,565	
No. cases (*n*, %)	9,533 (4.14)	743 (4.49)	294 (5.28)	
Incidence rate^e^	3.11	3.39	4.03	
10-year cumulative risk^e^ (%)	2.64	3.00	3.42	
	**HR**	**HR (95% CI)**	**HR (95% CI)**	***P*** **-value** ^d^
Unadjusted model	1.00 (ref.)	1.10 (1.01–1.18)	1.29 (1.15–1.45)	<0.001
Model 1^a^	1.00 (ref.)	1.22 (1.13–1.31)	1.42 (1.27–1.60)	<0.001
Model 2^b^	1.00 (ref.)	1.08 (1.00–1.17)	1.22 (1.08–1.37)	<0.001
Model 3^c^	1.00 (ref.)	1.06 (0.98–1.14)	1.19 (1.06–1.34)	0.009

^a^Model 1:adjusted for gender, age and race.

^b^Model 2: adjusted for model 1 plus BMI, alcohol, smoking status, education, and the Townsend deprivation index.

^c^Model 3: adjusted for model 2 plus healthy diet score, labor work level, and baseline diseases (including hypertension, diabetes, CVD, and cancer).

^d^P-values: represent the overall significance of the categorical variable, calculated using the Likelihood Ratio Test (LRT) comparing models with and without the variable.

^e^Incidence rate: expressed as the number of cases per 1,000 person-years.

^f^10-year cumulative risk: estimated absolute probability of incident CKD over a 10-year follow-up period based on Kaplan-Meier analysis.

Unadjusted survival curves showed that individuals with a lifetime history of night shift work had a significantly elevated cumulative incidence of CKD (Supplementary Figure S2). All covariates satisfied the proportional hazards assumption based on Schoenfeld residuals (global test *P* > 0.05). Results from the unadjusted analysis indicated a significant baseline association between night shift status and CKD risk (*P* < 0.001). Compared with participants who rarely or never worked night shifts, the HRs for incident CKD among irregular and permanent night shift workers were 1.22 (95% CI, 1.13–1.31) and 1.42 (95% CI, 1.27–1.60), respectively (Table 2). This association was further examined through three hierarchical adjustment models. After adjusting for basic demographic factors in Model 1, the associations remained significant. Following additional adjustment for lifestyle factors in Model 2, the HRs were modestly attenuated. In the fully adjusted model (Model 3), the positive trend persisted, with an HR of 1.06 (95% CI, 0.98–1.14) for irregular night shift workers and 1.19 (95% CI, 1.06–1.34) for permanent night shift workers, indicating that the increased CKD risk associated with night shift exposure is independent of major clinical confounders.

In further analyses involving the occupational sub-cohort (*n* = 67,907; 2,396 incident CKD cases), four night shift parameters were examined: lifetime duration, monthly frequency, shift length, and consecutive shifts (Supplementary Tables S5–S8). The overall incidence density was 3.15 per 1,000 person-years. The distribution of CKD risk varied across the four occupational parameters (Tables 3–6). Compared with non-night workers, participants with the most cumulative years of night shift work (>14 years) and the highest monthly frequency (>10 shifts/month) exhibited 10-year cumulative risks of 2.88 and 3.26%, respectively. Similarly, the risk reached 3.55% for those with the most consecutive night shifts (>7 shifts/month). In contrast, the relationship with single-shift duration was non-linear; the highest incidence rate (4.21 per 1,000 person-years) and 10-year cumulative risk (4.02%) were recorded in the <8 h per shift group, exceeding the risks observed in groups with longer individual shifts.

**Table 3:** Associations between the period spent working night shifts and risks of CKD.

Variable	Period spent working night shifts				
	None	<2 years	2–14 years	>14 years	
No. participants	51,398	6,148	8,448	1,913	
No. cases (*n*, %)	1,692 (3.29)	255 (3.92)	369 (4.16)	80 (4.18)	
Incidence rate^e^	2.45	3.09	3.27	2.12	
10-year cumulative risk^e^ (%)	2.07	2.58	2.82	2.88	
	**HR**	**HR (95% CI)**	**HR (95% CI)**	**HR (95% CI)**	***P*** **-value** ^d^
Unadjusted model	1.00 (ref.)	1.26 (1.11–1.44)	1.34 (1.20–1.50)	1.28 (1.02–1.60)	<0.001
Model 1^a^	1.00 (ref.)	1.32 (1.15–1.50)	1.35 (1.21–1.52)	1.29 (1.03–1.61)	<0.001
Model 2^b^	1.00 (ref.)	1.18 (1.03–1.35)	1.21 (1.08–1.36)	1.18 (0.94–1.48)	<0.001
Model 3^c^	1.00 (ref.)	1.17 (1.02–1.34)	1.21 (1.08–1.36)	1.14 (0.91–1.43)	0.004

^a^Model 1:adjusted for gender, age and race.

^b^Model 2: adjusted for model 1 plus BMI, alcohol, smoking status, education, and the Townsend deprivation index.

^c^Model 3: adjusted for model 2 plus healthy diet score, labor work level, and baseline diseases (including hypertension, diabetes, CVD, and cancer).

^d^P-values: represent the overall significance of the categorical variable, calculated using the Likelihood Ratio Test (LRT) comparing models with and without the variable.

^e^Incidence rate: expressed as the number of cases per 1,000 person-years.

^f^10-year cumulative risk: estimated absolute probability of incident CKD over a 10-year follow-up period based on Kaplan-Meier analysis.

**Table 4:** Associations between the number of night shifts worked monthly and risks of CKD.

Variable	Number of night shifts worked monthly				
	None	<5/month	5–10/month	>10/month	
No. participants	51,398	4,542	7,567	4,400	
No. cases (*n*, %)	1,692 (3.29)	178 (3.92)	315 (4.16)	211 (4.78)	
Incidence rate^e^	2.45	2.92	3.11	3.58	
10-year cumulative risk^f^ (%)	2.07	2.38	2.64	3.26	
	**HR**	**HR (95% CI)**	**HR (95% CI)**	**HR (95% CI)**	***P*** **-value** ^d^
Unadjusted model	1.00 (ref.)	1.20 (1.02–1.39)	1.28 (1.13–1.44)	1.47 (1.27–1.69)	< 0.001
Model 1^a^	1.00 (ref.)	1.22 (1.05–1.43)	1.33 (1.18–1.51)	1.44 (1.25–1.66)	< 0.001
Model 2^b^	1.00 (ref.)	1.13 (0.97–1.32)	1.21 (1.07–1.37)	1.25 (1.08–1.44)	< 0.001
Model 3^c^	1.00 (ref.)	1.12 (0.96–1.31)	1.20 (1.06–1.36)	1.23 (1.06–1.42)	0.003

^a^Model 1:adjusted for gender, age and race.

^b^Model 2: adjusted for model 1 plus BMI, alcohol, smoking status, education, and the Townsend deprivation index.

^c^Model 3: adjusted for model 2 plus healthy diet score, labor work level, and baseline diseases (including hypertension, diabetes, CVD, and cancer).

^d^P-values: represent the overall significance of the categorical variable, calculated using the Likelihood Ratio Test (LRT) comparing models with and without the variable.

^e^Incidence rate: expressed as the number of cases per 1,000 person-years.

^f^10-year cumulative risk: estimated absolute probability of incident CKD over a 10-year follow-up period based on Kaplan-Meier analysis.

**Table 5:** Associations between the Usual length of each night shift and risks of CKD.

Variable	Usual length of each night shift				
	None	<8 h	8–12 h	>12 h	
No. participants	51,398	1,825	13,492	1,192	
No. cases (*n*, %)	1,692 (3.29)	102 (5.59)	568 (4.21)	34 (2.85)	
Incidence rate^e^	2.45	4.21	3.14	2.11	
10-year cumulative risk^f^ (%)	2.07	4.02	2.65	1.77	
	**HR**	**HR (95% CI)**	**HR (95% CI)**	**HR (95% CI)**	***P*** **-value** ^d^
Unadjusted model	1.00 (ref.)	1.73 (1.42–2.11)	1.29 (1.17–1.42)	0.86 (0.61–1.21)	< 0.001
Model 1^a^	1.00 (ref.)	1.67 (1.37–2.05)	1.31 (1.19–1.45)	0.96 (0.69–1.35)	< 0.001
Model 2^b^	1.00 (ref.)	1.52 (1.24–1.85)	1.17 (1.06–1.29)	0.93 (0.67–1.31)	< 0.001
Model 3^c^	1.00 (ref.)	1.51 (1.23–1.85)	1.16 (1.05–1.28)	0.94 (0.67–1.32)	< 0.001

^a^Model 1:adjusted for gender, age and race.

^b^Model 2: adjusted for model 1 plus BMI, alcohol, smoking status, education, and the Townsend deprivation index.

^c^Model 3: adjusted for model 2 plus healthy diet score, labor work level, and baseline diseases (including hypertension, diabetes, CVD, and cancer).

^d^P-values: represent the overall significance of the categorical variable, calculated using the Likelihood Ratio Test (LRT) comparing models with and without the variable.

^e^Incidence rate: expressed as the number of cases per 1,000 person-years.

^f^10-year cumulative risk: estimated absolute probability of incident CKD over a 10-year follow-up period based on Kaplan-Meier analysis.

**Table 6:** Associations between the consecutive night shifts and risks of CKD.

Variable	Consecutive night shifts				
	None	<2 shifts/month	2–7 shifts/month	>7 shifts/month	
No. participants	51,398	2,751	12,144	1,614	
No. cases (*n*, %)	1,692 (3.29)	95 (3.45)	524 (4.31)	85 (5.27)	
Incidence rate^e^	2.45	2.56	3.22	3.95	
10-year cumulative risk^f^ (%)	2.07	2.04	2.78	3.55	
	**HR**	**HR (95% CI)**	**HR (95% CI)**	**HR (95% CI)**	***P*** **-value** ^d^
Unadjusted model	1.00 (ref.)	1.05 (0.85–1.29)	1.32 (1.20–1.46)	1.62 (1.30–2.01)	<0.001
Model 1^a^	1.00 (ref.)	1.07 (0.87–1.32)	1.36 (1.24–1.51)	1.52 (1.22–1.89)	<0.001
Model 2^b^	1.00 (ref.)	1.02 (0.83–1.26)	1.22 (1.10–1.35)	1.32 (1.06–1.65)	<0.001
Model 3^c^	1.00 (ref.)	1.02 (0.83–1.26)	1.21 (1.09–1.34)	1.31 (1.05–1.63)	<0.001

^a^Model 1:adjusted for gender, age and race.

^b^Model 2: adjusted for model 1 plus BMI, alcohol, smoking status, education, and the Townsend deprivation index.

^c^Model 3: adjusted for model 2 plus healthy diet score, labor work level, and baseline diseases (including hypertension, diabetes, CVD, and cancer).

^d^P-values: represent the overall significance of the categorical variable, calculated using the Likelihood Ratio Test (LRT) comparing models with and without the variable.

^e^Incidence rate: expressed as the number of cases per 1,000 person-years.

^f^10-year cumulative risk: estimated absolute probability of incident CKD over a 10-year follow-up period based on Kaplan-Meier analysis.

**Figure 2:**
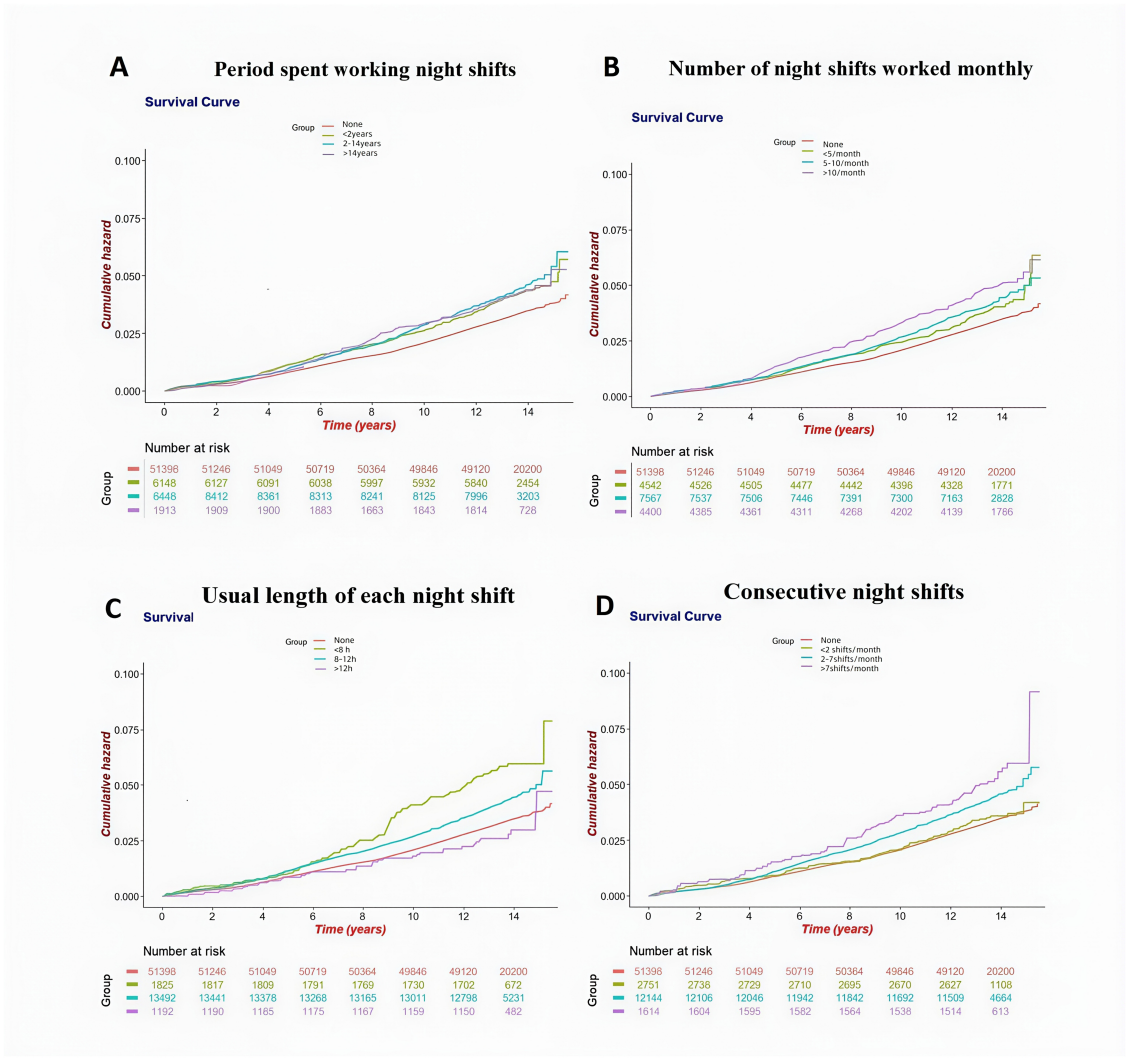
Kaplan-Meier survival curves for incident CKD according to category of Lifelong night shift working status. **(A)** Kaplan-Meier curves for CKD by Period spent working night shifts; **(B)** Kaplan-Meier curves for CKD by Number of night shifts worked monthly; **(C)** Kaplan-Meier curves for CKD by Usual length of each night shift; **(D)** Kaplan-Meier curves for CKD by Consecutive night shifts. Cox models with penalized splines were adjusted for gender, age, race, BMI, alcohol, smoking status, education, the Townsend deprivation index, healthy diet score, labor work level, and baseline diseases (including hypertension, diabetes, CVD, and cancer).

Unadjusted survival curves (Figure 2) and initial Cox models revealed significant baseline associations between all four metrics and elevated CKD risk (*P* < 0.001). Compared with individuals without night shift work, increased risks of incident CKD were observed in participants with 2–14 years of night shift duration (HR 1.21, 95% CI: 1.08–1.36), >10 times/month (HR 1.23, 95% CI: 1.06–1.42), <8 h per shift (HR 1.51, 95% CI: 1.23–1.85), and >7 consecutive night shifts per month (HR 1.31, 95% CI: 1.05–1.63).

In the mediation analysis, chronotype (4.13%, *P* < 0.001) and the Townsend deprivation index (percentage, 7.5%, *P* < 0.001) partially mediated the association between current night shift work and CKD risk. However, no significant mediation effects were observed in relation to sleep duration, physical labor, dietary habits, smoking and drinking behaviors, or comorbidities (cancer, hypertension, diabetes, cardiovascular disease; Supplementary Table S9).

### Night shift status, genetic risk, and CKD risk

**Figure 3:**
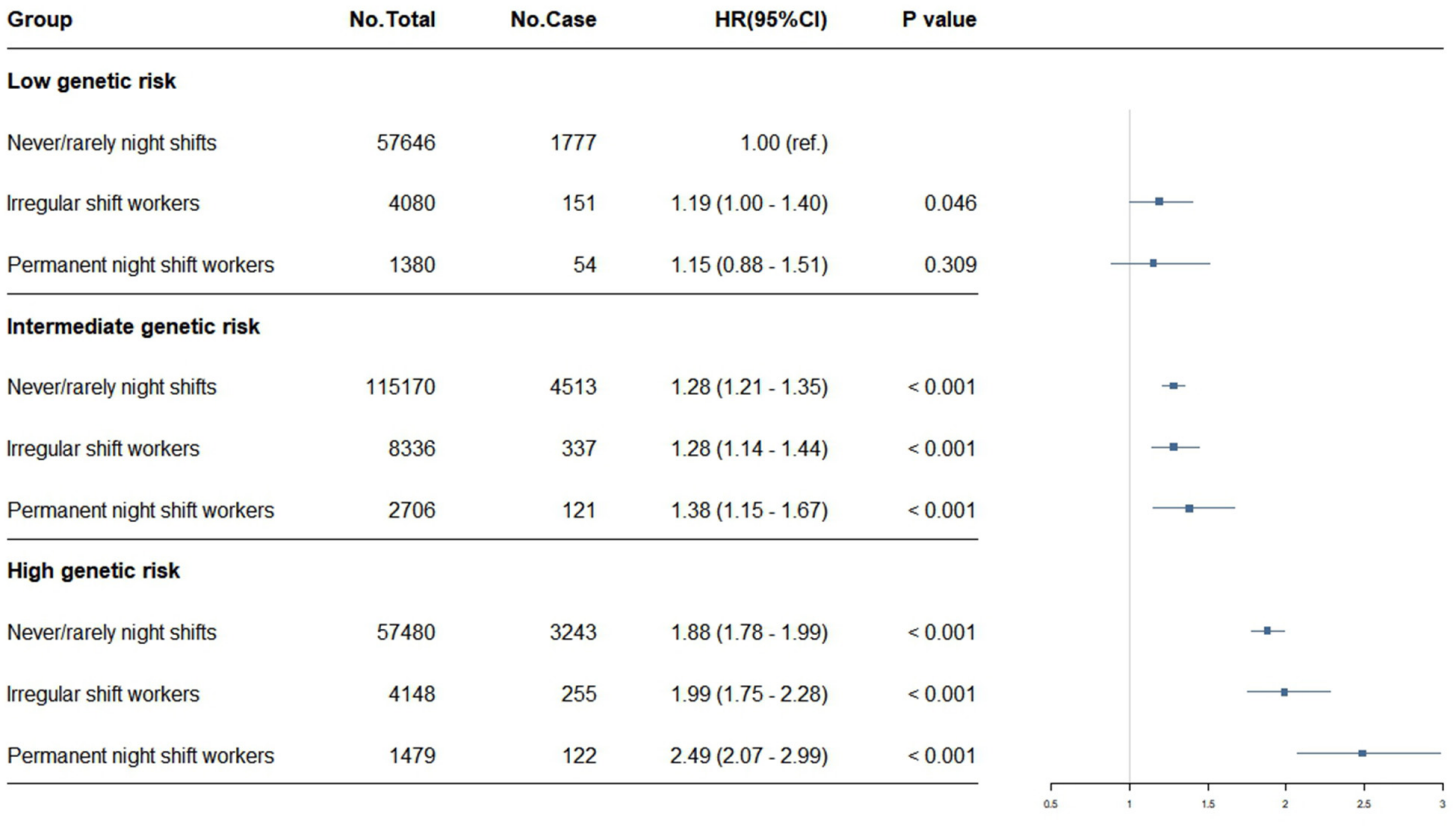
Risk of incident CKD according to current work schedule and genetic risk based on SNPs from Wuttke et al. (22) Cox models were adjusted for gender, age, race, BMI, alcohol, smoking status, education, the Townsend deprivation index, healthy diet score, labor work level, and baseline diseases (including hypertension, diabetes, CVD, and cancer).

Compared to individuals with low genetic risk, those with intermediate or high genetic susceptibility showed a greater likelihood of developing CKD (Supplementary Tables S10, S11). Our joint effects analysis revealed that genetic susceptibility significantly modified the association between night shift work and CKD risk. Figure 3 displays the joint associations between current night work schedules and genetic susceptibility in relation to CKD development. Participants with high genetic susceptibility and permanent night shift work exhibited significantly increased risks of incident CKD (HR 2.49, 95% CI: 2.07–2.99) compared to daytime-only working controls. Highest CKD risks were observed in high genetic risk night shift workers vs. low-risk non-exposed controls across all exposure dimensions: short-term exposure (<2 years) showed HR 2.31 (95% CI: 1.82–2.93), moderate shift frequency (5–10 monthly rotations) showed HR 2.47 (95% CI: 1.98–3.07), extended shift duration (8–12 h) showed HR 2.35 (95% CI: 1.96–2.83), and recurrent consecutive shifts (2–7 monthly) showed HR 2.38 (95% CI: 1.97–2.87; Supplementary Figures S3–S6). This consistent pattern of markedly elevated hazard ratios in genetically susceptible individuals exposed to night shifts demonstrates a strong modifying effect of genetic background.

To further characterize the combined risk profiles and evaluate potential departures from additivity, we performed an analysis of joint associations on an additive scale. The analysis, quantified by RERI and AP, revealed heterogeneous patterns dependent on genetic risk stratification and exposure type (Supplementary Tables S12–S16). Significant super-additive associations (RERI and AP > 0) were specifically identified. For instance, among individuals with a high genetic risk, permanent night shift work exhibited a significant super-additive association (RERI: 0.45, 95% CI: 0.07–0.88; AP: 0.18, 95% CI: 0.04–0.40). Similarly, a joint association exceeding additivity was observed in individuals with an intermediate genetic risk who worked high-frequency night shifts (>10 shifts/month; RERI: 0.71, 95% CI: 0.15–1.18; AP: 0.35, 95% CI: 0.08–0.54). Furthermore, sub-additive associations (RERI and AP <0) were observed in certain intermediate and high genetic risk subgroups. For instance, a sub-additive pattern was identified between high genetic risk and irregular shift work (RERI: −0.08, 95% CI: −0.29 to −0.04).

Taken together, the joint association and additive-scale analyses provide complementary evidence of the combined risk profiles, indicating that the risk of CKD associated with night shift work may vary significantly across different levels of genetic susceptibility.

### Sensitivity analyses

In sensitivity analyses, baseline characteristics of current and lifetime night shift work status were examined after removing participants with incomplete covariate data (Supplementary Tables S17–S21). Further Cox regression analyses showed that the associations remained significant after omitting cases with missing values for covariates (Supplementary Tables S22–S26) or restricting the analysis to incident CKD cases occurring more than two years after enrollment (Supplementary Tables S27–S31). Subgroup analyses further demonstrated consistent associations between current/lifetime night shift work and CKD risk across all predefined strata. Current night shift exposure showed stronger associations in individuals without hypertension (*P*_interaction_ = 0.02) or diabetes (*P*_interaction_ = 0.03). Notably, both current and lifetime night shift exposures exhibited the most pronounced associations in White individuals without cardiovascular disease (all *P*_interaction_ <0.001; Supplementary Tables S32–S36). Moreover, the relationship between genetic predisposition, night shift work, and CKD risk persisted when applying an alternative PRS approach described by Yu et al. (26) (Supplementary Figures S7–S11). Using this alternative PRS, the overall patterns of joint associations on an additive scale remained consistent (Supplementary Tables S37–S41). Notably, while the super-additive association between high genetic risk and permanent night shift work remained significant (RERI: 0.28, 95% CI: 0.07–0.88), some specific estimates showed sensitivity to the PRS calculation method. For instance, in the intermediate genetic risk group, the joint association for >10 night shifts/month shifted from a super-additive pattern in the primary analysis to a significant sub-additive association in the secondary analysis (RERI: −0.42, 95% CI: −1.03 to −0.03). This indicates that while the core findings are stable, certain subgroup estimates may be influenced by the genetic risk stratification approach. When the association between night shift work and incident CKD was reassessed using competing risk models accounting for mortality, the conclusions remained consistent (Supplementary Figure S12). Similarly, Findings remained robust in sensitivity analyses addressing temporal clarity. After excluding participants who started night shifts post-recruitment and truncating exposure durations at baseline, significant associations with incident CKD persisted across all metrics. These results confirm that observed risks are driven by established pre-follow-up occupational history rather than immortal time bias (Supplementary Tables S42–S45).

## Discussion

In this large-scale study, night shift work was positively associated with an increased risk of incident CKD. Specifically, permanent night shift work exhibited a significant hazard (HR 1.19, 95% CI 1.06–1.34), representing an absolute risk increase of 0.91 cases per 1,000 person-years. Furthermore, the 10-year cumulative risk reached its highest levels among those with high-frequency (3.26%), consecutive (3.55%), or short-duration (<8 h) shifts (4.02%). These associations were particularly notable among individuals with greater genetic susceptibility, suggesting a compounding effect on inherent CKD risk.

In spite of mounting evidence connecting night shift work to diverse health risks, research on its association with the incidence of CKD remains scarce. Earlier research has demonstrated that shift workers, independent of exposure to nephrotoxic agents like halogenated hydrocarbons, commonly exhibit increased urinary albumin levels (27). Additionally, another study showed that in police officers, eGFR decline was more pronounced with increased night shift work hours (28). In our study, we found that irregular and permanent shift work are associated with a 6 and 19% higher risk of CKD, respectively. Disruption of the synchronization between biological rhythms and the sleep-wake cycle may constitute a potential pathological mechanism. Shift and night work disrupt energy expenditure patterns and the secretion rhythms of related hormones (such as leptin, ghrelin, thyroid-stimulating hormone, insulin, and melatonin), affecting the sleep-wake cycle and circadian rhythms, thereby interfering with normal metabolic functions (29). The kidney, which has a circadian rhythm, controls processes like the renin-angiotensin-aldosterone system (RAAS) (30). Shift work disrupts this rhythm, potentially leading to excessive RAAS activation, resulting in overproduction of angiotensin II, which causes renal vasoconstriction and increased glomerular pressure. Additionally, increased aldosterone activity damages endothelial cells, causing increased protein leakage that progresses to proteinuria and diminished glomerular filtration rate, ultimately raising CKD risk (31). Animal models of circadian regulatory genes, such as casein kinase-1 epsilon, show that short-cycle mutations in the tau gene lead to severe kidney diseases, including proteinuria, renal tubular dilation, and cell apoptosis. However, light cycles adapted to this genotype (22 h) can normalize behavioral patterns and restore normal kidney structure and function (32).

Moreover, our new results derived from comprehensive lifetime employment data emphasize the joint impact of both the duration and frequency of night shift work. A previous study involving 15,775 participants found that retired individuals with a history of shift work had an elevated risk of CKD, with those working night shifts for either <10 years (64% higher risk) or >20 years (48% higher risk) showing particularly increased risk compared to non-shift workers (33). However, our study observed a non-monotonic association, where individuals with <14 years of night shift work had a higher risk of CKD. This lack of a strictly monotonic association trend may be related to the “healthy worker effect,” where, with increasing age or declining health, shift workers tend to transition to less strenuous work (34). Additionally, long-term night shift work may lead workers to fully adjust their melatonin rhythms to adapt to nighttime work, which could explain why individuals with >14 years of night shift work did not show a higher CKD risk. Our study also found that night shifts longer than 12 h did not significantly increase the risk of CKD. A potential explanation is that workers with longer night shifts may have more opportunities for short breaks during work, which could help mitigate the negative effects of the job. However, existing evidence suggests that prolonged fixed night shift schedules fail to adequately realign circadian rhythms in the majority of individuals (35). Moreover, in further analysis of night shift frequency, we found that individuals with high-frequency night shifts per month had a 23% increased risk of CKD, while those with high-frequency consecutive night shifts faced an even higher risk of 31%, further supporting this notion. It is worth noting that compared to other populations, long-term individuals working night shifts show healthier lifestyle patterns, such as lower smoking and drinking habits and a higher proportion of manual labor work (indicating higher physical activity), which may support the findings of this study.

In addition to circadian disruption, sleep disturbances may partly clarify the relationship between shift work and CKD. Research has indicated that inadequate sleep duration and impaired sleep quality are linked to reduced renal function (36–39). Compared with day shift workers, irregular and permanent night shift workers had 2.27 and 3.82% less sleep, respectively, indirectly supporting this view. Sleep deprivation may increase sympathetic activity, disrupt blood pressure and metabolism, and cause oxidative stress, ultimately impairing glomerular function and lowering eGFR (36, 40–44). The link between shift work and CKD also appears to differ by sex. Female participants with a history of night shift work exhibited an increased risk of CKD, while no such association was observed in men, consistent with findings from a KNHANES-based study (45). This may be related to greater circadian sensitivity in women, leading to poorer sleep quality when rhythms are disrupted (46–48).

The stratified analyses identified differential susceptibility patterns, with metabolically healthy (normotensive/non-diabetic, *P*_interaction_ <0.03) and cardiovascular disease-free White populations (*P*_interaction_ <0.001) showing particularly strong night shift-associated CKD risk. These differential effects may be attributed to: (1) potential renal protective mechanisms (e.g., endothelial function improvement, oxidative stress reduction) from commonly prescribed metabolic medications (e.g., ACE inhibitors, statins) in patients with hypertension, diabetes or cardiovascular disease (49–51); and (2) limited statistical power in non-White subgroups due to sample size constraints (White participants >97%). These results highlight the need to account for baseline metabolic status and ethnic differences when evaluating night shift-related CKD risk.

To our knowledge, this represents the first comprehensive analysis to assess the combined impact of shift work and genetic risk on CKD development. The results indicate a joint effect between genetic susceptibility and current shift work (all *P*_interaction_ <0.001), indicating that extended duration, greater frequency of night shifts, and higher genetic risk synergistically elevate CKD risk. Notably, our additive analysis of joint associations on an additive scale further revealed heterogeneous interaction patterns across different genetic risk and exposure strata. Significant super-additive associations were specifically identified in high-genetic-risk individuals under high-intensity shift patterns, most notably for permanent night work. In contrast, sub-additive associations were observed in other contexts, such as the joint association between intermediate genetic risk and specific shift durations (e.g., 2–14 years). These patterns remained largely consistent across our primary and sensitivity analyses, suggesting that the combined impact of genetic susceptibility and circadian disruption on CKD risk is complex and depends on the specific nature of the shift work exposure. Although some interaction was observed in the associations between current or lifetime night shift exposure and CKD across models, all analyses consistently demonstrated significant positive trends in hazard ratios. These results highlight the importance of targeting individuals with elevated genetic risk for CKD when considering shift work interventions, particularly through minimizing night shift exposure to mitigate disease risk.

This study leverages prospective cohort data from over 250,000 participants in the UK Biobank, with standardized occupational exposure assessments and sensitivity analyses, suggesting that optimizing shift schedules, particularly for high genetic risk groups, may hold potential preventive value for reducing CKD risk. However, this study has several limitations. First, due to the observational nature of the study, causal inferences between shift work and CKD cannot be drawn. Second, although CKD events were defined based on ICD codes and follow-up creatinine and urine protein data to reduce misclassification, the limited availability of serial UACR measurements during follow-up—with only a minority of participants undergoing repeat testing—may have resulted in the under-ascertainment of early-stage CKD manifested exclusively as new-onset albuminuria. Third, demographic data were self-reported, potentially introducing recall bias. Fourth, employment information was only collected at baseline, and changes during follow-up were not captured. Additionally, since the study population is primarily of European ancestry, the generalizability of the findings to other ethnic groups is limited. Finally, owing to selection bias favoring healthy volunteers, the lifestyle profiles of UK Biobank participants may not accurately reflect those of the general population, warranting caution when generalizing these results (52).

## Conclusions

These findings suggest that night shift work, especially permanent schedules and adverse shift patterns, may contribute to a higher risk of CKD, particularly among individuals with high genetic susceptibility. Whether adjusting night shift work duration and frequency can effectively reduce CKD risk requires further investigation, which holds important implications for the future optimization of shift work schedules.

## Data Availability

Publicly available datasets were analyzed in this study. This data can be found here: UK Biobank Consortium website (https://www.ukbiobank.ac.uk/).
