# Supplementary material for: Association between night shift work, genetic risk, and chronic kidney disease: a prospective cohort study based on the UK Biobank
Source: Front Public Health. 2026 Apr 30;14:1772746. doi: 10.3389/fpubh.2026.1772746 (PMC13171536; doi:10.3389/fpubh.2026.1772746)
Supplement: Supplementary file 1 [file Data_Sheet_1.pdf]

Supplemental Materials

Supplementary Table 1. UK Biobank shift work classification Reference Table Based on Occupational Survey Questionnaire Data

Supplementary Table 2. Definitions and Codes for Identifying Incident CKD

Supplementary Table 3. Definitions and Codes for Associated Comorbidities

Supplementary Figure 1. Penalized spline Cox model of lifelong night shift work and CKD risk

Supplementary Table 4. Number and percentage of missing data for covariates in the study population

Supplementary Figure 2. Kaplan-Meier survival curves for incident CKD according to category of Current night shift work

Supplementary Table 5. Baseline characteristics of the study participants according to categories of Period spent working night shifts(n=67097)

Supplementary Table 6. Baseline characteristics of the study participants according to categories of Number of night shifts worked monthly(n=67097)

Supplementary Table 7. Baseline characteristics of the study participants according to categories of Usual length of each night shift (n=67097)

Supplementary Table 8. Baseline characteristics of the study participants according to categories of Consecutive night shifts(n=67097)

Supplementary Table 9. Mediation Analysis of covariate in night shift-CKD association

Supplementary Table 10. Risk of incident CKD according to different genetic risk based on SNPs from Wuttke et al. (In the current night shift exposure cohort)

Supplementary Table 11. Risk of incident CKD according to different genetic risk based on SNPs from Wuttke et al. (In the lifetime night shift exposure cohort)

Supplementary Figure 3. Risk of incident CKD according to Period spent working night shifts and genetic risk

Supplementary Figure 4. Risk of incident CKD according to Number of night shifts worked monthly and genetic risk

Supplementary Figure 5. Risk of incident CKD according to Usual length of each night shift and genetic risk

Supplementary Figure 6. Risk of incident CKD according to Consecutive night shifts and genetic risk

Supplementary Table 12 . Joint association of PRS and Current employment status with CKD risk on an additive scale

Supplementary Table 13 . Joint association of PRS and Period spent working with CKD risk on an additive scaleCKD risk

Supplementary Table 14 . Joint association of PRS and Number of night shifts worked monthly with CKD risk on an additive scale

Supplementary Table 15 . Joint association of PRS and Usual length of each night shift with CKD risk on an additive scale

Supplementary Table 16 . Joint association of PRS and Consecutive night shifts with CKD risk on an additive scale

Supplementary Table 17. Baseline characteristics according to categories of Current night shift work without covariate imputation(n=218246)

Supplementary Table 18. Baseline characteristics of the study participants according to categories of

Period spent working night shifts without covariate imputation(n=61567)

Supplementary Table 19. Baseline characteristics of the study participants according to categories of Number of night shifts worked monthly without covariate imputation(n=61567)

Supplementary Table 20. Baseline characteristics of the study participants according to categories of Usual length of each night shift without covariate imputation(n=61567)

Supplementary Table 21. Baseline characteristics of the study participants according to categories of Consecutive night shifts without covariate imputation(n=61567)

Supplementary Table 22. Associations between the Current night shift work and risks of CKD without covariate imputation(n=218246)

Supplementary Table 23. Associations between the Period spent working night shifts and risks of CKD without covariate imputation(n=61567)

Supplementary Table 24. Associations between the Number of night shifts worked monthly and risks of CKD without covariate imputation(n=61567)

Supplementary Table 25. Associations between the Usual length of each night shift and risks of CKD without covariate imputation(n=61567)

Supplementary Table 26. Associations between the Consecutive night shifts and risks of CKD without covariate imputation(n=61567)

Supplementary Table 27. Association of Current night shift work with CKD Risk After Excluding CKD Cases Within the First Two Years(n=250781)

Supplementary Table 28. Association of Period spent working night shifts with CKD Risk After Excluding CKD Cases Within the First Two Years (n=67694)

Supplementary Table 29. Association of Number of night shifts worked monthly with CKD Risk After Excluding CKD Cases Within the First Two Years (n=67694)

Supplementary Table30 . Association of Usual length of each night shift with CKD Risk After Excluding CKD Cases Within the First Two Years (n=67694)

Supplementary Table31 . Association of Consecutive night shifts with CKD Risk After Excluding CKD Cases Within the First Two Years (n=67694)

Supplementary Table 32. Stratified Analysis of Current night shift work and CKD Risk by covariates

Supplementary Table 33. Stratified Analysis of Period spent working night shifts and CKD Risk by covariates

Supplementary Table 34. Stratified Analysis of Number of night shifts worked monthly and CKD Risk by covariates

Supplementary Table 35. Stratified Analysis of Usual length of each night shift and CKD Risk by covariates

Supplementary Table 36. Stratified Analysis of Consecutive night shifts and CKD Risk by covariates

Supplementary Figure 7. Stratified Analysis of Current night shift work and CKD Risk by PRS Group base on the SNPs from Yu et al.

Supplementary Figure 8. Risk of incident CKD according to Period spent working night shifts and genetic risk base on the SNPs from Yu et al.

Supplementary Figure 9. Stratified Analysis of Number of night shifts worked monthly and CKD Risk by PRS Group base on the SNPs from Yu et al.

Supplementary Figure 10. Stratified Analysis of Usual length of each night shift and CKD Risk by PRS Group base on the SNPs from Yu et al.
Supplementary Figure 11. Stratified Analysis of Consecutive night shifts and CKD Risk by PRS Group base on the SNPs from Yu et al.
Supplementary Table 37 . Sensitivity analysis of the joint association between PRS and Current employment status on CKD risk: an additive-scale analysis
Supplementary Table 38 . Sensitivity analysis of the joint association between PRS and Period spent working night shifts on CKD risk: an additive-scale analysis
Supplementary Table 39 . Sensitivity analysis of the joint association between PRS and Number of night shifts worked monthly on CKD risk: an additive-scale analysis
Supplementary Table 40 . Sensitivity analysis of the joint association between PRS and Usual length of each night shift on CKD risk: an additive-scale analysis
Supplementary Table 41 . Sensitivity analysis of the joint association between PRS and Consecutive night shifts on CKD risk: an additive-scale analysis
Supplementary Figure 12. Associations between the Current or lifelong employment status and risks of CKD using competing risk models.
Supplementary Table 42. Associations between the pre-baseline Period spent working night shifts and risks of CKD (n=67881)
Supplementary Table 43. Associations between the pre-baseline Number of night shifts worked monthly and risks of CKD (n=67881)
Supplementary Table 44. Associations between the pre-baseline Usual length of each night shift and risks of CKD (n=67881)
Supplementary Table 45. Associations between the pre-baseline Consecutive night shifts and risks of CKD (n=67881)

Supplementary Table 1.UK Biobank shift work classification Reference Table Based on Occupational Survey Questionnaire Data

**Ouestion 1:Does your work involve shift work?**	**Question 2: Does your job involve night shifts?**	**Shift work type**
Never/rarely		Never/rarely night shifts(reference category)
Sometimes	Never/rarely	Never/rarely night shifts
	Sometimes	Irregular night shift
	Usually	Irregular night shift
	Always	Irregular night shift
Usually	Never/rarely	Never/rarely night shifts
	Sometimes	Irregular night shift
	Usually	Irregular night shift
	Always	Irregular night shift
Always	Never/rarely	Never/rarely night shifts
	Sometimes	Irregular night shift
	Usually	Irregular night shift
	Always	Permanent night shift

Reference Group (Never/rarely night shifts): Participants who answered Never/rarely to Question 2, or Never/rarely to Question 1 (regardless of their response to Question 2);

Irregular Night Shifts: Those who reported Sometimes/Usually shift work (Question 1) and Sometimes/Usually/Always night shifts (Question 2), or Always shift work but not Always night shifts;

Permanent Night Shifts: Only participants who answered Always to both Questions 1 and 2.

Supplementary Table 2. Definitions and Codes for Identifying Incident CKD

Outcome	Codes		
	**ICD-10**	**ICD-9**	**OPCS-4**
Chronic kidney disease	D59.3, E10.2, E11.2, E12.2, E13.2, E14.2, E85.3, I12, I13, I15.0, M10.3, N02, N03, N04, N05, N06, N07, N08, N11, N12, N13, N14, N15, N16, N18, N19, N25, N26, N28.0, N28.8, N28.9, N39.1, O10.2, O11, O12.1, O12.2, O14, Q60, R39.2, T86.1, Z49.0, Z94.0	403, 581, 582, 583, 585, 586, 587, 588, 589, 591, 642, 2503, 7530, 7531, 7532, 7533, 7910	L74.1-74.6, L74.8-74.9, M01.2-01.9, M02.3, M08.4, M17.2, M17.4, M17.8-17.9, X40.2, X40.5-40.6, X41.1-41.2

Abbreviations: ICD-10, International Classification of Diseases-10th Revision; ICD-9,International Classification of Diseases-9th Revision; OPCS-4, Office of Population Censuses and Surveys Classification of Interventions and Procedures.

Supplementary Table 3. Definitions and Codes for Associated Comorbidities

Comorbidities	Codes		
	**ICD-10**	**ICD-9**	**OPCS-4**
Cardiovascular disease	I00-I99 ， Z95.1 Z95.5	390-459	K40–K46, K49, K50, K75,A05.2–A05.4, L34.3, L35.1, L35.3,K62.1–K62.3
Hypertension	ICD-10:I11-I13,I15,O10	401-405	-
Diabetes	E10–E14	250	-
Cancer	C00–C97, Z85	140–209, V10	-

Abbreviations: ICD-10, International Classification of Diseases-10th Revision; ICD-9, International Classification of Diseases-9th Revision; OPCS-4, Office of Population Censuses and Surveys Classification of Interventions and Procedure.

Supplementary Figure 1. Penalized spline Cox model of lifelong night shift work and CKD risk

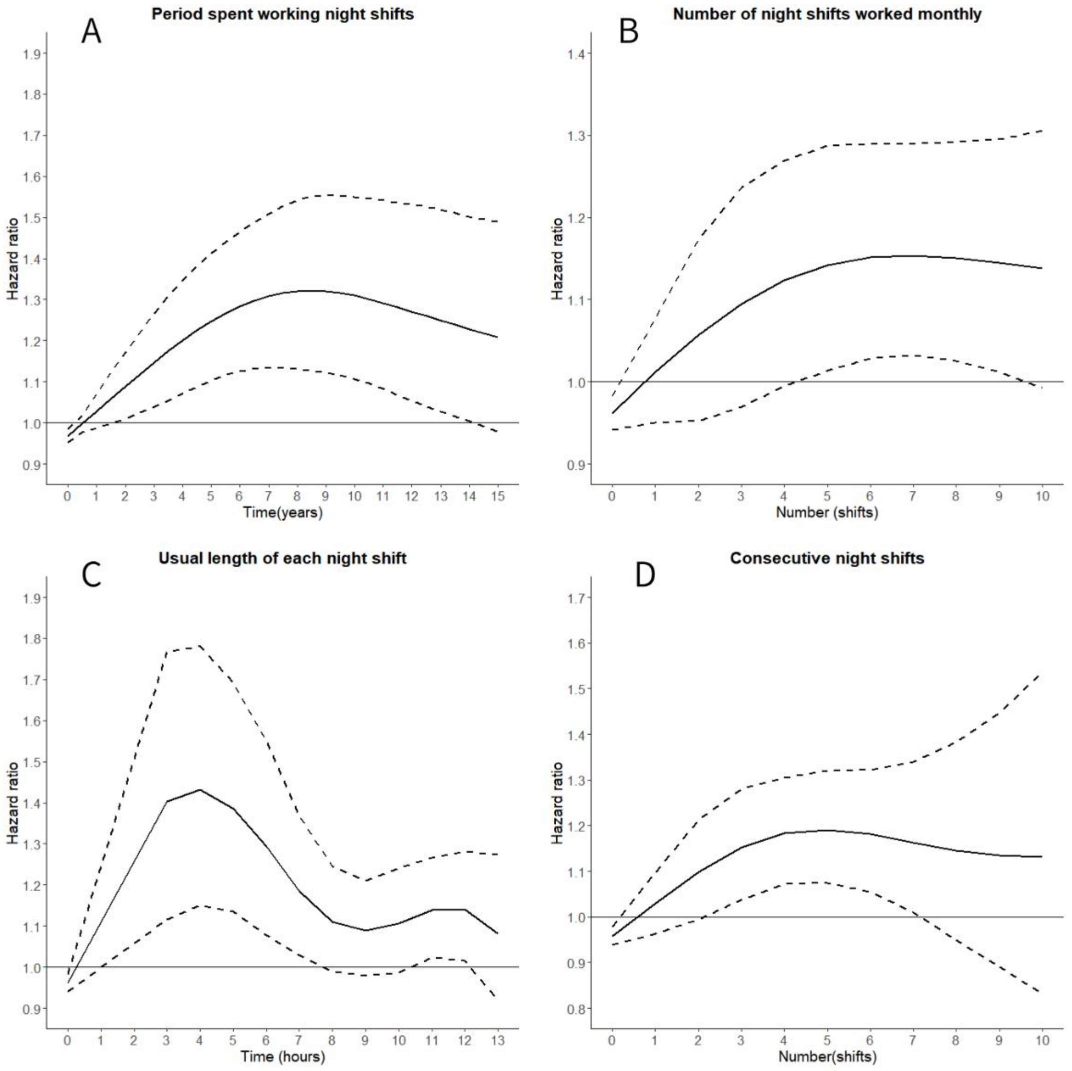

(A)Assessing CKD risk by Period spent working night shifts using penalized splines;(B)Assessing CKD risk by Number of night shifts worked monthly using penalized splines;(C)Assessing CKD risk by Usual length of each night shift using penalized splines;(D)Assessing CKD risk by Consecutive night shifts using penalized splines.Cox models with penalized splines were adjusted for gender, age, race, BMI, alcohol, smoking status, education, the Townsend deprivation index, healthy diet score, labor work level, and baseline diseases (including hypertension, diabetes, CVD, and cancer).

Supplementary Table 4. Number and percentage of missing data for covariates in the study population

Covariates	Missing (n)	Missing (%)
Ethnicity	757	0.30%
Education	28070	11.12%
Townsend Deprivation Index	353	0.14%
Smoking status	682	0.27%
Alcohol consumption	227	0.09%
Healthy diet score	8557	3.39%
Sleep duration	833	0.33%
Labor work	303	0.12%
BMI	959	0.38%

Abbreviation: BMI, body mass index.

Supplementary Figure 2. Kaplan-Meier survival curves for incident CKD according to category of Current night shift work

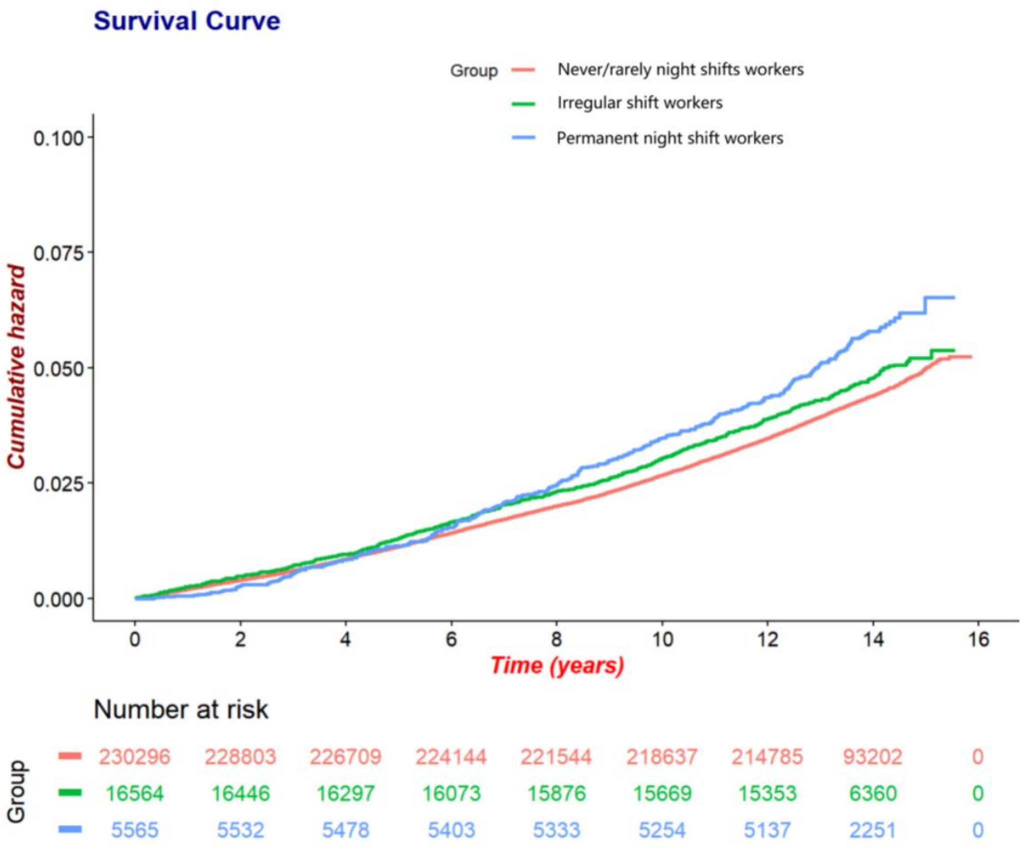

Cox models were adjusted for gender, age, race, BMI, alcohol, smoking status, education, the Townsend deprivation index, healthy diet score, labor work level, and baseline diseases (including hypertension, diabetes, CVD, and cancer).

Supplementary Table 5. Baseline characteristics of the study participants according to categories of Period spent working night shifts(n=67097)

Characteristics	Total	Period spent working night shifts			
		None	<2years	2-14years	>14years
No. participants	67907	51398	6148	8448	1913
Age(years)±SD	52.8±6.8	53.0±6.8	51.9±7.0	52.5±6.8	52.8±6.5
sex(n,%)					
Female	37151(54.7)	29785(57.9)	2583(42.0)	3787(44.8)	996(52.1)
Male	30756(45.3)	21613(42.1)	3565(58.0)	4661(55.2)	917(47.9)
Ethnic(n,%)					
White	66011(97.2)	50113(97.5)	5875(95.6)	8161(96.6)	1862(97.3)
Asian	721(1.1)	516(1.0)	93(1.5)	106(1.3)	6(0.3)
Black	439(0.6)	269(0.5)	78(1.3)	66(0.8)	26(1.4)
Other	736(1.1)	500(1.0)	102(1.7)	115(1.4)	19(1.0)
Education(n, %)					
College or University degree	35882(52.8)	29002(56.4)	2762(44.9)	3338(39.5)	780(40.8)
Other	32025(47.2)	22396(43.6)	3386(55.1)	5110(60.5)	1133(59.2)
Townsend deprivation index	-2.4(-3.8, -0.1)	-2.43(-3.8, -0.2)	-2.1(-3.6,0.4)	-2.2(-3.7, 0.2)	-2.6(-3.9, -0.6)
BMI(n,%, kg/m2)					
Mean	26.0(23.5, 28.9)	25.7(23.4, 28.7)	26.7(24.2,30.0)	26.8(24.1, 29.8)	26.7(24.0, 29.5)
<18.5	352(0.5)	302(0.6)	9(0.1)	29(0.3)	12(0.6)
18.5–24.9	26963(39.7)	21531(41.9)	2005(32.6)	2783(32.9)	644(33.7)
25–29.9	27768(40.9)	20703(40.3)	2611(42.5)	3609(42.7)	845(44.2)
≥30	12824(18.9)	8862(17.2)	1523(24.8)	2027(24.0)	412(21.5)
Smoking(n,%)					
Never	41023(60.4)	32032(62.3)	3328(54.1)	4565(54.0)	1098(57.4)
Previous	21864(32.2)	15904(30.9)	2200(35.8)	3087(36.5)	673(35.2)
Current	5020(7.4)	3462(6.7)	620(10.1)	796(9.4)	142(7.4)
Alcohol(n,%)					
Never	1605(2.4)	1234(2.4)	143(2.3)	184(2.2)	44(2.3)
Previous	1579(2.3)	1135(2.2)	138(2.2)	241(2.9)	65(3.4)
Current	64723(95.3)	49029(95.4)	5867(95.4)	8023(95.0)	1804(94.3)
Sleep duration(hours)	7.0(7.0, 8.0)	7.0(7.0, 8.0)	7.0(6.0, 8.0)	7.0(6.0, 8.0)	7.0(6.0, 8.0)
Healthy diet score±SD	2.9±1.3	2.9±1.3	2.8±1.3	2.8±1.3	2.8±1.3
Labor work(n,%)					
Never	51339(75.6)	41311(80.4)	3983(64.8)	4979(58.9)	1066(55.7)
Sometime	11608(17.1)	7287(14.2)	1426(23.2)	2321(27.5)	574(30.0)
Usually	2882(4.2)	1617(3.1)	406(6.6)	693(8.2)	166(8.7)
Always	2078(3.1)	1183(2.3)	333(5.4)	455(5.4)	107(5.6)
cancer(n,%)	5543(8.2)	4298(8.4)	421(6.8)	664(7.9)	160(8.4)
Hypertension(n,%)	13383(19.7)	9871(19.2)	1256(20.4)	1786(21.1)	470(24.6)

Characteristics	Total	Period spent working night shifts			
		None	<2years	2-14years	>14years
Diabetes(n,%)	1831(2.7)	1231(2.4)	226(3.7)	290(3.4)	84(4.4)
Cardiovascular disease(n,%)	2199(3.2)	1529(3.0)	278(4.5)	327(3.9)	65(3.4)
eGFR(mL/min per 1.73 m2)	95.6(86.4, 102.4)	95.5(86.3,102.3)	96.2(86.9,103.0)	95.9(86.7, 102.6)	95.6(86.7, 102.4)

Data were presented as frequency (%), mean ± standard deviation or median (interquartile range). Abbreviation: BMI, body mass index. eGFR, estimated Glomerular Filtration Rate； Data are mean ± SD or median (interquartile range) or frequencies (percentages).

Supplementary Table 6. Baseline characteristics of the study participants according to categories of Number of night shifts worked monthly (n=67097)

Characteristics	Total	Number of night shifts worked monthly			
		None	<5/month	5-10/month	>10/month
No. participants	67907	51398	4542	7567	4400
Age(years)±SD	52.8±6.8	53.0±6.8	52.3±6.8	52.1±6.7	52.8±6.9
sex(n,%)					
Female	37151(54.7)	29785(57.9)	2216(48.8)	3267(43.2)	1883(42.8)
Male	30756(45.3)	21613(42.1)	2326(51.2)	4300(56.8)	2497(57.2)
Ethnic(n,%)					
White	66011(97.2)	50113(97.5)	4338(95.5)	7329(96.9)	4231(96.2)
Asian	721(1.1)	516(1.0)	68(1.5)	81(1.1)	56(1.3)
Black	439(0.6)	269(0.5)	55(1.2)	62(0.8)	53(1.2)
Other	736(1.1)	500(1.0)	81(1.8)	95(1.3)	60(1.4)
Education(n, %)					
College or University degree	35882(52.8)	29002(56.4)	2080(45.8)	3167(41.9)	1633(37.1)
Other	32025(47.2)	22396(43.6)	2462(54.2)	4400(58.1)	2767(62.9)
Townsend deprivation index	-2.4(-3.8, -0.1)	-2.4(-3.8, -0.2)	-2.3(-3.7,0.0)	-2.3(-3.8, -0.0)	-1.98(-3.6, 0.7)
BMI(n,%, kg/m2)					
Mean	26.0(23.5, 28.9)	25.7(23.,4 28.7)	26.4(23.9, 29.5)	26.7(24.1, 29.8)	27.1(24.4, 30.3)
<18.5	352(0.5)	302(0.6)	15(0.3)	22(0.3)	13(0.3)
18.5–24.9	26963(39.7)	21531(41.9)	1599(35.2)	2509(33.2)	1324(30.1)
25–29.9	27768(40.9)	20703(40.3)	1933(42.6)	3249(42.9)	1883(42.8)
≥30	12824(18.9)	8862(17.2)	995(21.9)	1787(23.6)	1180(26.8)
Smoking(n,%)					
Never	41023(60.4)	32032(62.3)	2493(55.3)	4260(56.3)	2218(50.4)
Previous	21864(32.2)	15904(30.9)	1632(35.9)	2657(35.1)	1671(38.0)
Current	5020(7.4)	3462(6.7)	397(8.7)	650(8.6)	511(11.6)
Alcohol(n,%)					
Never	1605(2.4)	1234(2.4)	120(2.6)	151(2.0)	100(2.3)
Previous	1579(2.3)	1135(2.2)	125(2.8)	190(2.5)	129(2.9)
Current	64723(95.3)	49029(95.4)	4297(94.6)	7226(95.5)	4171(94.8)
Sleep duration(hours)	7.0(7.0, 8.0)	7.0(7.0, 8.0)	7.0(7.0, 8.0)	7.0(6.0, 8.0)	7.0(6.0, 8.0)
Healthy diet score±SD	2.9±1.3	2.9±1.3	2.8±1.3	2.8±1.3	2.8±1.3
Labor work(n,%)					
Never	51339(75.6)	41311(80.4)	2842(62.6)	4780(63.2)	2406(54.7)
Sometime	11608(17.1)	7287(14.2)	1165(25.6)	1912(25.3)	1244(28.3)
Usually	2882(4.2)	1617(3.1)	332(7.3)	522(6.9)	411(9.3)
Always	2078(3.1)	1183(2.3)	203(4.5)	353(4.7)	339(7.7)
Cancer(n,%)	5543(8.2)	4298(8.4)	359(7.9)	543(7.2)	343(7.8)
Hypertension(n,%)	13383(19.7)	9871(19.2)	947(20.8)	1562(20.6)	1003(22.8)

Characteristics	Total	Number of night shifts worked monthly			
		None	<5/month	5-10/month	>10/month
Diabetes(n,%)	1831(2.7)	1231(2.4)	154(3.4)	269(3.6)	177(4.0)
Cardiovascular disease(n,%)	2199(3.2)	1529(3.0)	155(3.4)	307(4.1)	208(4.7)
eGFR(mL/min per 1.73 m2)	95.6(86.4, 102.4)	95.52(86.3,102.3)	96.3(87.3,102.9)	95.9(86.6, 102.8)	95.7(86.5,102.6)

Data were presented as frequency (%), mean ± standard deviation or median (interquartile range). Abbreviation: BMI, body mass index. eGFR, estimated Glomerular Filtration Rate； Data are mean ± SD or median (interquartile range) or frequencies (percentages).

Supplementary Table 7. Baseline characteristics of the study participants according to categories of Number of night shifts worked monthly (n=67097)

Characteristics	Total	Usual length of each night shift			
		None	<8h	8-12h	>12h
No. participants	67907	51398	1825	13492	1192
Age(years)±SD	52.8±6.8	53.0±6.8	52.8±7.1	52.4±6.8	51.4±6.4
sex(n,%)					
Female	37151(54.7)	29785(57.9)	728(39.9)	6047(44.8)	591(49.6)
Male	30756(45.3)	21613(42.1)	1097(60.1)	7445(55.2)	601(50.4)
Ethnic(n,%)					
White	66011(97.2)	50113(97.5)	1752(96.0)	13003(96.4)	1143(95.9)
Asian	721(1.1)	516(1.0)	20(1.1)	163(1.2)	22(1.8)
Black	439(0.6)	269(0.5)	21(1.2)	142(1.1)	7(0.6)
Other	736(1.1)	500(1.0)	32(1.8)	184(1.4)	20(1.7)
Education(n, %)					
College or University degree	35882(52.8)	29002(56.4)	877(48.1)	5230(38.8)	773(64.8)
Other	32025(47.2)	22396(43.6)	948(51.9)	8262(61.2)	419(35.2)
Townsend deprivation index	-2.4(-3.8, -0.1)	-2.4(-3.8, -0.2)	-1.9(-3.6, 0.9)	-2.2(-3.7, 0.1)	-2.4(-3.8, -0.2)
BMI(n,%, kg/m2)					
Mean	26.0(23.5, 28.9)	25.7(23.4, 28.7)	26.6(24.1, 29.8)	26.8(24.2, 29.9)	25.9(23.4, 29.0)
<18.5	352(0.5)	302(0.6)	6(0.3)	36(0.3)	8(0.7)
18.5–24.9	26963(39.7)	21531(41.9)	600(32.9)	4346(32.2)	486(40.8)
25–29.9	27768(40.9)	20703(40.3)	792(43.4)	5819(43.1)	454(38.1)
≥30	12824(18.9)	8862(17.2)	427(23.4)	3291(24.4)	244(20.5)
Smoking(n,%)					
Never	41023(60.4)	32032(62.3)	929(50.9)	7300(54.1)	762(63.9)
Previous	21864(32.2)	15904(30.9)	728(39.9)	4885(36.2)	347(29.1)
Current	5020(7.4)	3462(6.7)	168(9.2)	1307(9.7)	83(7.0)
Alcohol(n,%)					
Never	1605(2.4)	1234(2.4)	34(1.9)	305(2.3)	32(2.7)
Previous	1579(2.3)	1135(2.2)	46(2.5)	374(2.8)	24(2.0)
Current	64723(95.3)	49029(95.4)	1745(95.6)	12813(95.0)	1136(95.3)
Sleep duration(hours)	7.0(7.0, 8.0)	7.00(7.0, 8.0)	7.00(6.0, 8.0)	7.0(6.0, 8.0)	7.0(7.0, 8.0)
Healthy diet score±SD	2.9±1.3	2.9±1.3	2.9±1.3	2.8±1.3	2.9±1.3
Labor work(n,%)					
Never	51339(75.6)	41311(80.4)	1202(65.9)	7979(59.1)	847(71.1)
Sometime	11608(17.1)	7287(14.2)	433(23.7)	3649(27.0)	239(20.1)
Usually	2882(4.2)	1617(3.1)	95(5.2)	1101(8.2)	69(5.8)
Always	2078(3.1)	1183(2.3)	95(5.2)	763(5.7)	37(3.1)
Cancer(n,%)	5543(8.2)	4298(8.4)	147(8.1)	1004(7.4)	94(7.9)

Characteristics	Total	Usual length of each night shift			
		None	<8h	8-12h	>12h
Hypertension(n,%)	13383(19.7)	9871(19.2)	408(22.4)	2908(21.6)	196(16.4)
Diabetes(n,%)	1831(2.7)	1231(2.4)	78(4.3)	487(3.6)	35(2.9)
Cardiovascular disease(n,%)	2199(3.2)	1529(3.0)	72(3.9)	564(4.2)	34(2.9)
eGFR(mL/min per 1.73 m2)	95.6(86.4, 102.4)	95.52(86.3,102.3)	95.99(87.4,102.5)	95.8(86.1,102.8)	96.9(87.6, 103.1)

Data were presented as frequency (%), mean ± standard deviation or median (interquartile range). Abbreviation: BMI, body mass index. eGFR, estimated Glomerular Filtration Rate； Data are mean ± SD or median (interquartile range) or frequencies (percentages).

Supplementary Table 8. Baseline characteristics of the study participants according to categories of Consecutive night shifts(n=67097)

Characteristics	Total	Consecutive night shifts			
		None	2 shifts/month	2-7shifts/month	>7shifts/month
No. participants	67907	51398	2751	12144	1614
Age(years)±SD	52.8±6.8	53.0±6.8	52.4±6.9	52.2±6.8	53.5±6.9
sex(n,%)					
Female	37151(54.7)	29785(57.9)	1363(49.5)	5308(43.7)	695(43.1)
Male	30756(45.3)	21613(42.1)	1388(50.5)	6836(56.3)	919(56.9)
Ethnic(n,%)					
White	66011(97.2)	50113(97.5)	2652(96.4)	11697(96.3)	1549(96.0)
Asian	721(1.1)	516(1.0)	35(1.3)	141(1.2)	29(1.8)
Black	439(0.6)	269(0.5)	24(0.9)	131(1.1)	15(0.9)
Other	736(1.1)	500(1.0)	40(1.5)	175(1.4)	21(1.3)
Education(n, %)					
College or University degree	35882(52.8)	29002(56.4)	1518(55.2)	4695(38.7)	667(41.3)
Other	32025(47.2)	22396(43.6)	1233(44.8)	7449(61.3)	947(58.7)
Townsend deprivation index	-2.4(-3.8, -0.1)	-2.4(-3.8, -0.2)	-2.2(-3.7, 0.4)	-2.2(-3.7, 0.2)	-2.2(-3.7, 0.2)
BMI(n,%, kg/m2)					
Mean	26.0(23.5, 28.9)	25.7(23.4, 28.7)	26.1(23.5, 29.1)	26.8(24.2, 29.9)	26.9(24.4, 30.1)
<18.5	352(0.5)	302(0.6)	10(0.4)	33(0.3)	7(0.4)
18.5–24.9	26963(39.7)	21531(41.9)	1079(39.2)	3871(31.9)	482(29.9)
25–29.9	27768(40.9)	20703(40.3)	1116(40.6)	5242(43.2)	707(43.8)
≥30	12824(18.9)	8862(17.2)	546(19.8)	2998(24.7)	418(25.9)
Smoking(n,%)					
Never	41023(60.4)	32032(62.3)	1552(56.4)	6619(54.5)	820(50.8)
Previous	21864(32.2)	15904(30.9)	958(34.8)	4352(35.8)	650(40.3)
Current	5020(7.4)	3462(6.7)	241(8.8)	1173(9.7)	144(8.9)
Alcohol(n,%)					
Never	1605(2.4)	1234(2.4)	57(2.1)	280(2.3)	34(2.1)
Previous	1579(2.3)	1135(2.2)	74(2.7)	313(2.6)	57(3.5)
Current	64723(95.3)	49029(95.4)	2620(95.2)	11551(95.1)	1523(94.4)
Sleep duration(hours)	7.0(7.0, 8.0)	7.0(7.0, 8.0)	7.0(7.0, 8.0)	7.0(6.0, 8.0)	7.0(6.0, 8.0)
Healthy diet score±SD	2.9±1.3	2.9±1.3	2.9±1.3	2.8±1.3	2.8±1.3
Labor work(n,%)					
Never	51339(75.6)	41311(80.4)	1884(68.5)	7171(59.0)	973(60.3)
Sometime	11608(17.1)	7287(14.2)	586(21.3)	3296(27.1)	439(27.2)
Usually	2882(4.2)	1617(3.1)	169(6.1)	980(8.1)	116(7.2)
Always	2078(3.1)	1183(2.3)	112(4.1)	697(5.7)	86(5.3)
Cancer(n,%)	5543(8.2)	4298(8.4)	215(7.8)	905(7.5)	125(7.7)
Hypertension(n,%)	13383(19.7)	9871(19.2)	533(19.4)	2598(21.4)	381(23.6)

Characteristics	Total	Consecutive night shifts			
		None	2 shifts/month	2-7shifts/month	>7shifts/month
Diabetes(n,%)	1831(2.7)	1231(2.4)	95(3.5)	442(3.6)	63(3.9)
Cardiovascular disease(n,%)	2199(3.2)	1529(3.0)	77(2.8)	512(4.2)	81(5.0)
eGFR(mL/min per 1.73 m2)	95.6(86.4, 102.4)	95.5(86.3, 102.3)	95.9(87.1, 102.7)	96.0(86.9, 102.9)	95.6(86.0, 102.0)

Data were presented as frequency (%), mean ± standard deviation or median (interquartile range). Abbreviation: BMI, body mass index. eGFR, estimated Glomerular Filtration Rate；Data are mean ± SD or median (interquartile range) or frequencies (percentages).

Supplementary Table 9. Mediation Analysis of covariate in night shift-CKD association

Variable	ACME ($\times 10^3$)	Total Effect($\times 10^3$)	Prop. Mediated	*P* value
Chronotype	0.128	3.27	4.13%	<0.001
Townsend deprivation index	0.253	3.15	7.5%	<0.001
Sleep duration	-0.00982	3.60	0.45%	0.833
Labor work	0.391	4.10	9.61%	0.078
Healthy diet	-0.0131	3.13	0.39%	0.855
Cancer	0.00421	3.56	0.0283%	0.915
Hypertension	-0.0365	3.29	0.87%	0.667
Diabetes	0.0735	3.59	1.67%	0.489
Cardiovascular disease	-0.0294	3.37	0.95%	0.720
Smoking	0.128	3.41	3.48%	0.064
Alcohol	0.111	3.17	3.61%	0.100

ACME（Average Causal Mediation Effect),Prop. Mediated（Proportion of the total effect mediated). Cox models were adjusted for gender, age, race, BMI, alcohol, smoking status, education, the Townsend deprivation index, healthy diet score, labor work level, and baseline diseases (including hypertension, diabetes, CVD, and cancer).

Supplementary Table 10. Risk of incident CKD according to different genetic risk based on SNPs from Wuttke et al. (In the current night shift exposure cohort)

	PRS categories			
	Low genetic risk	Intermediate genetic risk	high genetic risk	
No. participants	63106	126212	63107	
No. cases (n,%)	1982 (3.14)	4971 (3.94)	3620 (5.74)	
	HR	HR (95n,% CI)	HR (95n,% CI)	[d]*P* value
[a]Model 1	1.00 (ref.)	1.27 (1.21-1.34)	1.89 (1.79-2.00)	<0.001
[b]Model 2	1.00 (ref.)	1.26 (1.20-1.33)	1.87 (1.77-1.97)	<0.001
[c]Model 3	1.00 (ref.)	1.26 (1.20-1.33)	1.88 (1.78-1.98)	<0.001

[a]Model 1:adjusted for gender, age and race;

[b]Model 2: adjusted for model 1 plus BMI, alcohol , smoking status, education, and the Townsend deprivation index;

[c]Model 3: adjusted for model 2 plus healthy diet score, labor work level, and baseline diseases (including hypertension, diabetes, CVD, and cancer).

[d]*P* values: represent the overall significance of the categorical variable, calculated using the Likelihood Ratio Test (LRT) comparing models with and without the variable.

Supplementary Table 11. Risk of incident CKD according to different genetic risk based on SNPs from Wuttke et al. (In the lifetime night shift exposure cohort)

	PRS categories			
	Low genetic risk	Intermediate genetic risk	high genetic risk	
No. participants	16977	33952	16978	
No. cases (n,%)	437 (2.57)	1106 (3.26)	853 (5.02)	
	HR	HR (95n,% CI)	HR (95n,% CI)	*P* value
[a]Model 1	1.00 (ref.)	1.30 (1.16-1.45)	2.04 (1.82-2.29)	<0.001
[b]Model 2	1.00 (ref.)	1.29 (1.16-1.45)	2.01 (1.79-2.26)	<0.001
[c]Model 3	1.00 (ref.)	1.29 (1.16-1.45)	2.02 (1.80-2.27)	<0.001

[a]Model 1:adjusted for gender, age and race;

[b]Model 2: adjusted for model 1 plus BMI, alcohol , smoking status, education, and the Townsend deprivation index;

[c]Model 3: adjusted for model 2 plus healthy diet score, labor work level, and baseline diseases (including hypertension, diabetes, CVD, and cancer).

[d]*P* values: represent the overall significance of the categorical variable, calculated using the Likelihood Ratio Test (LRT) comparing models with and without the variable.

Supplementary Figure 2. Risk of incident CKD according to Period spent working night shifts and genetic risk

Group	No.Total	No.Case	HR(95%CI)	P value
**Low genetic risk**				
None	12804	298	1.00 (ref.)	
<2 years	1498	44	1.15 (0.84 - 1.58)	0.384
2-14years	2183	83	1.50 (1.17 - 1.91)	0.001
>14 years	492	12	0.90 (0.50 - 1.60)	0.711
**Intermediate genetic risk**				
None	25776	772	1.31 (1.15 - 1.50)	< 0.001
<2 years	3041	121	1.63 (1.32 - 2.02)	< 0.001
2-14years	4183	169	1.61 (1.33 - 1.96)	< 0.001
>14 years	952	44	1.80 (1.31 - 2.48)	< 0.001
**High genetic risk**				
None	12818	622	2.15 (1.87 - 2.47)	< 0.001
<2 years	1609	90	2.31 (1.82 - 2.93)	< 0.001
2-14years	2082	117	2.28 (1.84 - 2.84)	< 0.001
>14 years	469	24	2.21 (1.46 - 3.36)	< 0.001

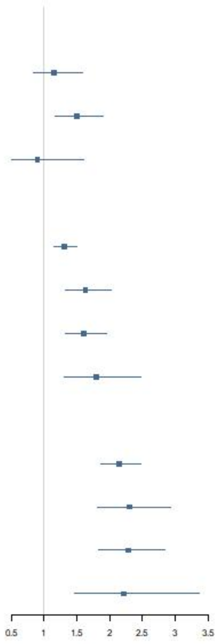

Cox models were adjusted for gender, age, race, BMI, alcohol, smoking status, education, the Townsend deprivation index, healthy diet score, labor work level, and baseline diseases (including hypertension, diabetes, CVD, and cancer).

Supplementary Figure 3. Risk of incident CKD according to Number of night shifts worked monthly and genetic risk

Group	No.Total	No.Case	HR(95%CI)	P value
**Low genetic risk**				
None	12804	298	1.00 (ref.)	
<5/month	1197	44	1.46 (1.07 - 2.01)	0.018
5-10/month	1884	64	1.40 (1.07 - 1.84)	0.015
>10/month	1092	31	0.99 (0.68 - 1.43)	0.953
**Intermediate genetic risk**				
None	25776	772	1.31 (1.15 - 1.50)	< 0.001
<5/month	2217	75	1.42 (1.10 - 1.84)	0.007
5-10/month	3734	139	1.54 (1.25 - 1.88)	< 0.001
>10/month	2225	120	2.00 (1.62 - 2.49)	< 0.001
**High genetic risk**				
None	12818	622	2.15 (1.87 - 2.47)	< 0.001
<5/month	1128	59	2.16 (1.63 - 2.86)	< 0.001
5-10/month	1949	112	2.47 (1.98 - 3.07)	< 0.001
>10/month	1083	60	2.12 (1.60 - 2.80)	< 0.001

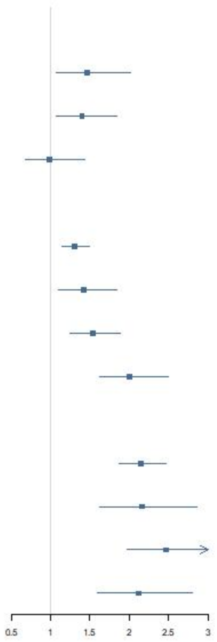

Cox models were adjusted for gender, age, race, BMI, alcohol, smoking status, education, the Townsend deprivation index, healthy diet score, labor work level, and baseline diseases (including hypertension, diabetes, CVD, and cancer).

Supplementary Figure 4. Risk of incident CKD according to Usual length of each night shift and genetic risk

Group	No.Total	No.Case	HR(95%CI)	P value
**Low genetic risk**				
None	12804	298	1.00 (ref.)	
<8h	439	22	1.82 (1.18 - 2.81)	0.007
8-12h	3423	111	1.26 (1.01 - 1.56)	0.043
>12h	311	6	0.92 (0.41 - 2.07)	0.843
**Intermediate genetic risk**				
None	25776	772	1.31 (1.15 - 1.50)	< 0.001
<8h	904	55	2.34 (1.75 - 3.13)	< 0.001
8-12h	6709	261	1.56 (1.32 - 1.85)	< 0.001
>12h	563	18	1.48 (0.92 - 2.38)	0.106
**High genetic risk**				
None	12818	622	2.15 (1.87 - 2.47)	< 0.001
<8h	482	25	2.19 (1.46 - 3.30)	< 0.001
8-12h	3360	196	2.35 (1.96 - 2.83)	< 0.001
>12h	318	10	1.56 (0.83 - 2.94)	0.165

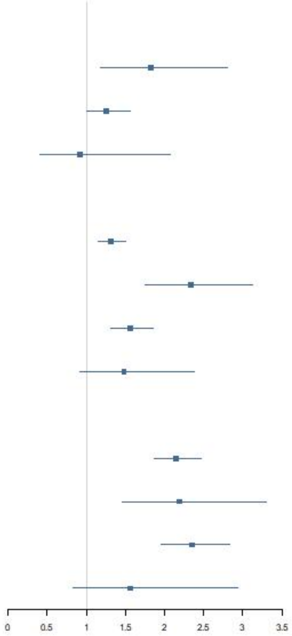

Cox models were adjusted for gender, age, race, BMI, alcohol, smoking status, education, the Townsend deprivation index, healthy diet score, labor work level, and baseline diseases (including hypertension, diabetes, CVD, and cancer).

Supplementary Figure 5. Risk of incident CKD according to Usual length of each night shift and genetic risk

Group	No.Total	No.Case	HR(95%CI)	P value
**Low genetic risk**				
None	12804	298	1.00 (ref.)	
<2shifts	688	19	1.14 (0.72 - 1.82)	0.577
2-7shifts	3044	106	1.37 (1.10 - 1.71)	0.006
>7shifts	441	14	1.08 (0.63 - 1.86)	0.768
**Intermediate genetic risk**				
None	25776	772	1.31 (1.15 - 1.50)	< 0.001
<2shifts	1326	42	1.34 (0.97 - 1.86)	0.073
2-7shifts	6074	243	1.62 (1.36 - 1.93)	< 0.001
>7shifts	776	49	2.26 (1.67 - 3.06)	< 0.001
**High genetic risk**				
None	12818	622	2.15 (1.87 - 2.47)	< 0.001
<2shifts	737	34	2.02 (1.42 - 2.89)	< 0.001
2-7shifts	3026	175	2.38 (1.97 - 2.87)	< 0.001
>7shifts	397	22	2.10 (1.36 - 3.25)	< 0.001

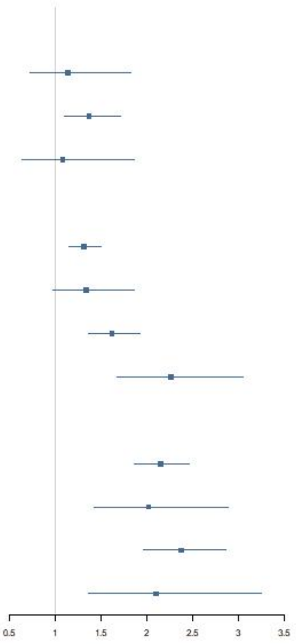

Cox models were adjusted for gender, age, race, BMI, alcohol, smoking status, education, the Townsend deprivation index, healthy diet score, labor work level, and baseline diseases (including hypertension, diabetes, CVD, and cancer).

Supplementary Table 12. Joint association of PRS and Current employment status with CKD risk on an additive scale

Group	RERI (95% CI)	AP (95% CI)	*P* value	Interaction Type
**Intermediate genetic risk**				
Irregular shift workers	-0.18 (-0.22, 0.07)	-0.14 (-0.19, 0.05)	0.152	Additive
Permanent night shift workers	-0.05 (-0.16, 0.52)	-0.04 (-0.13, 0.34)	0.611	Additive
**High genetic risk**				
Irregular shift workers	-0.08 (-0.29, -0.04)	-0.04 (-0.21, -0.03)	0.035	Sub-additive*
Permanent night shift workers	0.45 (0.07, 0.88)	0.18 (0.04, 0.40)	0.026	Super-additive*

RERI (Relative Excess Risk due to Interaction): Assesses the potential departure from additivity between genetic susceptibility and night shift work.AP (Attributable Proportion): Represents the proportion of the estimated risk in the combined exposure group that may be linked to the interaction between the two factors.Interaction Type: Categorized based on the 95% confidence interval (CI) of RERI and AP. P-value: Represents the significance level for the Relative Excess Risk due to Interaction (RERI). Given that RERI and AP are mathematically related, the statistical significance ($P < 0.05$) and the 95% confidence intervals for both metrics consistently indicate the same direction of departure from additivity.Additive: 95% CI crosses the null value (0), suggesting an independent and cumulative relationship.Super-additive: 95% CI is entirely above 0, reflecting a combined association that exceeds the sum of individual associations.Sub-additive: 95% CI is entirely below 0, reflecting a combined association that is less than the sum.

Supplementary Table 13. Joint association of PRS and Period spent working with CKD risk on an additive scale

Group	RERI (95% CI)	AP (95% CI)	*P* value	Interaction Type
**Intermediate genetic risk**				
<2years	0.17 (-0.18, 0.61)	0.11 (-0.11, 0.33)	0.391	Additive
2-14years	-0.19 (-0.72, 0.13)	-0.12 (-0.49, 0.08)	0.579	Additive
>14years	0.60 (-0.16, 0.96)	0.33 (-0.10, 0.49)	0.120	Additive
**High genetic risk**				
<2years	0.01 (-0.68, 0.55)	0.00 (-0.37, 0.21)	0.976	Additive
2-14years	-0.36 (-1.07, 0.13)	-0.16 (-0.51, 0.05)	0.239	Additive
>14years	0.17 (-0.72, 1.39)	0.08 (-0.44, 0.46)	0.753	Additive

RERI and AP were used to assess the departure from additivity. P-values represent the significance of RERI. Interaction types (Additive, Super-additive, or Sub-additive) were defined as per the criteria detailed in Table S12.

Supplementary Table 14. Joint association of PRS and Number of night shifts worked monthly with CKD risk on an additive scale

Group	RERI (95% CI)	AP (95% CI)	*P* value	Interaction Type
**Intermediate genetic risk**				
<5/month	-0.35 (-0.94, 0.13)	-0.25 (-0.73, 0.07)	0.197	Additive
5-10/month	-0.17 (-0.60, 0.17)	-0.11 (-0.42, 0.09)	0.374	Additive
>10/month	0.71 (0.15, 1.18)	0.35 (0.08, 0.54)	0.017	Super-additive*
**High genetic risk**				
<5/month	-0.45 (-1.23, -0.08)	-0.21 (-0.71, -0.03)	0.024	Sub-additive*
5-10/month	-0.08 (-0.74, 0.50)	-0.03 (-0.30, 0.17)	0.795	Additive
>10/month	-0.02 (-0.58, 0.87)	-0.01 (-0.29, 0.30)	0.967	Additive

RERI and AP were used to assess the departure from additivity. P-values represent the significance of RERI. Interaction types (Additive, Super-additive, or Sub-additive) were defined as per the criteria detailed in Table S12.

Supplementary Table 15. Joint association of PRS and Usual length of each night shift with CKD risk on an additive scale.

Group	RERI (95% CI)	AP (95% CI)	*P* value	Interaction Type
**Intermediate genetic risk**				
<8 h	0.21 (-0.84, 1.49)	0.09 (-0.47, 0.48)	0.721	Additive
8-12h	-0.01 (-0.28, 0.20)	-0.00 (-0.19, 0.11)	0.967	Additive
>12h	0.25 (-0.73, 0.88)	0.17 (-0.60, 0.56)	0.544	Additive
**High genetic risk**				
<8 h	-0.77 (-2.13, 0.44)	-0.35 (-1.63, 0.16)	0.240	Additive
8-12h	-0.05 (-0.45, 0.36)	-0.02 (-0.22, 0.13)	0.819	Additive
>12h	-0.51 (-1.58, 0.82)	-0.32 (-1.77, 0.35)	0.409	Additive

RERI and AP were used to assess the departure from additivity. P-values represent the significance of RERI. Interaction types (Additive, Super-additive, or Sub-additive) were defined as per the criteria detailed in Table S12.

Supplementary Table 16. Joint association of PRS and Consecutive night shifts with CKD risk on an additive scale

Group	RERI (95% CI)	AP (95% CI)	*P* value	Interaction Type
**Intermediate genetic risk**				
<2 shifts/month	-0.11 (-0.60, 0.47)	-0.08 (-0.53, 0.31)	0.698	Additive
2-7shifts/month	-0.06 (-0.36, 0.25)	-0.04 (-0.22, 0.14)	0.709	Additive
>7shifts/month	0.87 (-0.13, 1.50)	0.38 (-0.07, 0.60)	0.197	Additive
**High genetic risk**				
<2 shifts/month	-0.26 (-1.28, 0.51)	-0.13 (-1.02, 0.18)	0.562	Additive
2-7shifts/month	-0.14 (-0.57, 0.25)	-0.06 (-0.30, 0.09)	0.501	Additive
>7shifts/month	-0.13 (-1.12, 0.63)	-0.06 (-0.73, 0.23)	0.576	Additive

RERI and AP were used to assess the departure from additivity. P-values represent the significance of RERI. Interaction types (Additive, Super-additive, or Sub-additive) were defined as per the criteria detailed in Table S12.

Supplementary Table 17. Baseline characteristics according to categories of Current night shift work without covariate imputation (n=218246)

Characteristics	Total	Current night shift work		
		Never/rarely night shifts	Irregular night shift	Permanent night shift
No. participants	218246	200660	13303	4283
Age(years)±SD	52.18±6.97	52.32±6.98	50.50±6.64	50.59±6.59
sex(n,%)				
Female	114667(52.5)	107566(53.6)	5391(40.5)	1710(39.9)
Male	103579(47.5)	93094(46.4)	7912(59.5)	2573(60.1)
Ethnic(n,%)				
White	207087(94.9)	191368(95.4)	11831(88.9)	3888(90.8)
Asian	4434(2.0)	3849(1.9)	475(3.6)	110(2.6)
Black	3438(1.6)	2673(1.3)	587(4.4)	178(4.2)
Other	3287(1.5)	2770(1.4)	410(3.1)	107(2.5)
Education(n, %)				
College or University degree	93543(42.9)	89359(44.5)	3474(26.1)	710(16.6)
Other	124703(57.1)	111301(55.5)	9829(73.9)	3573(83.4)
Townsend deprivation index	-2.25(-3.70, 0.19)	-2.30(-3.73, 0.08)	-1.52(-3.30, 1.40)	-1.54(-3.30, 1.36)
BMI(n,%, kg/m2)				
Mean	26.37(23.85, 29.43)	26.28(23.79, 29.33)	27.29(24.66, 30.49)	27.69(25.04, 30.74)
<18.5	1009(0.5)	947（0.5）	49（0.4）	13（0.3）
18.5–24.9	78041(35.8)	71170（35.5）	3540（26.6）	1008（23.5）
25–29.9	91926(42.1)	85218（42.5）	5889（44.3）	1950（45.5）
≥30	47270(21.7)	43325（21.6）	3825（28.8）	1312（30.6）
Smoking(n,%)				
Never	128474(58.9)	118912(59.3)	7247(54.5)	2315(54.1)
Previous	68161(31.2)	62922(31.4)	3989(30.0)	1250(29.2)
Current	21611(9.9)	18826(9.4)	2067(15.5)	718(16.8)
Alcohol(n,%)				
Never	6336(2.9)	5518(2.7)	592(4.5)	226(5.3)
Previous	5476(2.5)	4929(2.5)	407(3.1)	140(3.3)
Current	206434(94.6)	190213(94.8)	12304(92.5)	3917(91.5)
Sleep duration(hours)	7.00(7.00, 8.00)	7.00(7.00, 8.00)	7.00(6.00, 8.00)	7.00(6.00, 8.00)
Healthy diet score±SD	2.81±1.30	2.82±1.29	2.67±1.32	2.61±1.29
Labor work(n,%)				
Never	150826(69.1)	145538(72.5)	4269(32.1)	1019(23.8)
Sometime	43753(20.0)	36679(18.3)	5448(41.0)	1626(38.0)
Usually	12672(5.8)	9938(5.0)	1960(14.7)	774(18.1)
Always	10995(5.0)	8505(4.2)	1626(12.2)	864(20.2)
Cancer(n,%)	17751(8.1)	16554(8.2)	903(6.8)	294(6.9)

Characteristics	Total	Current night shift work		
		Never/rarely night shifts	Irregular night shift	Permanent night shift
Hypertension(n,%)	44690(20.5)	40931(20.4)	2851(21.4)	908(21.2)
Diabetes(n,%)	7257(3.3)	6527(3.3)	571(4.3)	159(3.7)
Cardiovascular disease(n,%)	7660(3.5)	6992(3.5)	508(3.8)	160(3.7)
eGFR(mL/min per 1.73 m2)	96.27(87.05, 103.19)	96.15(86.94, 103.06)	97.83(88.30, 104.86)	97.48(88.42, 104.43)

Data were presented as frequency (%), mean ± standard deviation or median (interquartile range). Abbreviation: BMI, body mass index. eGFR, estimated Glomerular Filtration Rate；Data are mean ± SD or median (interquartile range) or frequencies (percentages).

Supplementary Table 18. Baseline characteristics of the study participants according to categories of Period spent working night shifts without covariate imputation (n=61567)

Characteristics	Total	Period spent working night shifts			
		None	<2years	2-14years	>14years
No. participants	61567	46893	5471	7509	1694
Age(years)±SD	52.7±6.8	52.9±6.8	51.8±6.9	52.4±6.7	52.6±6.4
sex(n,%)					
Female	33889(55.0)	27169(57.9)	2337(42.7)	3467(46.2)	916(54.1)
Male	27678(45.0)	19724(42.1)	3134(57.3)	4042(53.8)	778(45.9)
Ethnic(n,%)					
White	61567(100.0)	46893(100.0)	5471(100.0)	7509(100.0)	1694(100.0)
Education(n, %)					
College or University degree	32794(53.3)	26634(56.8)	2482(45.4)	2994(39.9)	684(40.4)
Other	28773(46.7)	20259(43.2)	2989(54.6)	4515(60.1)	1010(59.6)
Townsend deprivation index	-2.4(-3.9, -0.2)	-2.5(-3.9, -0.3)	-2.2(-3.7, 0.2)	-2.3(-3.7, 0.1)	-2.7(-3.9, -0.7)
BMI(n,%, kg/m2)					
Mean	25.9(23.5, 28.9)	25.7(23.4, 28.6)	26.6(24.1, 29.9)	26.7(24.0, 29.8)	26.7(24.0, 29.4)
<18.5	320(0.5)	277(0.6)	8(0.1)	25(0.3)	10(0.6)
18.5–24.9	24846(40.4)	19911(42.5)	1817(33.2)	2542(33.9)	576(34.0)
25–29.9	25046(40.7)	18815(40.1)	2318(42.4)	3162(42.1)	751(44.3)
≥30	11355(18.4)	7890(16.8)	1328(24.3)	1780(23.7)	357(21.1)
Smoking(n,%)					
Never	37515(60.9)	29442(62.8)	2964(54.2)	4128(55.0)	981(57.9)
Previous	19647(31.9)	14383(30.7)	1966(35.9)	2708(36.1)	590(34.8)
Current	4405(7.2)	3068(6.5)	541(9.9)	673(9.0)	123(7.3)
Alcohol(n,%)					
Never	1216(2.0)	966(2.1)	84(1.5)	131(1.7)	35(2.1)
Previous	1360(2.2)	984(2.1)	119(2.2)	202(2.7)	55(3.2)
Current	58991(95.8)	44943(95.8)	5268(96.3)	7176(95.6)	1604(94.7)
Sleep duration(hours)	7.0(7.0, 8.0)	7.0(7.0, 8.0)	7.0(6.0, 8.0)	7.0(6.0, 8.0)	7.0(7.0, 8.0)
Healthy diet score±SD	2.9±1.3	2.9±1.3	2.8±1.3	2.8±1.3	2.8±1.3
Labor work(n,%)					
Never	47416(77.0)	38268(81.6)	3637(66.5)	4541(60.5)	970(57.3)
Sometime	10158(16.5)	6406(13.7)	1228(22.4)	2027(27.0)	497(29.3)
Usually	2371(3.9)	1316(2.8)	341(6.2)	578(7.7)	136(8.0)
Always	1622(2.6)	903(1.9)	265(4.8)	363(4.8)	91(5.4)
Cancer(n,%)	5056(8.2)	3938(8.4)	376(6.9)	603(8.0)	139(8.2)
Hypertension(n,%)	11843(19.2)	8807(18.8)	1093(20.0)	1535(20.4)	408(24.1)
Diabetes(n,%)	1556(2.5)	1059(2.3)	185(3.4)	242(3.2)	70(4.1)
Cardiovascular disease(n,%)	1916(3.1)	1349(2.9)	240(4.4)	274(3.6)	53(3.1)

Characteristics	Total	Period spent working night shifts			
		None	<2years	2-14years	>14years
eGFR(mL/min per 1.73 m2)	95.7(86.5, 102.4)	95.6(86.4,102.3)	96.2(87.0,103.0)	96.0(86.9, 102.7)	95.6(86.6, 102.4)

Data were presented as frequency (%), mean ± standard deviation or median (interquartile range). Abbreviation: BMI, body mass index. eGFR, estimated Glomerular Filtration Rate；Data are mean ± SD or median (interquartile range) or frequencies (percentages).

Supplementary Table 19. Baseline characteristics of the study participants according to categories of Number of night shifts worked monthly without covariate imputation (n=61567)

Characteristics	Total	Number of night shifts worked monthly			
		None	<5/month	5-10/month	>10/month
No. participants	61567	46893	4043	6805	3826
Age(years)±SD	52.7±6.8	52.9±6.8	52.2±6.8	51.9±6.7	52.6±6.9
sex(n,%)					
Female	33889(55.0)	27169(57.9)	2014(49.8)	3008(44.2)	1698(44.4)
Male	27678(45.0)	19724(42.1)	2029(50.2)	3797(55.8)	2128(55.6)
Ethnic(n,%)					
White	61567(100.0)	46893(100.0)	4043(100.0)	6805(100.0)	3826(100.0)
Education(n, %)					
College or University degree	32794(53.3)	26634(56.8)	1874(46.4)	2857(42.0)	1429(37.3)
Other	28773(46.7)	20259(43.2)	2169(53.6)	3948(58.0)	2397(62.7)
Townsend deprivation index	-2.4(-3.9, -0.2)	-2.5(-3.9, -0.3)	-2.3(-3.7, -0.1)	-2.4(-3.8, -0.2)	-2.1(-3.7, 0.4)
BMI(n,%, kg/m2)					
Mean	25.9(23.5, 28.9)	25.7(23.3, 28.6)	26.4(23.8, 29.5)	26.6(24.0, 29.7)	27.0(24.4, 30.2)
<18.5	320(0.5)	277(0.6)	13(0.3)	19(0.3)	11(0.3)
18.5–24.9	24846(40.4)	19911(42.5)	1437(35.5)	2315(34.0)	1183(30.9)
25–29.9	25046(40.7)	18815(40.1)	1714(42.4)	2902(42.6)	1615(42.2)
≥30	11355(18.4)	7890(16.8)	879(21.7)	1569(23.1)	1017(26.6)
Smoking(n,%)					
Never	37515(60.9)	29442(62.8)	2261(55.9)	3874(56.9)	1938(50.7)
Previous	19647(31.9)	14383(30.7)	1446(35.8)	2368(34.8)	1450(37.9)
Current	4405(7.2)	3068(6.5)	336(8.3)	563(8.3)	438(11.4)
Alcohol(n,%)					
Never	1216(2.0)	966(2.1)	78(1.9)	106(1.6)	66(1.7)
Previous	1360(2.2)	984(2.1)	108(2.7)	168(2.5)	100(2.6)
Current	58991(95.8)	44943(95.8)	3857(95.4)	6531(96.0)	3660(95.7)
Sleep duration(hours)	7.0(7.0, 8.0)	7.00(7.0, 8.0)	7.00(7.0, 8.0)	7.00(7.0, 8.0)	7.00(6.0, 8.0)
Healthy diet score±SD	2.9±1.3	2.9±1.3	2.8±1.3	2.8±1.3	2.8±1.3
Labor work(n,%)					
Never	47416(77.0)	38268(81.6)	2596(64.2)	4381(64.4)	2171(56.7)
Sometime	10158(16.5)	6406(13.7)	1008(24.9)	1691(24.8)	1053(27.5)
Usually	2371(3.9)	1316(2.8)	282(7.0)	445(6.5)	328(8.6)
Always	1622(2.6)	903(1.9)	157(3.9)	288(4.2)	274(7.2)
Cancer(n,%)	5056(8.2)	3938(8.4)	328(8.1)	493(7.2)	297(7.8)
Hypertension(n,%)	11843(19.2)	8807(18.8)	818(20.2)	1374(20.2)	844(22.1)
Diabetes(n,%)	1556(2.5)	1059(2.3)	130(3.2)	219(3.2)	148(3.9)
Cardiovascular disease(n,%)	1916(3.1)	1349(2.9)	129(3.2)	271(4.0)	167(4.4)

Characteristics	Total	Number of night shifts worked monthly			
		None	<5/month	5-10/month	>10/month
eGFR(mL/min per 1.73 m2)	95.7(86.5, 102.4)	95.57(86.4, 102.3)	96.35(87.3, 102.9)	95.97(86.9, 102.8)	95.75(86.6, 102.7)

Data were presented as frequency (%), mean ± standard deviation or median (interquartile range). Abbreviation: BMI, body mass index. eGFR, estimated Glomerular Filtration Rate； Data are mean ± SD or median (interquartile range) or frequencies (percentages).

Supplementary Table 20. Baseline characteristics of the study participants according to categories of Usual length of each night shift without covariate imputation (n=61567)

Characteristics	Total	Usual length of each night shift			
		None	<8h	8-12h	>12h
No. participants	61567	46893	1625	11957	1092
Age(years)±SD	52.7±6.8	52.9±6.8	52.8±7.2	52.2±6.7	51.4±6.3
sex(n,%)					
Female	33889(55.0)	27169(57.9)	635(39.1)	5536(46.3)	549(50.3)
Male	27678(45.0)	19724(42.1)	990(60.9)	6421(53.7)	543(49.7)
Ethnic(n,%)					
White	61567(100.0)	46893(100.0)	1625(100.0)	11957(100.0)	1092(100.0)
Education(n, %)					
College or University degree	32794(53.3)	26634(56.8)	783(48.2)	4669(39.0)	708(64.8)
Other	28773(46.7)	20259(43.2)	842(51.8)	7288(61.0)	384(35.2)
Townsend deprivation index	-2.4(-3.9, -0.2)	-2.5(-3.9, -0.3)	-2.0(-3.6, 0.7)	-2.3(-3.7, -0.1)	-2.4(-3.8, -0.3)
BMI(n,%, kg/m2)					
Mean	25.9(23.5, 28.9)	25.7(23.4, 28.6)	26.5(24.1, 29.8)	26.7(24.1, 29.8)	25.8(23.3, 29.0)
<18.5	320(0.5)	277(0.6)	5(0.3)	31(0.3)	7(0.6)
18.5–24.9	24846(40.4)	19911(42.5)	546(33.6)	3941(33.0)	448(41.0)
25–29.9	25046(40.7)	18815(40.1)	693(42.6)	5121(42.8)	417(38.2)
≥30	11355(18.4)	7890(16.8)	381(23.4)	2864(24.0)	220(20.1)
Smoking(n,%)					
Never	37515(60.9)	29442(62.8)	833(51.3)	6544(54.7)	696(63.7)
Previous	19647(31.9)	14383(30.7)	648(39.9)	4294(35.9)	322(29.5)
Current	4405(7.2)	3068(6.5)	144(8.9)	1119(9.4)	74(6.8)
Alcohol(n,%)					
Never	1216(2.0)	966(2.1)	26(1.6)	202(1.7)	22(2.0)
Previous	1360(2.2)	984(2.1)	37(2.3)	320(2.7)	19(1.7)
Current	58991(95.8)	44943(95.8)	1562(96.1)	11435(95.6)	1051(96.2)
Sleep duration(hours)	7.0(7.0, 8.0)	7.0(7.0, 8.0)	7.0(7.0, 8.0)	7.0(6.0, 8.0)	7.0(7.0, 8.0)
Healthy diet score±SD	2.9±1.3	2.9±1.3	2.9±1.3	2.8±1.3	2.9±1.3
Labor work(n,%)					
Never	47416(77.0)	38268(81.6)	1092(67.2)	7269(60.8)	787(72.1)
Sometime	10158(16.5)	6406(13.7)	381(23.4)	3163(26.5)	208(19.0)
Usually	2371(3.9)	1316(2.8)	83(5.1)	908(7.6)	64(5.9)
Always	1622(2.6)	903(1.9)	69(4.2)	617(5.2)	33(3.0)
Cancer(n,%)	5056(8.2)	3938(8.4)	129(7.9)	900(7.5)	89(8.2)
Hypertension(n,%)	11843(19.2)	8807(18.8)	357(22.0)	2503(20.9)	176(16.1)
Diabetes(n,%)	1556(2.5)	2.88(1.28)	2.86(1.29)	2.80(1.30)	2.88(1.27)
Cardiovascular disease(n,%)	1916(3.1)	1349(2.9)	63(3.9)	473(4.0)	31(2.8)

Characteristics	Total	Usual length of each night shift			
		None	<8h	8-12h	>12h
eGFR(mL/min per 1.73 m2)	95.7(86.5, 102.4)	95.6(86.4, 102.3)	96.0(87.5, 102.5)	96.0(86.9, 102.9)	96.8(87.3, 102.7)

Data were presented as frequency (%), mean ± standard deviation or median (interquartile range).

Abbreviation: BMI, body mass index. eGFR, estimated Glomerular Filtration Rate；Data are mean ± SD or median (interquartile range) or frequencies (percentages).

Supplementary Table 21. Baseline characteristics of the study participants according to categories of Consecutive night shifts without covariate imputation (n=61567)

Characteristics	Total	Consecutive night shifts			
		None	2 shifts/month	2-7shifts/month	>7shifts/month
No. participants	61567	46893	2485	10762	1427
Age(years)±SD	52.7±6.8	52.9±6.8	52.3±6.9	52.0±6.7	53.4±6.9
sex(n,%)					
Female	33889(55.0)	27169(57.9)	1259(50.7)	4827(44.9)	634(44.4)
Male	27678(45.0)	19724(42.1)	1226(49.3)	5935(55.1)	793(55.6)
Ethnic(n,%)					
White	61567(100.0)	46893(100.0)	2485(100.0)	10762(100.0)	1427(100.0)
Education(n, %)					
College or University degree	32794(53.3)	26634(56.8)	1387(55.8)	4181(38.8)	592(41.5)
Other	28773(46.7)	20259(43.2)	1098(44.2)	6581(61.2)	835(58.5)
Townsend deprivation index	-2.4(-3.9, -0.2)	-2.49(-3.9, -0.3)	-2.2(-3.7, 0.2)	-2.3(-3.7, -0.0)	-2.3(-3.7, -0.0)
BMI(n,%, kg/m2)					
Mean	25.9(23.5, 28.9)	25.7(23.3, 28.6)	26.0(23.4, 29.0)	26.8(24.2, 29.9)	26.9(24.4, 30.1)
<18.5	320(0.5)	277(0.6)	7(0.3)	29(0.3)	7(0.5)
18.5–24.9	24846(40.4)	19911(42.5)	995(40.0)	3511(32.6)	429(30.1)
25–29.9	25046(40.7)	18815(40.1)	1003(40.4)	4601(42.8)	627(43.9)
≥30	11355(18.4)	7890(16.8)	480(19.3)	2621(24.4)	364(25.5)
Smoking(n,%)					
Never	37515(60.9)	29442(62.8)	1420(57.1)	5920(55.0)	733(51.4)
Previous	19647(31.9)	14383(30.7)	861(34.6)	3831(35.6)	572(40.1)
Current	4405(7.2)	3068(6.5)	204(8.2)	1011(9.4)	122(8.5)
Alcohol(n,%)					
Never	1216(2.0)	966(2.1)	43(1.7)	187(1.7)	20(1.4)
Previous	1360(2.2)	984(2.1)	67(2.7)	259(2.4)	50(3.5)
Current	58991(95.8)	44943(95.8)	2375(95.6)	10316(95.9)	1357(95.1)
Sleep duration(hours)	7.0(7.0, 8.0)	7.00(7.0, 8.0)	7.0(7.0, 8.0)	7.00(6.0, 8.0)	7.00(6.0, 8.0)
Healthy diet score±SD	2.9±1.3	2.9±1.3	2.9±1.3	2.8±1.3	2.8±1.3
Labor work(n,%)					
Never	47416(77.0)	38268(81.6)	1729(69.6)	6537(60.7)	882(61.8)
Sometime	10158(16.5)	6406(13.7)	515(20.7)	2858(26.6)	379(26.6)
Usually	2371(3.9)	1316(2.8)	151(6.1)	807(7.5)	97(6.8)
Always	1622(2.6)	903(1.9)	90(3.6)	560(5.2)	69(4.8)
Cancer(n,%)	5056(8.2)	3938(8.4)	191(7.7)	816(7.6)	111(7.8)
Hypertension(n,%)	11843(19.2)	8807(18.8)	463(18.6)	2247(20.9)	326(22.8)
Diabetes(n,%)	1556(2.5)	1059(2.3)	83(3.3)	360(3.3)	54(3.8)
Cardiovascular disease(n,%)	1916(3.1)	1349(2.9)	64(2.6)	435(4.0)	68(4.8)

Characteristics	Total	Consecutive night shifts			
		None	2 shifts/month	2-7shifts/month	>7shifts/month
eGFR(mL/min per 1.73 m2)	95.7(86.5, 102.4)	95.6(86.4, 102.3)	96.0(87.5, 102.7)	96.1(87.0, 103.0)	95.6(86.0, 101.9)

Data were presented as frequency (%), mean ± standard deviation or median (interquartile range).

Abbreviation: BMI, body mass index. eGFR, estimated Glomerular Filtration Rate；Data are mean ± SD or median (interquartile range) or frequencies (percentages).

Supplementary Table 22. Associations between the Current night shift work and risks of CKD without covariate imputation(n=218246)

	Current night shift work			
	Never/rarely night shifts	Irregular shift workers	Permanent night shift workers	
No. participants	200660	13303	4283	
No. cases(n,%)	7625(3.80)	543(4.08)	205(4.79)	
	HR	HR(95n,% CI)	HR(95n,% CI)	[d]*P* value
Unadjusted model	1.00 (ref.)	1.08(1.00-1.18)	1.26(1.10-1.45)	0.002
[a]Model 1	1.00(ref.)	1.21(1.11-1.32)	1.41(1.23-1.62)	<0.001
[b]Model 2	1.00(ref.)	1.08(0.99-1.18)	1.21(1.05-1.39)	<0.001
[c]Model 3	1.00(ref.)	1.07(0.97-1.17)	1.22(1.05-1.40)	0.016

[a]Model 1:adjusted for gender, age and race;

[b]Model 2: adjusted for model 1 plus BMI, alcohol , smoking status, education, and the Townsend deprivation index;

[c]Model 3: adjusted for model 2 plus healthy diet score, labor work level, and baseline diseases (including hypertension, diabetes, CVD, and cancer).

[d]*P* values: represent the overall significance of the categorical variable, calculated using the Likelihood Ratio Test (LRT) comparing models with and without the variable.

Supplementary Table 23. Associations between the Period spent working night shifts and risks of CKD without covariate imputation(n=61567)

	Period spent working night shifts				
	None	<2years	2-14years	>14years	
No. participants	46893	5471	7509	1694	
No. cases(n,%)	1471(3.14)	227(4.15)	321(4.27)	70(4.13)	
	HR	HR(95n,% CI)	HR(95n,% CI)	HR(95n,% CI)	[d]*P* value
Unadjusted model	1.00 (ref.)	1.33(1.15-1.54)	1.37(1.21-1.55)	1.36(1.06-1.74)	<0.001
[a]Model 1	1.00(ref.)	1.39(1.21-1.60)	1.40(1.24-1.58)	1.36(1.07-1.73)	<0.001
[b]Model 2	1.00(ref.)	1.27(1.10-1.46)	1.29(1.14-1.46)	1.27(1.00-1.62)	<0.001
[c]Model 3	1.00(ref.)	1.24(1.07-1.43)	1.26(1.11-1.43)	1.21(0.95-1.54)	0.002

[a]Model 1:adjusted for gender, age and race;

[b]Model 2: adjusted for model 1 plus BMI, alcohol , smoking status, education, and the Townsend deprivation index;

[c]Model 3: adjusted for model 2 plus healthy diet score, labor work level, and baseline diseases (including hypertension, diabetes, CVD, and cancer).

[d]*P* values: represent the overall significance of the categorical variable, calculated using the Likelihood Ratio Test (LRT) comparing models with and without the variable.

Supplementary Table 24. Associations between the Number of night shifts worked monthly and risks of CKD without covariate imputation(n=61567)

	Number of night shifts worked monthly				
	None	<5/month	5-10/month	>10/month	
No. participants	46893	4043	6805	3826	
No. cases(n,%)	1471(3.14)	157(3.88)	277(4.07)	184(4.81)	
	HR	HR(95n,% CI)	HR(95n,% CI)	HR(95n,% CI)	[d]*P* value
Unadjusted model	1.00 (ref.)	1.28(1.09-1.52)	1.28(1.12-1.46)	1.56(1.33-1.83)	<0.001
[a]Model 1	1.00(ref.)	1.28(1.09-1.51)	1.38(1.21-1.57)	1.53(1.31-1.79)	<0.001
[b]Model 2	1.00(ref.)	1.20(1.02-1.42)	1.28(1.12-1.45)	1.36(1.17-1.59)	<0.001
[c]Model 3	1.00(ref.)	1.18(1.00-1.39)	1.25(1.10-1.42)	1.31(1.12-1.54)	0.001

[a]Model 1:adjusted for gender, age and race;

[b]Model 2: adjusted for model 1 plus BMI, alcohol , smoking status, education, and the Townsend deprivation index;

[c]Model 3: adjusted for model 2 plus healthy diet score, labor work level, and baseline diseases (including hypertension, diabetes, CVD, and cancer).

[d]*P* values: represent the overall significance of the categorical variable, calculated using the Likelihood Ratio Test (LRT) comparing models with and without the variable.

Supplementary Table 25. Associations between the Usual length of each night shift and risks of CKD without covariate imputation(n=61567)

	Usual length of each night shift				
	None	<8 h	8-12h	>12h	
No. participants	46893	1625	11957	1092	
No. cases(n,%)	1471(3.14)	89(5.48)	498(4.16)	31(2.84)	
	HR	HR(95n,% CI)	HR(95n,% CI)	HR(95n,% CI)	[d]*P* value
Unadjusted model	1.00 (ref.)	1.83(1.48-2.27)	1.33(1.20-1.48)	0.82(0.56-1.22)	<0.001
[a]Model 1	1.00(ref.)	1.72(1.39-2.13)	1.38(1.25-1.53)	1.00(0.70-1.43)	<0.001
[b]Model 2	1.00(ref.)	1.57(1.27-1.95)	1.26(1.14-1.40)	0.96(0.67-1.37)	<0.001
[c]Model 3	1.00(ref.)	1.56(1.26-1.94)	1.23(1.10-1.36)	0.97(0.68-1.38)	<0.001

[a]Model 1:adjusted for gender, age and race;

[b]Model 2: adjusted for model 1 plus BMI, alcohol , smoking status, education, and the Townsend deprivation index;

[c]Model 3: adjusted for model 2 plus healthy diet score, labor work level, and baseline diseases (including hypertension, diabetes, CVD, and cancer).

[d]*P* values: represent the overall significance of the categorical variable, calculated using the Likelihood Ratio Test (LRT) comparing models with and without the variable.

Supplementary Table 26. Associations between the Usual length of each night shift and risks of CKD without covariate imputation(n=61567)

	Consecutive night shifts				
	None	<2 shifts/month	2-7shifts/month	>7shifts/month	
No. participants	46893	2485	10762	1427	
No. cases(n,%)	1471（3.14）	80（3.22）	458（4.26）	80（5.61）	
	HR	HR(95n,% CI)	HR(95n,% CI)	HR(95n,% CI)	[d]*P* value
Unadjusted model	1.00 (ref.)	1.03(0.81-1.29)	1.37(1.23-1.53)	1.802(1.43-2.27)	<0.001
[a]Model 1	1.00(ref.)	1.05(0.84-1.32)	1.43(1.29-1.59)	1.70(1.36-2.13)	<0.001
[b]Model 2	1.00(ref.)	1.01(0.81-1.27)	1.31(1.18-1.45)	1.51(1.20-1.89)	<0.001
[c]Model 3	1.00(ref.)	1.00(0.80-1.26)	1.27(1.14-1.42)	1.47(1.17-1.84)	<0.001

[a]Model 1:adjusted for gender, age and race;

[b]Model 2: adjusted for model 1 plus BMI, alcohol , smoking status, education, and the Townsend deprivation index;

[c]Model 3: adjusted for model 2 plus healthy diet score, labor work level, and baseline diseases (including hypertension, diabetes, CVD, and cancer).

[d]*P* values: represent the overall significance of the categorical variable, calculated using the Likelihood Ratio Test (LRT) comparing models with and without the variable.

Supplementary Table 27. Association of Current night shift work with CKD Risk After Excluding CKD Cases Within the First Two Years (n=250781)

	Current night shift work			
	Never/rarely night shifts	Irregular night shift	Permanent night shift	
No. participants	228803	16446	5532	
No. cases(n,%)	8627(3.77)	664(4.04)	282(5.10)	
	HR	HR(95n,% CI)	HR(95n,% CI)	[d]*P* value
Unadjusted model	1.00 (ref.)	1.08(1.00-1.17)	1.36(1.21-1.53)	<0.001
[a]Model 1	1.00(ref.)	1.21(1.11-1.31)	1.50(1.33-1.69)	<0.001
[b]Model 2	1.00(ref.)	1.10(1.01-1.19)	1.32(1.17-1.49)	<0.001
[c]Model 3	1.00(ref.)	1.05(0.97-1.14)	1.26(1.11-1.42)	0.001

[a]Model 1:adjusted for gender, age and race;

[b]Model 2: adjusted for model 1 plus BMI, alcohol , smoking status, education, and the Townsend deprivation index;

[c]Model 3: adjusted for model 2 plus healthy diet score, labor work level, and baseline diseases (including hypertension, diabetes, CVD, and cancer).

[d]*P* values: represent the overall significance of the categorical variable, calculated using the Likelihood Ratio Test (LRT) comparing models with and without the variable.

Supplementary Table 28. Association of Period spent working night shifts with CKD Risk After Excluding CKD Cases Within the First Two Years (n=67694)

	Period spent working night shifts				
	None	<2years	2-14years	>14years	
No. participants	51246	6127	8412	1909	
No. cases(n,%)	1548(3.02)	235(3.84)	335(3.98)	76(3.98)	
	HR	HR(95n,% CI)	HR(95n,% CI)	HR(95n,% CI)	[d]*P* value
Unadjusted model	1.00 (ref.)	1,27(1.11-1.46)	1.33(1.18-1.50)	1.33(1.05-1.67)	<0.001
[a]Model 1	1.00(ref.)	1.32(1.15 - 1.51)	1.34(1.19 - 1.51)	1.33(1.06 - 1.68)	<0.001
[b]Model 2	1.00(ref.)	1.20(1.05 - 1.38)	1.23(1.09 - 1.38)	1.25(0.99 - 1.58)	<0.001
[c]Model 3	1.00(ref.)	1.17(1.02 - 1.35)	1.20(1.06 - 1.35)	1.19(0.94 - 1.50)	0.007

[a]Model 1:adjusted for gender, age and race;

[b]Model 2: adjusted for model 1 plus BMI, alcohol , smoking status, education, and the Townsend deprivation index;

[c]Model 3: adjusted for model 2 plus healthy diet score, labor work level, and baseline diseases (including hypertension, diabetes, CVD, and cancer).

[d]*P* values: represent the overall significance of the categorical variable, calculated using the Likelihood Ratio Test (LRT) comparing models with and without the variable.

Supplementary Table 29. Association of Number of night shifts worked monthly with CKD Risk After Excluding CKD Cases Within the First Two Years (n=67694)

	Number of night shifts worked monthly				
	None	<5/month	5-10/month	>10/month	
No. participants	51246	4526	7537	4385	
No. cases(n,%)	1548(3.02)	162(3.58)	288(3.82)	196(4.47)	
	HR	HR(95n,% CI)	HR(95n,% CI)	HR(95n,% CI)	[d]*P* value
Unadjusted model	1.00 (ref.)	1.19(1.01-1.40)	1.27(1.12-1.45)	1.49(1.28-1.73)	<0.001
[a]Model 1	1.00(ref.)	1.21(1.03 - 1.42)	1.33(1.17 - 1.51)	1.45(1.25 - 1.68)	<0.001
[b]Model 2	1.00(ref.)	1.14(0.97 - 1.34)	1.23(1.08 - 1.40)	1.29(1.11 - 1.50)	<0.001
[c]Model 3	1.00(ref.)	1.11(0.95 - 1.31)	1.20(1.05 - 1.36)	1.24(1.06 - 1.44)	0.005

[a]Model 1:adjusted for gender, age and race;

[b]Model 2: adjusted for model 1 plus BMI, alcohol , smoking status, education, and the Townsend deprivation index;

[c]Model 3: adjusted for model 2 plus healthy diet score, labor work level, and baseline diseases (including hypertension, diabetes, CVD, and cancer).

[d]*P* values: represent the overall significance of the categorical variable, calculated using the Likelihood Ratio Test (LRT) comparing models with and without the variable.

Supplementary Table 30. Association of Usual length of each night shift with CKD Risk After Excluding CKD Cases Within the First Two Years (n=67694)

	Usual length of each night shift				
	None	<8 h	8-12h	>12h	
No. participants	51246	1817	13441	1190	
No. cases(n,%)	1548(3.02)	94(5.17)	520(3.87)	32(2.69)	
	HR	HR(95n,% CI)	HR(95n,% CI)	HR(95n,% CI)	[d]*P* value
Unadjusted model	1.00 (ref.)	1.74(1.42-2.15)	1.29(1.17-1.42)	0.89(0.62-1.26)	<0.001
[a]Model 1	1.00(ref.)	1.67(1.36 - 2.06)	1.31(1.18 - 1.45)	0.99(0.70 - 1.41)	<0.001
[b]Model 2	1.00(ref.)	1.53(1.24 - 1.89)	1.20(1.08 - 1.33)	0.95(0.67 - 1.36)	<0.001
[c]Model 3	1.00(ref.)	1.51(1.22 - 1.86)	1.16(1.04 - 1.29)	0.97(0.68 - 1.37)	<0.001

[a]Model 1:adjusted for gender, age and race;

[b]Model 2: adjusted for model 1 plus BMI, alcohol , smoking status, education, and the Townsend deprivation index;

[c]Model 3: adjusted for model 2 plus healthy diet score, labor work level, and baseline diseases (including hypertension, diabetes, CVD, and cancer).

[d]*P* values: represent the overall significance of the categorical variable, calculated using the Likelihood Ratio Test (LRT) comparing models with and without the variable.

Supplementary Table 31. Association of Consecutive night shifts with CKD Risk After Excluding CKD Cases Within the First Two Years (n=67694)

	Consecutive night shifts				
	None	<2 shifts/month	2-7shifts/month	>7shifts/month	
No. participants	51246	2738	12106	1604	
No. cases(n,%)	1548(3.02)	82(2.99)	489(4.04)	75(4.68)	
	HR	HR(95n,% CI)	HR(95n,% CI)	HR(95n,% CI)	[d]*P* value
Unadjusted model	1.00 (ref.)	0.99(0.79-1.24)	1.35(1.22-1.49)	1.56(1.24-1.97)	<0.001
[a]Model 1	1.00(ref.)	1.01(0.81 - 1.26)	1.38(1.25 - 1.53)	1.46(1.16 - 1.84)	<0.001
[b]Model 2	1.00(ref.)	0.97(0.77 - 1.21)	1.27(1.14 - 1.41)	1.30(1.03 - 1.64)	<0.001
[c]Model 3	1.00(ref.)	1.96(0.77 - 1.20)	1.23(1.10 - 1.37)	1.26(1.00 - 1.59)	<0.001

[a]Model 1:adjusted for gender, age and race;

[b]Model 2: adjusted for model 1 plus BMI, alcohol , smoking status, education, and the Townsend deprivation index;

[c]Model 3: adjusted for model 2 plus healthy diet score, labor work level, and baseline diseases (including hypertension, diabetes, CVD, and cancer).

[d]*P* values: represent the overall significance of the categorical variable, calculated using the Likelihood Ratio Test (LRT) comparing models with and without the variable.

Supplementary Table 32. Stratified Analysis of Current night shift work and CKD Risk by covariates

Characteristics	Current night shift work			*P* value	*P* interaction
	Never/rarely night shifts	Irregular shift workers	Permanent night shift workers		
Sex					
Women	1.00	1.06(0.93 - 1.20)	1.34(1.11 - 1.61)	0.002	0.11
Man	1.00	1.07(0.97 - 1.18)	1.12(0.96 - 1.30)		
Sleep duration					
≤6 h/day	1.00	1.03(0.91 - 1.17)	1.16(0.97 - 1.39)	0.003	0.61
7-8 h/day	1.00	1.07(0.97 - 1.18)	1.27(1.08 - 1.50)		
≥9 h/day	1.00	1.18(0.87 - 1.59)	0.76(0.41 - 1.38)		
Age					
<60years	1.00	1.04(0.95 - 1.14)	1.13(0.98 - 1.31)	0.086	0.44
>=60years	1.00	0.99(0.85 - 1.14)	1.20(0.97 - 1.47)		
Smoking					
Never	1.00	1.02(0.91 - 1.14)	1.21(1.02 - 1.44)	0.014	0.10
Previous	1.00	1.16(1.03 - 1.32)	1.28(1.05 - 1.55)		
Current	1.00	0.95(0.78 - 1.15)	0.97(0.72 - 1.31)		
Body mass index					
<18.5	1.00	0.58(0.07 - 4.52)	2.97(0.70 - 12.53)	0.037	0.35
18.5-25	1.00	1.20(1.01 - 1.43)	1.30(0.97 - 1.73)		
25-30	1.00	1.04(0.92 - 1.17)	1.21(1.01 - 1.44)		
>30	1.00	1.02(0.90 - 1.15)	1.13(0.94 - 1.36)		
Labor work					
Never	1.00	1.03(0.90 - 1.18)	1.46(1.17 - 1.81)	<0.001	0.11
Sometime	1.00	1.08(0.96 - 1.22)	1.11(0.91 - 1.35)		
Usually	1.00	1.11(0.92 - 1.36)	1.05(0.77 - 1.42)		
Always	1.00	0.92(0.74 - 1.14)	1.13(0.87 - 1.47)		
Healthy diet					
<4	1.00	1.08(0.99 - 1.18)	1.24(1.08 - 1.42)	0.002	0.44
>=4	1.00	1.00(0.86 - 1.16)	1.05(0.82 - 1.35)		
Chronotype					
Morning	1.00	1.14(0.99 - 1.31)	1.03(0.80 - 1.33)	0.028	0.66
Intermediate	1.00	0.98(0.85 - 1.13)	1.23(0.98 - 1.55)		
Evening	1.00	1.16(0.93 - 1.44)	1.34(1.03 - 1.74)		
Alcohol				0.007	0.33
Never	1.00	1.23(0.91 - 1.67)	1.47(0.95 - 2.27)		

Characteristics	Current night shift work			P value	P interaction
	Never/rarely night shifts	Irregular shift workers	Permanent night shift workers		
Previous	1.00	0.94(0.64 - 1.37)	1.07(0.63 - 1.81)		
Current	1.00	1.05(0.97 - 1.14)	1.19(1.05 - 1.35)		
Education					
College or University degree	1.00	1.02(0.87 - 1.20)	1.13(0.83 - 1.55)	0.003	0.91
Other	1.00	1.07(0.98 - 1.17)	1.21(1.07 - 1.37)		
Cancer					
No	1.00	1.07(0.99-1.16)	1.18(1.04-1.34)	0.008	0.95
Yes	1.00	0.94(0.73 - 1.21)	1.26(0.88 - 1.80)		
Hypertension					
No	1.00	1.10(0.99-1.21)	1.30(1.12-1.51)	<0.001	0.02
Yes	1.00	1.00(0.88 - 1.13)	1.04(0.86 - 1.27)		
Diabetes					
No	1.00	1.07(0.98-1.16)	1.24(1.09-1.40)	<0.001	0.03
Yes	1.00	0.98(0.79 - 1.22)	0.89(0.60 - 1.31)		
Cardiovascular disease				0.006	<0.001
No	1.00	1.08(0.99-1.17)	1.19(1.05-1.35)		
Yes	1.00	0.86(0.67 - 1.12)	1.23(0.86 - 1.77)		
Ethnic	1.00				
White	1.00	1.08(0.99 - 1.17)	1.25(1.11 - 1.42)		
Asian	1.00	0.99(0.67 - 1.46)	0.55(0.20 - 1.50)	<0.001	<0.001
Black	1.00	0.68(0.45 - 1.02)	0.86(0.47 - 1.58)		
Other	1.00	1.09(0.69 - 1.71)	0.79(0.31 - 1.97)		

Cox models were adjusted for gender, age, race, BMI, alcohol, smoking status, education, the Townsend deprivation index, healthy diet score, labor work level, and baseline diseases (including hypertension, diabetes, CVD, and cancer).

Supplementary Table 33. Stratified Analysis of Period spent working night shifts and CKD Risk by covariates

Characteristics	Period spent working night shifts				*P* value	*P* interaction
	None	<2years	2-14years	>14years		
Sex						
Women	1.00	1.20(0.97 - 1.50)	1.26(1.05 - 1.50)	1.23(0.88 - 1.72)	0.013	0.81
Man	1.00	1.13(0.96 - 1.34)	1.17(1.00 - 1.36)	1.08(0.80 - 1.47)		
Sleep duration						
≤6 h/day	1.00	1.10(0.85 - 1.42)	1.06(0.84 - 1.33)	1.34(0.91 - 2.00)	<0.001	0.44
7-8 h/day	1.00	1.20(1.02 - 1.41)	1.28(1.11 - 1.47)	1.00(0.74 - 1.34)		
≥9 h/day	1.00	1.08(0.58 - 2.02)	1.04(0.60 - 1.81)	1.60(0.73 - 3.49)		
Age						
<60years	1.00	1.16(0.99 - 1.37)	1.23(1.07 - 1.42)	1.13(0.85 - 1.50)	0.005	0.80
>=60years	1.00	1.05(0.84 - 1.33)	1.14(0.94 - 1.39)	1.17(0.80 - 1.70)		
Smoking						
Never	1.00	1.08(0.89 - 1.32)	1.27(1.07 - 1.49)	1.01(0.72 - 1.42)	0.004	0.01
Previous	1.00	1.13(0.92 - 1.40)	1.22(1.02 - 1.46)	1.49(1.08 - 2.04)		
Current	1.00	1.72(1.18 - 2.49)	0.97(0.65 - 1.45)	0.31(0.08 - 1.24)		
Body mass index						
<18.5	1.00	—	—	4.89(0.57 - 41.76)		
18.5-25	1.00	1.24(0.93 - 1.66)	1.40(1.11 - 1.78)	1.14(0.69 - 1.89)	0.005	0.64
25-30	1.00	1.26(1.03 - 1.54)	1.10(0.91 - 1.32)	1.13(0.81 - 1.58)		
>30	1.00	1.04(0.82 - 1.31)	1.24(1.02 - 1.51)	1.13(0.76 - 1.68)		
Labor work						
Never	1.00	1.20(1.02 - 1.42)	1.20(1.04 - 1.39)	1.28(0.96 - 1.70)		
Sometime	1.00	1.28(0.98 - 1.69)	1.23(0.97 - 1.56)	0.89(0.55 - 1.43)	0.015	0.37
Usually	1.00	0.81(0.44 - 1.49)	0.92(0.58 - 1.46)	0.88(0.38 - 2.06)		
Always	1.00	0.62(0.30 - 1.28)	1.42(0.87 - 2.32)	1.29(0.54 - 3.06)		
Healthy diet						
<4	1.00	1.21(1.03 - 1.42)	1.29(1.12 - 1.49)	1.23(0.94 - 1.60)	<0.001	0.16
>=4	1.00	1.10(0.86 - 1.40)	1.07(0.86 - 1.32)	0.98(0.63 - 1.50)		
Chronotype						
Morning	1.00	1.06(0.80 - 1.40)	1.29(1.04 - 1.60)	0.94(0.55 - 1.61)	0.007	0.48
Intermediate	1.00	1.25(1.06 - 1.48)	1.21(1.04 - 1.40)	1.22(0.94 - 1.60)		
Evening	1.00	1.00(0.65 - 1.55)	1.06(0.72 - 1.57)	1.09(0.55 - 2.15)		
Alcohol					0.003	0.98
Never	1.00	1.12(0.46 - 2.73)	1.26(0.58 - 2.76)	1.43(0.33 - 6.16)		
Previous	1.00	1.29(0.60 - 2.78)	1.67(0.93 - 3.00)	1.12(0.39 - 3.20)		

Characteristics	Period spent working night shifts				P value	$P_{interaction}$
	None	<2years	2-14years	>14years		
Current	1.00	1.17(1.02 - 1.34)	1.20(1.06 - 1.35)	1.14(0.90 - 1.44)		
Education						
College or University degree	1.00	1.21(0.98 - 1.49)	1.13(0.93 - 1.38)	1.05(0.71 - 1.54)	0.002	0.79
Other	1.00	1.14(0.96 - 1.36)	1.26(1.09 - 1.46)	1.19(0.90 - 1.57)		
Cancer						
Yes	1.00	1.09(0.71 - 1.67)	0.90(0.62 - 1.32)	0.82(0.38 - 1.74)	<0.001	0.47
No	1.00	1.18(1.03-1.36)	1.25(1.11-1.42)	1.18(0.93-1.50)		
Hypertension						
Yes	1.00	1.12(0.89 - 1.40)	1.28(1.06 - 1.55)	1.32(0.95 - 1.84)	0.012	0.48
No	1.00	1.20(1.02-1.42)	1.17(1.01-1.36)	1.00(0.73-1.37)		
Diabetes						
Yes	1.00	1.00(0.64 - 1.55)	1.10(0.75 - 1.62)	1.26(0.66 - 2.42)	0.001	0.85
No	1.00	1.19(1.04-1.37)	1.22(1.08-1.38)	1.14(0.90-1.45)		
Cardiovascular disease					<0.001	<0.001
Yes	1.00	1.15(0.76 - 1.72)	0.99(0.66 - 1.48)	1.26(0.61 - 2.58)		
No	1.00	1.18(1.02-1.36)	1.23(1.09-1.39)	1.13(0.89-1.43)		
Ethnic						
White	1.00	1.17(1.02 - 1.35)	1.22(1.08 - 1.37)	1.17(0.93 - 1.47)		
Asian	1.00	1.36(0.44 - 4.22)	0.32(0.04 - 2.57)	—	0.001	<0.001
Black	1.00	0.56(0.12 - 2.61)	1.27(0.37 - 4.30)	0.94(0.11 - 7.94)		
Other	1.00	1.53(0.58 - 4.03)	1.67(0.67 - 4.14)	—		

Cox models were adjusted for gender, age, race, BMI, alcohol, smoking status, education, the Townsend deprivation index, healthy diet score, labor work level, and baseline diseases (including hypertension, diabetes, CVD, and cancer). "—":unstable or inestimable due to few events in the subgroup.

Supplementary Table 34. Stratified Analysis of Number of night shifts worked monthly and CKD Risk by covariates

Characteristics	Number of night shifts worked monthly				*P* value	*P* interaction
	None	<5/month	5-10/month	>10/month		
Sex						
Women	1.00	1.11(0.87 - 1.41)	1.25(1.03 - 1.52)	1.35(1.07 - 1.71)	0.010	0.66
Man	1.00	1.12(0.91 - 1.37)	1.16(0.99 - 1.37)	1.13(0.94 - 1.37)		
Sleep duration						
≤6 h/day	1.00	0.74(0.51 - 1.06)	1.11(0.87 - 1.41)	1.40(1.09 - 1.80)	0.006	0.08
7-8 h/day	1.00	1.28(1.07 - 1.53)	1.23(1.06 - 1.43)	1.13(0.94 - 1.37)		
≥9 h/day	1.00	1.08(0.53 - 2.17)	1.26(0.74 - 2.14)	0.99(0.47 - 2.08)		
Age						
<60years	1.00	1.14(0.94 - 1.38)	1.17(1.00 - 1.36)	1.29(1.08 - 1.55)	0.006	0.80
>=60years	1.00	1.02(0.78 - 1.34)	1.19(0.96 - 1.46)	1.09(0.85 - 1.39)		
Smoking						
Never	1.00	1.07(0.85 - 1.34)	1.12(0.94 - 1.35)	1.35(1.09 - 1.67)	0.006	0.50
Previous	1.00	1.14(0.90 - 1.46)	1.28(1.06 - 1.54)	1.20(0.96 - 1.50)		
Current	1.00	1.34(0.83 - 2.16)	1.30(0.88 - 1.93)	0.88(0.54 - 1.44)		
Body mass index						
<18.5	1.00	—	—	2.66(0.32 - 22.37)		
18.5-25	1.00	1.37(1.01 - 1.85)	1.42(1.11 - 1.82)	1.06(0.74 - 1.52)	0.006	0.22
25-30	1.00	1.07(0.84 - 1.36)	1.18(0.97 - 1.42)	1.23(0.98 - 1.53)		
>30	1.00	1.05(0.79 - 1.39)	1.12(0.90 - 1.39)	1.28(1.01 - 1.62)		
Labor work						
Never	1.00	1.23(1.02 - 1.49)	1.20(1.03 - 1.40)	1.20(0.99 - 1.46)		
Sometime	1.00	1.04(0.75 - 1.44)	1.20(0.93 - 1.55)	1.34(1.02 - 1.77)	0.018	0.15
Usually	1.00	0.43(0.18 - 1.00)	1.33(0.84 - 2.10)	0.73(0.40 - 1.33)		
Always	1.00	1.16(0.58 - 2.32)	0.82(0.43 - 1.56)	1.34(0.78 - 2.30)		
Healthy diet						
<4	1.00	1.16(0.96 - 1.40)	1.27(1.10 - 1.47)	1.31(1.10 - 1.56)	0.001	0.22
>=4	1.00	1.05(0.80 - 1.39)	1.08(0.86 - 1.35)	1.06(0.80 - 1.40)		
Chronotype						
Morning	1.00	0.97(0.70 - 1.35)	1.19(0.94 - 1.52)	1.32(1.00 - 1.75)	0.003	0.30
Intermediate	1.00	1.16(0.95 - 1.40)	1.26(1.08 - 1.47)	1.24(1.03 - 1.50)		
Evening	1.00	1.34(0.84 - 2.12)	0.91(0.59 - 1.40)	0.98(0.60 - 1.60)		
Alcohol						
Never	1.00	1.67(0.69 - 4.03)	1.02(0.39 - 2.65)	1.08(0.40 - 2.88)	0.005	0.78
Previous	1.00	0.96(0.38 - 2.43)	1.48(0.79 - 2.77)	1.95(0.95 - 4.00)		
Current	1.00	1.12(0.95 - 1.32)	1.20(1.06 - 1.36)	1.21(1.04 - 1.41)		
Education					0.004	0.77
College or University degree	1.00	1.16(0.91 - 1.48)	1.11(0.91 - 1.36)	1.21(0.94 - 1.56)		

Characteristics	Number of night shifts worked monthly				P value	$P_{interaction}$
	None	<5/month	5-10/month	>10/month		
Other	1.00	1.10(0.89 - 1.35)	1.26(1.08 - 1.47)	1.24(1.03 - 1.49)		
Cancer						
Yes	1.00	0.80(0.47 - 1.37)	0.81(0.53 - 1.26)	1.31(0.86 - 2.00)	<0.001	0.21
No	1.00	1.16(0.99-1.37)	1.25(1.10-1.43)	1.22(1.04-1.43)		
Hypertension						
Yes	1.00	1.50(1.19 - 1.89)	1.08(0.87 - 1.34)	1.20(0.95 - 1.52)	<0.001	<0.001
No	1.00	0.92(0.74-1.13)	1.27(1.09-1.48)	1.24(1.03-1.50)		
Diabetes						
Yes	1.00	1.15(0.71 - 1.87)	1.08(0.71 - 1.63)	1.04(0.66 - 1.64)	0.002	0.82
No	1.00	1.12(0.95-1.32)	1.22(1.08-1.39)	1.25(1.07-1.46)		
Cardiovascular disease					0.005	<0.001
Yes	1.00	0.95(0.54 - 1.66)	1.15(0.77 - 1.70)	1.09(0.69 - 1.72)		
No	1.00	1.15(0.97-1.35)	1.21(1.06-1.37)	1.24(1.06-1.45)		
Ethnic						
White	1.00	1.13(0.96 - 1.32)	1.20(1.06 - 1.36)	1.25(1.07 - 1.45)		
Asian	1.00	0.62(0.08 - 4.79)	0.85(0.19 - 3.89)	0.99(0.20 - 5.05)	0.004	<0.001
Black	1.00	1.68(0.51 - 5.51)	1.03(0.25 - 4.28)	—		
Other	1.00	0.87(0.24 - 3.08)	2.37(0.94 - 6.00)	1.30(0.40 - 4.21)		

Cox models were adjusted for gender, age, race, BMI, alcohol, smoking status, education, the Townsend deprivation index, healthy diet score, labor work level, and baseline diseases (including hypertension, diabetes, CVD, and cancer). "—":unstable or inestimable due to few events in the subgroup.

Supplementary Table 35. Stratified Analysis of Usual length of each night shift and CKD Risk by covariates

Characteristics	Usual length of each night shift				*P* value	*P* interaction
	None	<8 h	8-12h	>12h		
Sex						
Women	1.00	1.91(1.39 - 2.62)	1.17(1.01 - 1.37)	1.01(0.60 - 1.68)	<0.001	0.25
Man	1.00	1.29(0.99 - 1.67)	1.14(1.00 - 1.30)	0.89(0.57 - 1.41)		
Sleep duration						
≤6 h/day	1.00	1.15(0.75 - 1.78)	1.13(0.93 - 1.37)	0.66(0.29 - 1.47)	<0.001	0.76
7-8 h/day	1.00	1.66(1.31 - 2.11)	1.17(1.03 - 1.32)	1.04(0.71 - 1.54)		
≥9 h/day	1.00	1.28(0.51 - 3.22)	1.13(0.72 - 1.77)	0.99(0.24 - 4.07)		
Age						
<60years	1.00	1.47(1.13 - 1.91)	1.19(1.06 - 1.35)	0.81(0.53 - 1.23)	0.004	0.53
>=60years	1.00	1.47(1.07 - 2.03)	1.05(0.88 - 1.24)	1.26(0.71 - 2.24)		
Smoking						
Never	1.00	1.60(1.19 - 2.15)	1.10(0.95 - 1.27)	1.30(0.88 - 1.92)	0.002	0.17
Previous	1.00	1.39(1.02 - 1.90)	1.23(1.06 - 1.44)	0.55(0.26 - 1.15)		
Current	1.00	1.60(0.86 - 2.98)	1.17(0.85 - 1.60)	0.30(0.04 - 2.16)		
Body mass index						
<18.5	1.00	—	0.73(0.09 - 6.11)	—	<0.001	0.01
18.5-25	1.00	2.43(1.71 - 3.47)	1.15(0.93 - 1.43)	1.27(0.72 - 2.26)		
25-30	1.00	1.57(1.17 - 2.12)	1.11(0.95 - 1.29)	1.06(0.64 - 1.77)		
>30	1.00	0.91(0.59 - 1.41)	1.22(1.04 - 1.45)	0.56(0.27 - 1.19)		
Labor work						
Never	1.00	1.60(1.25 - 2.05)	1.17(1.04 - 1.33)	0.97(0.65 - 1.46)	<0.001	0.43
Sometime	1.00	1.48(0.99 - 2.22)	1.17(0.95 - 1.44)	1.02(0.50 - 2.07)		
Usually	1.00	0.94(0.34 - 2.60)	0.90(0.60 - 1.34)	0.45(0.06 - 3.23)		
Always	1.00	0.75(0.23 - 2.45)	1.16(0.74 - 1.80)	0.64(0.09 - 4.72)		
Healthy diet						
<4	1.00	1.53(1.19 - 1.96)	1.23(1.09 - 1.39)	1.06(0.72 - 1.57)	<0.001	0.43
>=4	1.00	1.49(1.06 - 2.11)	1.03(0.86 - 1.24)	0.71(0.35 - 1.42)		
Chronotype						
Morning	1.00	1.53(1.04 - 2.25)	1.13(0.93 - 1.38)	0.97(0.48 - 1.96)	0.002	0.52
Intermediate	1.00	1.47(1.14 - 1.91)	1.21(1.07 - 1.37)	0.99(0.66 - 1.51)		
Evening	1.00	1.71(0.94 - 3.08)	0.98(0.71 - 1.37)	0.62(0.20 - 1.96)		
Alcohol						
Never	1.00	1.11(0.26 - 4.70)	1.25(0.64 - 2.44)	1.13(0.15 - 8.35)	<0.001	0.81
Previous	1.00	2.05(0.73 - 5.78)	1.29(0.76 - 2.19)	2.74(0.83 - 9.03)		

Characteristics	Usual length of each night shift				*P* value	*P* interaction
	None	<8 h	8-12h	>12h		
Current	1.00	1.51(1.23 - 1.86)	1.16(1.05 - 1.28)	0.88(0.61 - 1.26)		
Education						
College or University degree	1.00	1.64(1.22 - 2.21)	1.08(0.91 - 1.27)	1.08(0.70 - 1.67)	0.001	0.47
Other	1.00	1.42(1.08 - 1.86)	1.21(1.07 - 1.37)	0.77(0.44 - 1.33)		
Cancer						
Yes	1.00	1.20(0.61 - 2.36)	0.96(0.70 - 1.31)	0.49(0.12 - 1.96)	<0.001	0.53
No	1.00	1.55(1.26-1.92)	1.19(1.07-1.32)	1.00(0.70-1.42)		
Hypertension						
Yes	1.00	1.20(0.84 - 1.73)	1.24(1.06 - 1.46)	1.00(0.55 - 1.83)	<0.001	0.26
No	1.00	1.70(1.33-2.17)	1.11(0.98-1.26)	0.91(0.60-1.37)		
Diabetes						
Yes	1.00	1.35(0.72 - 2.54)	1.09(0.79 - 1.51)	0.29(0.04 - 2.06)	<0.001	0.46
No	1.00	1.53(1.24-1.89)	1.17(1.06-1.30)	1.01(0.72-1.43)		
Cardiovascular disease					<0.001	<0.001
Yes	1.00	1.09(0.50 - 2.34)	1.02(0.74 - 1.41)	2.13(0.93 - 4.86)		
No	1.00	1.55(1.26-1.91)	1.18(1.06-1.31)	0.84(0.58-1.23)		
Ethnic						
White	1.00	1.51(1.22 - 1.85)	1.17(1.06 - 1.29)	0.99(0.70 - 1.39)		
Asian	1.00	1.85(0.23 - 14.84)	0.82(0.25 - 2.63)	—	<0.001	<0.001
Black	1.00	2.42(0.49 - 12.00)	0.71(0.23 - 2.13)	—		
Other	1.00	1.21(0.25 - 5.81)	1.71(0.77 - 3.79)	—		

Cox models were adjusted for gender, age, race, BMI, alcohol, smoking status, education, the Townsend deprivation index, healthy diet score, labor work level, and baseline diseases (including hypertension, diabetes, CVD, and cancer). "—":unstable or inestimable due to few events in the subgroup.

Supplementary Table 36. Stratified Analysis of Consecutive night shifts and CKD Risk by covariates

Characteristics	Consecutive night shifts				*P* value	*P* interaction
	None	<2 shifts/month	2-7shifts/month	>7shifts/month		
Sex						
Women	1.00	1.11(0.81 - 1.51)	1.26(1.08 - 1.48)	1.27(0.88 - 1.85)	0.004	0.68
Man	1.00	0.94(0.71 - 1.25)	1.16(1.02 - 1.33)	1.30(0.99 - 1.72)		
Sleep duration						
≤6 h/day	1.00	0.98(0.65 - 1.47)	1.10(0.90 - 1.35)	1.29(0.86 - 1.93)	0.001	0.26
7-8 h/day	1.00	1.09(0.85 - 1.39)	1.23(1.08 - 1.39)	1.33(1.02 - 1.75)		
≥9 h/day	1.00	0.21(0.03 - 1.50)	1.38(0.90 - 2.13)	0.87(0.27 - 2.82)		
Age						
<60years	1.00	0.96(0.73 - 1.25)	1.23(1.08 - 1.39)	1.36(1.02 - 1.81)	0.001	0.74
>=60years	1.00	1.07(0.77 - 1.49)	1.10(0.92 - 1.31)	1.23(0.87 - 1.73)		
Smoking						
Never	1.00	1.11(0.84 - 1.48)	1.13(0.97 - 1.31)	1.53(1.12 - 2.09)	<0.001	0.22
Previous	1.00	0.83(0.58 - 1.18)	1.32(1.13 - 1.55)	1.11(0.79 - 1.57)		
Current	1.00	1.34(0.75 - 2.37)	1.11(0.79 - 1.55)	1.32(0.64 - 2.72)		
Body mass index						
<18.5	1.00	—	0.80(0.10 - 6.74)	—	0.009	0.82
18.5-25	1.00	1.30(0.90 - 1.89)	1.33(1.07 - 1.65)	1.22(0.72 - 2.08)		
25-30	1.00	1.03(0.75 - 1.41)	1.16(1.00 - 1.36)	1.34(0.96 - 1.88)		
>30	1.00	0.80(0.53 - 1.21)	1.19(1.00 - 1.42)	1.36(0.96 - 1.93)		
Labor work						
Never	1.00	1.08(0.84 - 1.38)	1.21(1.07 - 1.38)	1.42(1.08 - 1.86)		
Sometime	1.00	0.77(0.47 - 1.26)	1.30(1.05 - 1.60)	1.11(0.70 - 1.76)	0.003	0.31
Usually	1.00	0.61(0.22 - 1.67)	0.93(0.62 - 1.41)	0.85(0.31 - 2.34)		
Always	1.00	1.76(0.82 - 3.76)	0.95(0.59 - 1.54)	1.37(0.57 - 3.26)		
Healthy diet						
<4	1.00	1.04(0.81 - 1.34)	1.30(1.15 - 1.47)	1.25(0.95 - 1.65)	<0.001	0.31
>=4	1.00	0.99(0.68 - 1.42)	1.03(0.86 - 1.25)	1.42(0.98 - 2.05)		
Chronotype						
Morning	1.00	0.91(0.58 - 1.41)	1.19(0.97 - 1.46)	1.40(0.93 - 2.10)	<0.001	0.46
Intermediate	1.00	1.03(0.80 - 1.34)	1.25(1.11 - 1.42)	1.34(1.01 - 1.76)		
Evening	1.00	1.21(0.68 - 2.13)	1.02(0.73 - 1.44)	0.90(0.42 - 1.92)		
Alcohol						
Never	1.00	1.67(0.51 - 5.48)	1.12(0.55 - 2.24)	1.35(0.31 - 5.93)	<0.001	0.75
Previous	1.00	0.63(0.15 - 2.63)	1.66(0.98 - 2.81)	1.40(0.49 - 3.98)		

Characteristics	Consecutive night shifts				*P* value	*P* interaction
	None	<2 shifts/month	2-7shifts/month	>7shifts/month		
Current	1.00	1.03(0.83 - 1.27)	1.20(1.08 - 1.33)	1.30(1.04 - 1.63)		
Education						
College or University degree	1.00	0.97(0.71 - 1.32)	1.16(0.98 - 1.37)	1.46(1.03 - 2.07)	<0.001	0.75
Other	1.00	1.06(0.80 - 1.40)	1.24(1.09 - 1.41)	1.22(0.92 - 1.62)		
Cancer						
Yes	1.00	1.10(0.61 - 1.98)	0.97(0.70 - 1.35)	0.64(0.26 - 1.56)	<0.001	0.21
No	1.00	1.01(0.81-1.26)	1.24(1.11-1.38)	1.40(1.12-1.76)		
Hypertension						
Yes	1.00	1.13(0.81 - 1.59)	1.27(1.08 - 1.50)	1.06(0.73 - 1.54)	0.004	0.35
No	1.00	0.96(0.74-1.25)	1.17(1.02-1.33)	1.49(1.13-1.96)		
Diabetes						
Yes	1.00	1.05(0.56 - 1.96)	1.08(0.77 - 1.52)	1.13(0.55 - 2.33)	<0.001	0.86
No	1.00	1.01(0.81-1.26)	1.22(1.10-1.36)	1.34(1.06-1.69)		
Cardiovascular disease					<0.001	<0.001
Yes	1.00	0.78(0.34 - 1.79)	1.06(0.75 - 1.47)	1.54(0.85 - 2.81)		
No	1.00	1.04(0.84-1.29)	1.22(1.10-1.36)	1.27(1.01-1.62)		
Ethnic						
White	1.00	0.99(0.80 - 1.23)	1.22(1.10 - 1.35)	1.35(1.09 - 1.69)		
Asian	1.00	—	1.13(0.35 - 3.59)	0.78(0.09 - 6.52)	<0.001	<0.001
Black	1.00	2.11(0.43 - 10.42)	0.88(0.28 - 2.72)	—		
Other	1.00	3.07(0.98 - 9.63)	1.37(0.59 - 3.19)	—		

Cox models were adjusted for gender, age, race, BMI, alcohol, smoking status, education, the Townsend deprivation index, healthy diet score, labor work level, and baseline diseases (including hypertension, diabetes, CVD, and cancer). "—":unstable or inestimable due to few events in the subgroup.

Supplementary Figure 6. Stratified Analysis of Current night shift work and CKD Risk by PRS Group .

Group	No.Total	No.Case	HR(95%CI)	P value
**Low genetic risk**				
Never/rarely night shifts	57519	1984	1.00 (ref.)	
Irregular shift workers	4167	173	1.18(1.01-1.39)	0.034
Permanent night shift workers	1420	59	1.13 (0.87-1.46)	0.373
**Intermediate genetic risk**				
Never/rarely night shifts	115227	4663	1.18 (1.12-1.24)	< 0.001
Irregular shift workers	8232	256	1.23 (1.10-1.38)	< 0.001
Permanent night shift workers	2753	140	1.37 (1.15-1.63)	< 0.001
**High genetic risk**				
Never/rarely night shifts	57550	2886	1.48 (1.40-1.57)	< 0.001
Irregular shift workers	4165	214	1.46 (1.26-1.68)	< 0.001
Permanent night shift workers	1392	98	1.89 (1.54-2.32)	< 0.001

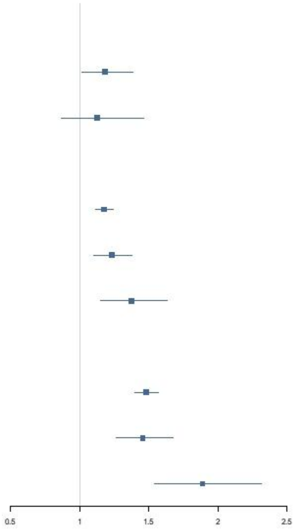

Cox models were adjusted for gender, age, race, BMI, alcohol, smoking status, education, the Townsend deprivation index, healthy diet score, labor work level, and baseline diseases (including hypertension, diabetes, CVD, and cancer).

Supplementary Figure 7. Risk of incident CKD according to Period spent working night shifts and genetic risk base on the SNPs from Yu et al.

Group	No.Total	No.Case	HR(95%CI)	P value
**Low genetic risk**				
None	12803	339	1.00(ref.)	
<2 years	1406	54	1.35 (1.01-1.81)	0.039
2-14years	2229	99	1.53 (1.22-1.92)	< 0.001
>14 years	539	11	0.68 (0.37-1.24)	0.21
**Intermediate genetic risk**				
None	25692	811	1.20 (1.05-1.36)	0.006
<2 years	3155	118	1.33 (1.08-1.64)	0.008
2-14years	4164	167	1.41 (1.17-1.70)	< 0.001
>14 years	942	45	1.68 (1.23-2.30)	0.001
**High genetic risk**				
None	12903	542	1.63 (1.42-1.87)	< 0.001
<2 years	1587	83	1.84 (1.44-2.34)	< 0.001
2-14years	2055	103	1.72 (1.38-2.15)	< 0.001
>14 years	432	24	1.85 (1.22-2.80)	0.004

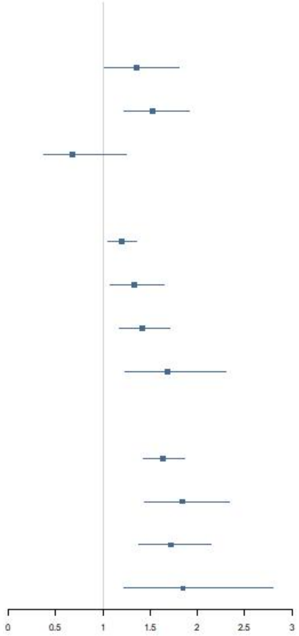

Cox models were adjusted for gender, age, race, BMI, alcohol, smoking status, education, the Townsend deprivation index, healthy diet score, labor work level, and baseline diseases (including hypertension, diabetes, CVD, and cancer).

Supplementary Figure 8. Stratified Analysis of Night shifts worked monthly and CKD Risk by PRS Group base on the SNPs from Yu et al.

Group	No.Total	No.Case	HR(95%CI)	P value
**Low genetic risk**				
None	12803	339	1.00(ref.)	
<5/month	1150	35	1.08 (0.76-1.53)	0.669
5-10/month	1936	74	1.38 (1.07-1.77)	0.013
>10/month	1088	55	1.59 (1.19-2.12)	0.002
**Intermediate genetic risk**				
None	25692	881	1.20 (1.05-1.36)	0.006
<5/month	2264	92	1.48 (1.17-1.86)	< 0.001
5-10/month	3760	143	1.40 (1.15-1.71)	< 0.001
>10/month	2237	95	1.37 (1.09-1.72)	0.008
**High genetic risk**				
None	12903	542	1.63 (1.42-1.87)	< 0.001
<5/month	1128	51	1.60 (1.19-2.16)	0.002
5-10/month	1871	98	1.85 (1.48-2.32)	< 0.001
>10/month	1075	61	1.83 (1.39-2.41)	< 0.001

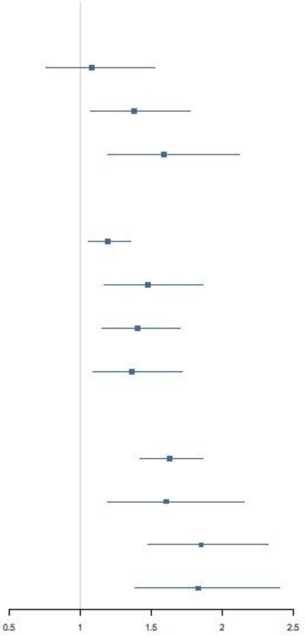

Cox models were adjusted for gender, age, race, BMI, alcohol, smoking status, education, the Townsend deprivation index, healthy diet score, labor work level, and baseline diseases (including hypertension, diabetes, CVD, and cancer).

Supplementary Figure 9. Stratified Analysis of Usual length of each night shift and CKD Risk by PRS Group base on the SNPs from Yu et al.

Group	No.Total	No.Case	HR(95%CI)	P value
**Low genetic risk**				
None	12803	339	1.00(ref.)	
<8h	453	27	1.92 (1.30-2.85)	0.001
8-12h	3424	131	1.32 (1.08-1.62)	0.008
>12h	297	6	0.82 (0.37-1.84)	0.627
**Intermediate genetic risk**				
None	25692	811	1.20 (1.05-1.36)	0.006
<8h	954	51	1.83 (1.36-2.45)	< 0.001
8-12h	6736	262	1.36 (1.16-1.60)	< 0.001
>12h	571	17	1.25 (0.77-2.04)	0.362
**High genetic risk**				
None	12903	542	1.63 (1.42-1.87)	< 0.001
<8h	418	24	2.00 (1.32-3.04)	0.001
8-12h	3332	175	1.78 (1.48-2.14)	< 0.001
>12h	324	11	1.39 (0.76-2.54)	0.279

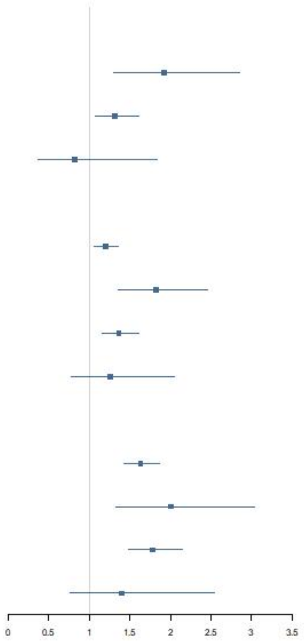

Cox models were adjusted for gender, age, race, BMI, alcohol, smoking status, education, the Townsend deprivation index, healthy diet score, labor work level, and baseline diseases (including hypertension, diabetes, CVD, and cancer).

Supplementary Figure 10. Stratified Analysis of Consecutive night shifts and CKD Risk by PRS Group base on the SNPs from Yu et al.

Group	No.Total	No.Case	HR(95%CI)	P v
**Low genetic risk**				
None	12803	339	1.00 (ref.)	
<2shifts	652	13	0.72 (0.41-1.26)	0.248
2-7shifts	3113	133	1.48 (1.21-1.82)	< 0.001
>7shifts	409	18	1.38 (0.86-2.22)	0.182
**Intermediate genetic risk**				
None	25692	811	1.20 (1.05-1.36)	0.006
<2shifts	1381	48	1.30 (0.96-1.76)	0.086
2-7shifts	6088	240	1.40 (1.18-1.65)	< 0.001
>7shifts	792	42	1.67 (1.21-2.30)	0.002
**High genetic risk**				
None	12903	542	1.63 (1.42-1.87)	< 0.001
<2shifts	718	34	1.75 (1.23-2.50)	0.002
2-7shifts	2943	151	1.78 (1.46-2.16)	< 0.001
>7shifts	413	25	1.84 (1.22-2.76)	0.003

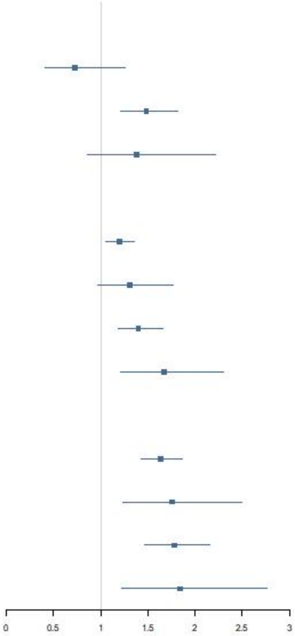

Cox models were adjusted for gender, age, race, BMI, alcohol, smoking status, education, the Townsend deprivation index, healthy diet score, labor work level, and baseline diseases (including hypertension, diabetes, CVD, and cancer).

Supplementary Table 37 . Sensitivity analysis of the joint association between PRS and Current employment status on CKD risk: an additive-scale analysis

Group	RERI (95% CI)	AP (95% CI)	*P* 值	Interaction Type
**Intermediate genetic risk**				
Irregular shift workers	-0.13 (-0.22, 0.07)	-0.11 (-0.19, 0.05)	0.200	Additive
Permanent night shift workers	0.07 (-0.16, 0.51)	0.05 (-0.13, 0.34)	0.617	Additive
**High genetic risk**				
Irregular shift workers	-0.21(-0.29, -0.04)	-0.14(-0.21, -0.03)	0.037	Sub-additive*
Permanent night shift workers	0.28 (0.07, 0.88)	0.15 (0.04, 0.40)	0.021	Super-additive*

RERI and AP were used to assess the departure from additivity. P-values represent the significance of RERI.

Interaction types (Additive, Super-additive, or Sub-additive) were defined as per the criteria detailed in Table S12.

Supplementary Table 38 . Sensitivity analysis of the joint association between PRS and Period spent working night shifts on CKD risk: an additive-scale analysis

Group	RERI (95% CI)	AP (95% CI)	P value	Interaction Type
**Intermediate genetic risk**				
<2years	-0.22 (-0.74, 0.14)	0.17 (-0.61, 0.11)	0.293	Additive
2-14years	-0.31 (-0.71, -0.07)	-0.22 (-0.51, -0.06)	0.045	Sub-additive*
>14years	0.81 (-0.04, 1.30)	0.48 (-0.03, 0.65)	0.063	Additive
**High genetic risk**				
<2years	-0.15 (-0.71, 0.25)	-0.08 (-0.40, 0.14)	0.506	Additive
2-14years	-0.44 (-1.02, 0.04)	-0.26 (-0.66, 0.02)	0.068	Additive
>14years	0.54 (-0.41, 1.48)	0.29 (-0.29, 0.58)	0.202	Additive

RERI and AP were used to assess the departure from additivity. P-values represent the significance of RERI.
Interaction types (Additive, Super-additive, or Sub-additive) were defined as per the criteria detailed in Table S12.

Supplementary Table 39. Sensitivity analysis of the joint association between PRS and Number of night shifts worked monthly on CKD risk: an additive-scale analysis

Group	RERI (95% CI)	AP (95% CI)	P value	Interaction Type
**Intermediate genetic risk**				
<5/month	0.20 (-0.40, 0.60)	0.14 (-0.27, 0.37)	0.420	Additive
5-10/month	-0.17 (-0.65, 0.15)	-0.12 (-0.60, 0.09)	0.402	Additive
>10/month	-0.42 (-1.03, -0.03)	-0.31 (-0.78, -0.02)	0.043	Sub-additive*
**High genetic risk**				
<5/month	-0.11 (-0.87, 0.26)	-0.07 (-0.63, 0.15)	0.414	Additive
5-10/month	-0.16 (-0.70, 0.40)	-0.08 (-0.48, 0.17)	0.479	Additive
>10/month	-0.39 (-1.20, 0.21)	-0.21 (-0.76, 0.12)	0.174	Additive

RERI and AP were used to assess the departure from additivity. P-values represent the significance of RERI. Interaction types (Additive, Super-additive, or Sub-additive) were defined as per the criteria detailed in Table S12.

Supplementary Table 40. Sensitivity analysis of the joint association between PRS and Usual length of each night shift on CKD risk: an additive-scale analysis

Group	RERI (95% CI)	AP (95% CI)	P value	Interaction Type
**Intermediate genetic risk**				
<8 h	-0.30 (-1.10, 0.24)	-0.16 (-0.63, 0.14)	0.387	Additive
8-12h	-0.15 (-0.50, 0.20)	-0.11 (-0.41, 0.13)	0.399	Additive
>12h	0.24 (-0.60, 0.89)	0.19 (-0.61, 0.62)	0.527	Additive
**High genetic risk**				
<8 h	-0.55 (-1.86, 0.24)	-0.28 (-1.30, 0.10)	0.303	Additive
8-12h	-0.17 (-0.61, 0.26)	-0.09 (-0.35, 0.12)	0.453	Additive
>12h	-0.06 (-1.33, 1.14)	-0.04 (-1.60, 0.56)	0.930	Additive

RERI and AP were used to assess the departure from additivity. P-values represent the significance of RERI.

Interaction types (Additive, Super-additive, or Sub-additive) were defined as per the criteria detailed in Table S12.

Supplementary Table 41. Sensitivity analysis of the joint association between PRS and Consecutive night shifts on CKD risk: an additive-scale analysis

Group	RERI (95% CI)	AP (95% CI)	*P* value	Interaction Type
**Intermediate genetic risk**				
<2 shifts/month	0.39 (-0.30, 0.91)	0.30 (-0.32, 0.58)	0.312	Additive
2-7shifts/month	-0.28 (-0.67, 0.04)	-0.20 (-0.55, 0.02)	0.087	Additive
>7shifts/month	0.09 (-0.78, 0.65)	0.06 (-0.53, 0.36)	0.802	Additive
**High genetic risk**				
<2 shifts/month	0.40 (-0.42, 0.92)	0.23 (-0.26, 0.44)	0.340	Additive
2-7shifts/month	-0.34 (-0.83, 0.14)	-0.19 (-0.50, 0.07)	0.172	Additive
>7shifts/month	-0.17 (-1.60, 0.52)	-0.10 (-1.14, 0.22)	0.748	Additive

RERI and AP were used to assess the departure from additivity. P-values represent the significance of RERI. Interaction types (Additive, Super-additive, or Sub-additive) were defined as per the criteria detailed in Table S12.

Supplementary Figure 11. Associations between the Current or lifelong employment status and risks of CKD using competing risk models

Group	No.Total	No.Case	HR(95%CI)
**current work schedule**			
Never/rarely night shifts	230296	9533	1.00(ref.)
Irregular night shift	16564	743	1.07(0.99-1.15)
Permanent night shift	5565	294	1.20(1.07-1.36)
**Period spent working night shifts**			
None	51398	1692	1.00(ref.)
<2years	6148	255	1.17(1.02-1.34)
2-14years	8448	369	1.21(1.08-1.36)
>14years	1913	80	1.15(0.92-1.44)
**Number of night shifts worked monthly**			
None	51389	1692	1.00(ref.)
<5/month	4542	178	1.12(0.96-1.31)
5-10/month	7567	315	1.21(1.07-1.37)
>10/month	4400	211	1.22(1.06-1.42)
**Usual length of each night shift**			
None	51398	1692	1.00(ref.)
<8 h	1825	102	1.50(1.23-1.84)
8-12h	13492	568	1.16(1.06-1.28)
>12h	1192	34	0.94(0.67-1.32)
**Consecutive night shifts**			
None	51398	1692	1.00(ref.)
<2 shifts/month	2751	95	1.02(0.83-1.25)
2-7shifts/month	12144	524	1.21(1.09-1.34)
>7shifts/month	1614	85	1.31(1.05-1.63)

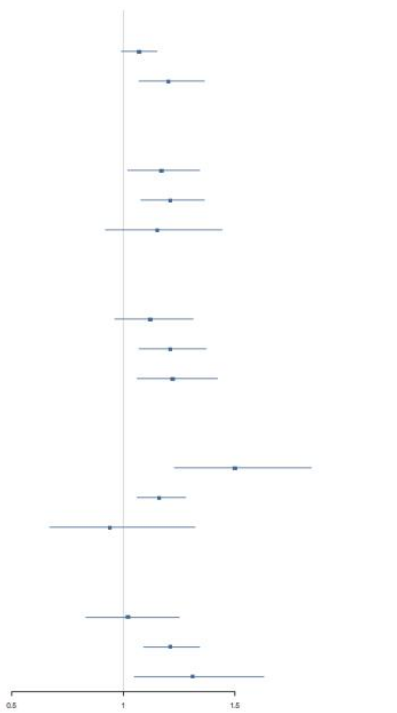

Cox models were adjusted for gender, age, race, BMI, alcohol, smoking status, education, the Townsend deprivation index, healthy diet score, labor work level, and baseline diseases (including hypertension, diabetes, CVD, and cancer).

Supplementary Table 42. Associations between the pre-baseline Period spent working night shifts and risks of CKD (n=67881)

	Period spent working night shifts				
	None	<2years	2-14years	>14years	
No. participants	51376	3550	9315	3640	
No. Cases (n, %)	1690 (3.29)	139 (3.92)	423 (4.54)	142 (3.90)	
	HR	HR (95% CI)	HR (95% CI)	HR (95% CI)	[d]*P* value
Unadjusted model	1.00 (ref.)	1.37(1.20-1.57)	1.36(1.20-1.53)	1.18(1.00-1.41)	< 0.001
[a]Model 1	1.00 (ref.)	1.29 (1.09-1.54)	1.42 (1.28-1.58)	1.16 (0.97-1.37)	< 0.001
[b]Model 2	1.00 (ref.)	1.18 (1.00-1.41)	1.31 (1.18-1.46)	1.06 (0.90-1.27)	< 0.001
[c]Model 3	1.00 (ref.)	1.16 (0.97-1.38)	1.27 (1.14-1.41)	1.02 (0.86-1.22)	0.003

[a]Model 1:adjusted for gender, age and race;

[b]Model 2: adjusted for model 1 plus BMI, alcohol , smoking status, education, and the Townsend deprivation index;

[c]Model 3: adjusted for model 2 plus healthy diet score, labor work level, and baseline diseases (including hypertension, diabetes, CVD, and cancer).

[d]*P* values: represent the overall significance of the categorical variable, calculated using the Likelihood Ratio Test (LRT) comparing models with and without the variable.

Supplementary Table 43. Associations between the pre-baseline Number of night shifts worked monthly and risks of CKD (n=67881)

	Number of night shifts worked monthly				
	None	<5/month	5-10/month	>10/month	
No. participants	51376	4541	7565	4399	
No. Cases (n, %)	1690 (3.29)	178 (3.92)	315 (4.16)	211 (4.80)	
	HR	HR (95% CI)	HR (95% CI)	HR (95% CI)	[d]*P* value
Unadjusted model	1.00 (ref.)	1.20 (1.03-1.40)	1.28 (1.13-1.44)	1.47 (1.27-1.69)	< 0.001
[a]Model 1	1.00 (ref.)	1.22 (1.05-1.43)	1.34 (1.18-1.51)	1.44 (1.25-1.66)	< 0.001
[b]Model 2	1.00 (ref.)	1.15 (0.99-1.35)	1.24 (1.10-1.40)	1.28 (1.11-1.48)	< 0.001
[c]Model 3	1.00 (ref.)	1.12 (0.96-1.32)	1.21 (1.06-1.36)	1.23 (1.06-1.42)	0.003

[a]Model 1:adjusted for gender, age and race;

[b]Model 2: adjusted for model 1 plus BMI, alcohol , smoking status, education, and the Townsend deprivation index;

[c]Model 3: adjusted for model 2 plus healthy diet score, labor work level, and baseline diseases (including hypertension, diabetes, CVD, and cancer).

[d]*P* values: represent the overall significance of the categorical variable, calculated using the Likelihood Ratio Test (LRT) comparing models with and without the variable.

Supplementary Table 44. Associations between the pre-baseline Usual length of each night shift and risks of CKD (n=67881)

	Usual length of each night shift				
	None	<8h	8-12h	>12h	
No. participants	51376	1823	13490	1192	
No. Cases (n, %)	1690 (3.29)	102 (5.60)	568 (4.21)	34 (2.85)	
	HR	HR (95% CI)	HR (95% CI)	HR (95% CI)	[d]*P* value
Unadjusted model	1.00 (ref.)	1.73(1.42-2.12)	1.29(1.17-1.42)	0.86(0.62-1.21)	< 0.001
[a]Model 1	1.00 (ref.)	1.67 (1.37-2.05)	1.31 (1.19-1.45)	0.96 (0.69-1.35)	< 0.001
[b]Model 2	1.00 (ref.)	1.54 (1.26-1.88)	1.21 (1.10-1.33)	0.93 (0.66-1.31)	< 0.001
[c]Model 3	1.00 (ref.)	1.51 (1.23-1.85)	1.16 (1.05-1.29)	0.94 (0.67-1.33)	< 0.001

[a]Model 1:adjusted for gender, age and race;

[b]Model 2: adjusted for model 1 plus BMI, alcohol , smoking status, education, and the Townsend deprivation index;

[c]Model 3: adjusted for model 2 plus healthy diet score, labor work level, and baseline diseases (including hypertension, diabetes, CVD, and cancer).

[d]*P* values: represent the overall significance of the categorical variable, calculated using the Likelihood Ratio Test (LRT) comparing models with and without the variable.

Supplementary Table 45. Associations between the pre-baseline Consecutive night shifts and risks of CKD (n=67881)

	Consecutive night shifts				
	None	<2shifts/month	2-7shifts/month	>7shifts/month	
No. participants	51376	2750	12142	1613	
No. Cases (n, %)	1690 (3.29)	95 (3.45)	524 (4.32)	85 (5.27)	
	HR	HR (95% CI)	HR (95% CI)	HR (95% CI)	[d]*P* value
Unadjusted model	1.00 (ref.)	1.05 (0.85-1.29)	1.32 (1.20-1.46)	1.62 (1.30-2.02)	< 0.001
[a]Model 1	1.00 (ref.)	1.07 (0.87-1.32)	1.36 (1.24-1.51)	1.52 (1.22-1.89)	< 0.001
[b]Model 2	1.00 (ref.)	1.03 (0.84-1.27)	1.25 (1.13-1.39)	1.36 (1.09-1.69)	< 0.001
[c]Model 3	1.00 (ref.)	1.02 (0.83-1.26)	1.21 (1.09-1.34)	1.31 (1.05-1.63)	< 0.001

[a]Model 1:adjusted for gender, age and race;

[b]Model 2: adjusted for model 1 plus BMI, alcohol , smoking status, education, and the Townsend deprivation index;

[c]Model 3: adjusted for model 2 plus healthy diet score, labor work level, and baseline diseases (including hypertension, diabetes, CVD, and cancer).

[d]*P* values: represent the overall significance of the categorical variable, calculated using the Likelihood Ratio Test (LRT) comparing models with and without the variable.

1.Wuttke M, Li Y, Li M, Sieber KB, Lifelines Cohort Study, VA Million Veteran Program, et al. A catalog of genetic loci associated with kidney function from analyses of a million individuals. Nat Genet. 2019;51(6):957–72. doi:10.1038/s41588-019-0407-x
2.Yu Z, Jin J, Tin A, Köttgen A, Yu B, Chen J, et al. Polygenic risk scores for kidney function and their associations with circulating proteome and incident kidney diseases. J Am Soc Nephrol. 2021;32(12):3025–39. doi:10.1681/ASN.2020111599
