# Supplementary material for: Association between night shift work, genetic risk, and chronic kidney disease: a prospective cohort study based on the UK Biobank
Source: Front Public Health. 2026 Apr 30;14:1772746. doi: 10.3389/fpubh.2026.1772746 (PMC13171536; doi:10.3389/fpubh.2026.1772746)
Supplement: Supplementary file 3 [file Table_1.doc]

STROBE Statement—Checklist of items that should be included in reports of ***cohort studies***

|  | Item No | Recommendation | **Location (Page No.)** |
| --- | --- | --- | --- |
| **Title and abstract** | 1 | (*a*) Indicate the study’s design with a commonly used term in the title or the abstract | Page1- 2 |
|  |  | (*b*) Provide in the abstract an informative and balanced summary of what was done and what was found | Page 2 |
| Introduction | | |  |
| Background/rationale | 2 | Explain the scientific background and rationale for the investigation being reported | Page3 |
| Objectives | 3 | State specific objectives, including any prespecified hypotheses | Page4 |
| Methods | | |  |
| Study design | 4 | Present key elements of study design early in the paper | Page4(Prospective cohort study design) |
| Setting | 5 | Describe the setting, locations, and relevant dates, including periods of recruitment, exposure, follow-up, and data collection | Page4(UKB recruitment) and  Page6(Follow-up end dates) |
| Participants | 6 | (*a*) Give the eligibility criteria, and the sources and methods of selection of participants. Describe methods of follow-up | Page4(Exclusion of prevalent CKD; Selection of 75,391 sub-cohort) |
|  |  | (*b*) For matched studies, give matching criteria and number of exposed and unexposed | N/A(No matching was performed in this study) |
| Variables | 7 | Clearly define all outcomes, exposures, predictors, potential confounders, and effect modifiers. Give diagnostic criteria, if applicable | Page5-8(Definitions of incident CKD, night shift work, and covariates) |
| Data sources/ measurement | 8* | For each variable of interest, give sources of data and details of methods of assessment (measurement). Describe comparability of assessment methods if there is more than one group | Page7(Questionnaires,EHR linkage and Lab analysis);  Page4(Comparability between 2006 baseline and 2015 sub-cohort questionnaires) |
| Bias | 9 | Describe any efforts to address potential sources of bias | Page4(Exclusion of prevalent CKD;Page6(Use of linked EHR and Lab analysis to minimize outcome misclassification);Page9(Multivariate Cox model adjusts for covariates);Page11(Sensitivity analyses and truncation to address immortal-time bias) |
| Study size | 10 | Explain how the study size was arrived at | page 4(Sample size was determined by the maximum available participants after applying exclusion criteria; detailed in Figure 1) |
| Quantitative variables | 11 | Explain how quantitative variables were handled in the analyses. If applicable, describe which groupings were chosen and why | page 7(Rationale for handling variables as continuous or categorical to assess linear/non-linear associations) |
| Statistical methods | 12 | (*a*) Describe all statistical methods, including those used to control for confounding | page 9(Use of Cox models with stepwise adjustment for confounders in Models 1–3)  Page10(Mediation analysis via effect decomposition; Joint models for Gene-Environment analysis) |
|  |  | (*b*) Describe any methods used to examine subgroups and interactions | page 11(Subgroup analyses by age/sex and use of interaction terms to assess effect modification) |
|  |  | (*c*) Explain how missing data were addressed | Page 10 (Assessment of multiplicative interaction via cross-product terms and additive interaction via RERI and AP) |
|  |  | (*d*) If applicable, explain how loss to follow-up was addressed | Page 6 (Handling of loss to follow-up via right censoring in the follow-up definition section) |
|  |  | (*e*) Describe any sensitivity analyses | Page 11 (Sensitivity analyses including excluding early CKD cases and addressing immortal-time bias) |
| Results | | |  |
| Participants | 13* | (a) Report numbers of individuals at each stage of study—eg numbers potentially eligible, examined for eligibility, confirmed eligible, included in the study, completing follow-up, and analysed | Page 12 (Current status cohort n=252,425; Lifetime exposure sub-cohort n=75,391; see Figure 1). |
|  |  | (b) Give reasons for non-participation at each stage | Page 12 (Reasons for exclusion: prevalent CKD at baseline and missing data) |
|  |  | (c) Consider use of a flow diagram | Figure1 |
| Descriptive data | 14* | (a) Give characteristics of study participants (eg demographic, clinical, social) and information on exposures and potential confounders | Page 13(Demographic and clinical profiles by night shift status; see Table 2 and Table S5-8) |
|  |  | (b) Indicate number of participants with missing data for each variable of interest | Page 13 (Reference to Table S4 for specific missing counts) |
|  |  | (c) Summarise follow-up time (eg, average and total amount) | Page 13 (Median follow-up: 13.7 years (IQR, 13.0–14.3 years) ) |
| Outcome data | 15* | Report numbers of outcome events or summary measures over time | Page 13 (Total: 3.36 million person-years; 10,573 and 2,396 incident CKD cases) |
| Main results | 16 | (*a*) Give unadjusted estimates and, if applicable, confounder-adjusted estimates and their precision (eg, 95% confidence interval). Make clear which confounders were adjusted for and why they were included | Page 14,Tables 2-6 (Unadjusted HRs and hierarchical adjustments in Models 1–3 are detailed with 95% CIs) |
|  |  | (*b*) Report category boundaries when continuous variables were categorized | Page 8, Page12, Figure S1 (Specific boundaries for duration, frequency, length, and consecutive shifts are defined) |
|  |  | (*c*) If relevant, consider translating estimates of relative risk into absolute risk for a meaningful time period | Page 10,Page 13-14, Tables 2–6 (Both incidence rates per 1,000 person-years and 10-year cumulative risks are reported to provide absolute risk measures) |
| Other analyses | 17 | Report other analyses done—eg analyses of subgroups and interactions, and sensitivity analyses | Page17- 18, Tables S5–S36，Figures2 ,S3-11 (Interaction tests across subgroups and sensitivity analyses ) |
| Discussion | | |  |
| Key results | 18 | Summarise key results with reference to study objectives | Page 19 (Overall associations and specific risk estimates for permanent shifts are summarized) |
| Limitations | 19 | Discuss limitations of the study, taking into account sources of potential bias or imprecision. Discuss both direction and magnitude of any potential bias | Page 24 (The impact of healthy volunteer bias, recall bias, and under-ascertainment on the direction of HRs is discussed) |
| Interpretation | 20 | Give a cautious overall interpretation of results considering objectives, limitations, multiplicity of analyses, results from similar studies, and other relevant evidence | Page 19-24 (A balanced interpretation considering study limitations, consistency with prior evidence, and potential biological mechanisms is provided) |
| Generalisability | 21 | Discuss the generalisability (external validity) of the study results | Page 24(The potential impact of ethnic homogeneity and selection bias on external validity is summarized) |
| Other information | | |  |
| Funding | 22 | Give the source of funding and the role of the funders for the present study and, if applicable, for the original study on which the present article is based | Page 25(Sources of research support and the independence of funders are declared) |

*Give information separately for exposed and unexposed groups.

**Note:** An Explanation and Elaboration article discusses each checklist item and gives methodological background and published examples of transparent reporting. The STROBE checklist is best used in conjunction with this article (freely available on the Web sites of PLoS Medicine at http://www.plosmedicine.org/, Annals of Internal Medicine at http://www.annals.org/, and Epidemiology at http://www.epidem.com/). Information on the STROBE Initiative is available at http://www.strobe-statement.org.
